# Barriers to the application of Health Technology Assessment (HTA) results: the case of COVID-19 vaccine deployment in Ghana

**DOI:** 10.1017/S0266462325100342

**Published:** 2026-02-02

**Authors:** Brian Adu Asare, Emmanuella Abassah-Konadu, Sarah Ama Kafui Okine, Ivy Amankwah, Desmond Dzidzornu Otoo, Hannah Asante, Saviour Yevutsey, Maxwell Ayindenaba Dalaba, Mustapha Immurana, Matilda Aberese-Ako, Joycelyn Azeez, Edith Enyonam Gavor, Martha Gyansa-Lutterodt, Evelyn Ansah, Tuoyo Okorosobo, Olumide Ogundahunsi, Abraham Hodgson, Seth Owusu Agyei, Margaret Gyapong

**Affiliations:** 1 Health Technology Assessment Secretariat, Ministry of Health, Ghana; 2 Ministry of Health, Ghana; 3 Health Technology Assessment Technical Working Group, Ministry of Health, Ghana; 4 University of Health and Allied Sciences, Ghana; 5 Public Health Specialist, Ghana; 6 Ghana College of Pharmacists, Public Health, Ghana; 7 SAVING Consortium

**Keywords:** Access and Delivery, Health Technology Assessment, barriers, COVID-19, vaccine deployment, public health emergencies, policy recommendations, Ghana

## Abstract

**Objectives:**

Health Technology Assessment (HTA) guides healthcare decision-making, while Implementation Research (IR) addresses challenges in operationalizing these decisions. The SAVING (Sustainable Access and Delivery of New Vaccines in Ghana) Consortium aims to enhance health intervention delivery in Ghana, focusing on HTA evidence. This study identifies barriers to the application of HTA-related evidence (cost analysis) in coronavirus disease 2019 (COVID-19) vaccine deployment in Ghana.

**Methods:**

This qualitative exploratory study purposively selected 12 key stakeholders with high interest and power relating to COVID-19 vaccine deployment in Ghana. Through in-depth interviews, seven stakeholders from diverse sectors contributed insights into barriers to the application of HTA-related evidence. Thematic analysis was conducted with narrative reporting supported by direct quotes for substantiation.

**Results:**

Six main barriers were identified: (1) timing and access to HTA reports, (2) technical complexities, (3) relevance of content, (4) political considerations and power dynamics, (5) health system fragmentation, and (6) poor responsiveness of decision-makers to research. Proposed solutions include engaging political decision-makers continuously, simplifying technical reports, aligning report content with policymakers’ needs, reducing political considerations, enhancing capacity building, fostering health system cohesion, and improving responsiveness to research.

**Conclusions:**

HTA is vital for informed healthcare decisions. However, technical complexity, relevance of content, inappropriate timing, and lack of access to HTA reports, among other barriers, prevent the uptake of HTA findings. Continuous and improved engagement between HTA producers and policymakers, along with rapid production of HTA, has the potential to improve the uptake of HTA findings, even during public health emergencies.

## Introduction

Health Technology Assessment (HTA) determines value in the introduction, adoption, and use of health technologies in health systems (1;2) and seeks to inform decision-making (3;4). In translating evidence to practice, Implementation Research (IR) seeks to address bottlenecks and approaches for unique settings. IR can provide data to guide optimal roll-out of interventions assessed through HTA (5). Such interventions can be vaccines, medical devices, procedures, diagnostic tests, systems, and healthcare programs (3). The SAVING (Sustainable Access and Delivery of New Vaccines in Ghana) Consortium (6), made up of the University of Health and Allied Sciences as the lead, the Ministry of Health (MOH), the Ghana Food and Drugs Authority (FDA), the Swiss Tropical and Public Health Institute, collaborating with the World Health Organization (WHO) special program for Research and Training in Tropical Diseases (also known as WHO-TDR), and Programme for Appropriate Technology in Health (PATH)/Access and Delivery Partnership (ADP), aims to enhance health intervention delivery in Ghana, focusing on use of HTA and HTA-related evidence.

The health system in Ghana is governed by a central MOH with administrative and policy formulation functions, while service delivery is by the Ghana Health Service (GHS). Ghana’s MOH institutionalized HTA as a tool for decision-making, in response to the 67th World Health Assembly resolution agenda item 15.7 on HTA (7), toward efficiency improvements in resource use. In 2019, Ghana established an HTA multi-agency steering committee (HTA-SC), an HTA multidisciplinary technical working group (HTA-TWG), and a multi-representational secretariat for HTA (HTA-SEC) with clear terms of reference to govern and manage the conduct and uptake of HTA (8). This is further supported by an HTA strategy, outlining the strategic objectives (9), HTA reference case, setting out methodological standards for HTA (10), and the HTA process guideline, defining a priori steps for producing HTAs to ensure transparency and consistency (11). Thus, Ghana intends to use HTA in policy prioritization, selection of health technologies, pricing strategies for health technologies, as well as procurement of pharmaceuticals and other health technologies, including vaccines (9).

HTA is used to assess the safety, effectiveness, costs, and economic implications, as well as ethical, social, legal, and organizational aspects of health technologies (12;13). WHO and the United Nations Children’s Fund (UNICEF) provide the COVID-19 Vaccine Introduction and deployment Costing tool (CVIC tool) (14;15) (see Supplementary Material 1), for costing coronavirus disease 2019 (COVID-19) vaccine initiatives in countries. This tool was used to cost COVID-19 vaccine deployment in Ghana, addressing the cost dimension of HTA analysis (16;17) (See Supplementary Material 2). The study was based on the government-initiated National Deployment and Vaccination Plan (NDVP) (16) (see Supplementary Material 3), guided by the WHO UNICEF submission and review plan (17). The NDVP is a comprehensive plan by the MOH and GHS, in collaboration with health partners, to protect the population and mitigate the spread of the virus, paving the way for socioeconomic recovery. This included procurement and deployment of COVID-19 vaccines, involving Ghana’s National Immunization Technical Advisory Group (NITAG) and other relevant statutory and regulatory bodies (16).

The CVIC tool aims to guide countries to introduce COVID-19 vaccines into their health systems (18). The tool estimates incremental costs for resource mobilization purposes, including the World Bank’s COVID-19 Fast-Track Loan Facility, and country-level decision-making. It was used to estimate the cost-component of COVID-19 vaccine deployment in Ghana (19) for a target population of 17.5 million. This HTA-related study identified the cost of vaccines accounting for 78–83 percent of total vaccination costs, translating to a cost per fully vaccinated person of $20.9–$26.2, and made recommendations through the HTA system (19;20). Because deployment would cost Ghana about 61–76 percent of the 2021 health sector budget for nonremuneration activities, it was recommended for decision-makers to ensure vaccine cost containment for a sustainable vaccination program. (Supplementary Materials 4 and 5). Before the COVID-19 pandemic, HTA systems in Ghana were established to respond to evidence needs for policymakers, but not under the constraints of public health emergencies like the COVID-19 pandemic. Uptake of HTA recommendations could be seamless, but not always straightforward (21). In an HTA conducted in 2020 for hypertension management (22), the National Medicines Selection Committee (NMSC), integrated the HTA recommendations into Standard Treatment Guidelines, 7th edition 2017 (23), and Essential Medicines List, 7th edition, 2017 (24), with the National Health Insurance Authority (NHIA) updating the National Health Insurance Medicines List (25). Other HTAs in Ghana have been applied satisfactorily, but not in a public health emergency or pandemic setting such as COVID-19. It appeared that integrating insights from the HTA-related analyses on COVID-19 vaccines encountered some barriers, leading to suboptimal application. Also, when the COVID-19 pandemic emerged, Ghana’s HTA system faced the challenge of rapidly responding to the need for timely, relevant, and accessible HTA findings to guide vaccine procurement and deployment decisions, exposing gaps in the existing framework. This study explores the barriers affecting the application of HTA-related evidence on COVID-19 vaccine deployment in Ghana. It seeks to contribute insights on HTA utilization in health policy and practice, particularly during public health crises in Ghana or other similar contexts.

## Methods

### Study design

This study explored the barriers to the application of HTA recommendations in Ghana’s COVID-19 vaccine deployment during a public health emergency. The qualitative approach sought to provide rich insights into this potentially complex and multifaceted issue.

### Sampling

This study adopted non-probability sampling to choose an appropriate purposive sample (26) drawn from a stakeholder analysis. It identified twelve stakeholders, with high interest and high power concerning COVID-19 vaccine deployment, as initial participants, including the MOH, HTA SC, HTA TWG, HTA-SEC, Expanded Programme of Immunization, Presidential Advisory Committee on COVID-19, National Technical Coordination Committee for COVID-19, NITAG, Drug Policy Unit (DPU), NMSC, NHIA, and the FDA, represented by professionals, policy decision-makers, and researchers involved in the generation of evidence, procurement, supply, and deployment of vaccines in Ghana. A snowballing technique was considered to reach any other relevant stakeholder(s). The following stakeholders were included: participants from the Procurement and Supply Directorate of the MOH (PS-MOH), the Policy Planning Monitoring and Evaluation Directorate of the MOH (PPME-MOH), and the Ministry of Finance (MOF). Seven interviews were conducted, using an interview guide with participant consent form (see Supplementary Material 6), with representatives from the DPU, NMSC, the HTA-SEC, HTA-TWG, the PS-MOH, HTA-SC/PPME-MOH, and MOF (see Supplementary Material 7 for Supplementary Table S1: Stakeholder groups and their roles).

### Data collection technique and tools

The interview guide was developed based on a review of the literature on challenges and the application of HTA results (27). Although some stakeholders were hesitant to participate due to the sensitive nature of the subject, the challenge was overcome through continuous emphasis on the anonymity of data and participant identity. In-depth interviews were conducted using open-ended questions, administered by trained researchers, ensuring privacy and lasting about 45 minutes. These were audio-recorded with participant consent, transcribed verbatim, and cross-checked for accuracy within the research team to facilitate thorough data analysis. The transcriptions were captured in Microsoft Word and imported into NVivo 14 (28) for coding and analysis.

### Data analysis

The qualitative analysis was done thematically and involved a multistep process of data familiarization through reading and re-reading the transcripts, initial coding, categorization leading to theme development, and iterative review for alignment with research goals (29). This led to a synthesized narrative, with validity and credibility enhanced through peer debriefing sessions. To ensure anonymity, the positions of the participants under the quotes have been listed under three-character identifiers.

## Results

The study found several barriers to the application of HTA results during the deployment of COVID-19 vaccines in Ghana. One major barrier was the timeliness and accessibility to reports. Delays in evidence generation meant that by the time evidence was available, it was no longer applicable. Other barriers include the relevance of content, where there was a perception of a disconnect between findings and the immediate needs of the pandemic. Also, it was noted that the report was technical and complex, making it difficult for non-experts in the HTA subject to understand and use for decision-making. Political considerations and power dynamics with political decisions often outweighing scientific recommendations, health system fragmentation with different sectors and departments operating in silos, and low responsiveness to research findings due to affordability, were further barriers identified in the study.

**Figure 1.**
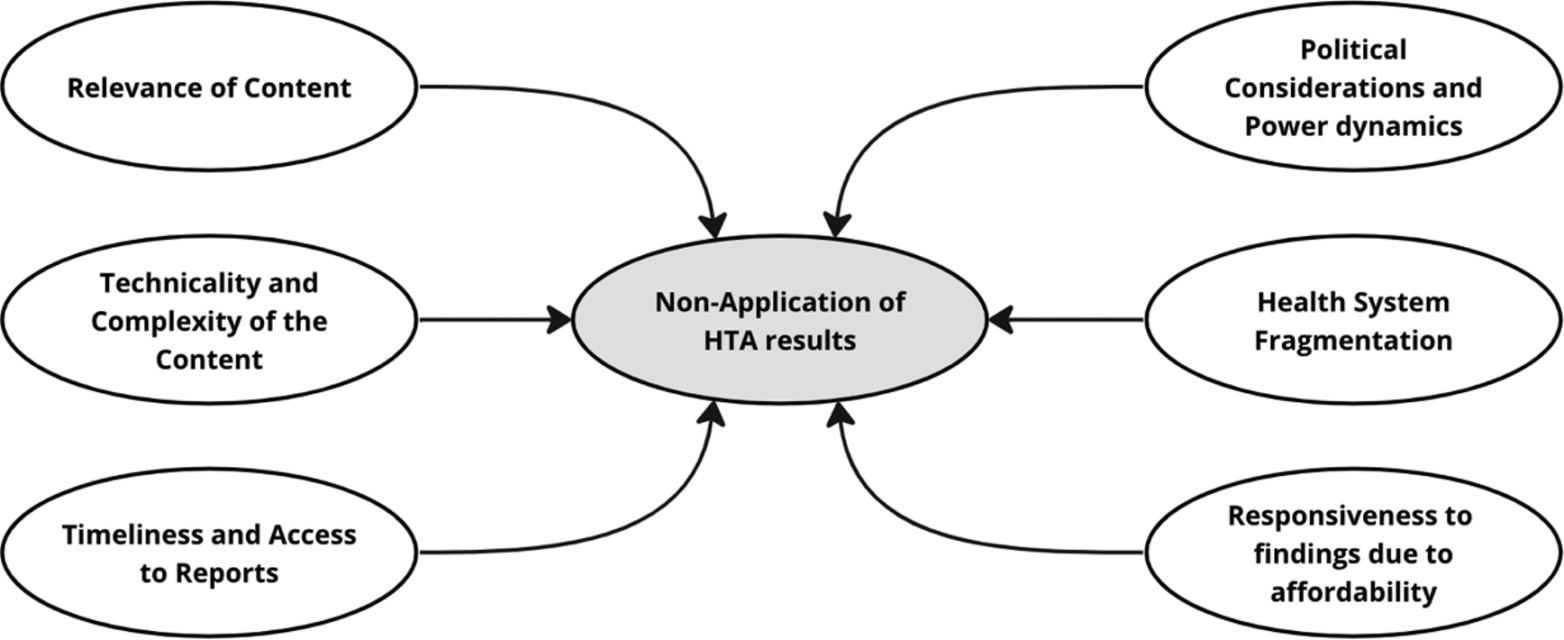
Thematic map: barriers to application of HTA results.

Figure 1 presents the resulting thematic map.

### Timeliness and access to HTA reports

Study participants highlighted that untimely release and limited accessibility to the HTA report impeded its use in decision-making for COVID-19 vaccine procurement in Ghana, especially under the urgency and rapidly evolving context of a public health emergency. Timely release would offer adequate opportunity for comprehension of the evidence and perusal of the cost analyses and their recommendations before subsequent use. Some quotes from participants are captured below:

*“… So, the timing of the result coming out and the timing of the decision on vaccines is also another issue. As to whether the report came out timely enough for decision-makers to be able to use it in that thing is something we really have to look at critically”. (HHS)*

*“One, the timing, but COVID didn’t give anybody any time to do things at the pace that one would have wanted to. It was like the country was running ahead of the recommendations so before we could say ‘jack’ serious decisions have been taken outside the recommendations”. (HHT)*

A participant added that access to the report was a problem because the report was not widely disseminated. It was added that a report that cannot be accessed cannot be used or implemented.

*“So, if the key decision-makers who are the key stakeholders do not have access to the reports… it cannot be used.” (MST)*

It was revealed that even though the report was submitted to the Information Technology department for dissemination through the MOH website, there was uncertainty about the actual publication. This would affect access to the report for implementation.

*“It’s a good question. I think we shared with the IT department for them to upload to the ministry website but to be frank I think I have to check whether they actually have” (HHS)*

### Technicality and complexity of reports

Participants highlighted that the technical density of HTA reports made it challenging for non-technical stakeholders and policymakers to understand, hindering its adoption and implementation. It was widely reported that while the report (shared through a dissemination workshop) contained rigorous data analysis, the language used was complex, with advanced economic analysis. Some participants noted that they were able to understand the material only due to their experience and familiarity with the report.

*“The third one, it will be the ability for the decision-maker to understand what is in that report”(HHS)*

*“But for me, being able to use it at my level and the factors that enhance it, I think it’s because I understand the material and I know what is in it.” (HHS)*

### Relevance of content of the report to decision-making

Participants highlighted a mismatch between the content of the report and the decisions to be made. The government was interested in evidence to select the most effective vaccine; however, the evidence provided was mainly on the cost of deployment, posing a barrier to the application of findings. Some participants noted other factors beyond cost, which are important in vaccine procurement, such as human resources, usage, and benefits.

*“Secondly the content; what actually the report is speaking to. It’s because if I need evidence to be able to select the vaccines and you give me evidence to be able to deploy the vaccines, you are not answering my questions you see.*

*My opinion is that we may recommend evidence, but the evidence becomes just one of the factors that may be considered in prioritization and may be other more complex issues will also be considered in prioritizing a particular action.” (HHS)*

*“… I even like the report about being able to estimate the human resource that will need to apply to this. So, in terms of what they did, one, the cost is also a condition but for me what supersedes the price is about the usage, the benefit.” (HDP)*

Additionally, some participants indicated that regional and international entities were involved in decision-making. The African Union provided pooled procurement mechanisms for vaccines to countries, which were not covered in the report:

*“There were other things that people don’t even want to look at, which the report also didn’t look at. We were doing what we call pool procurement. So, the African Union was also helping countries to choose based on not too much information at the time COVID came and we didn’t have any background data to back, you know. So, remember, there were other mechanisms and other forces at play that were even beyond the country.” (HHT)*

### Political considerations and power dynamics

Participants mentioned that although studies were conducted and reports were generated by technical officers, final implementation decisions were usually made by politically inclined decision-makers. It was therefore difficult to determine the actual factors informing decisions beyond the recommendations of the report.

*“There were other political factors that were beyond the technical work that we did…in my opinion, politicians needed to prove to the masses that they cared about them so they couldn’t wait for the recommendations.” (HHT)*

*“One, political influence in the sourcing. Two, who will be supplying. Because when it comes to the vaccine, it’s one of the essential commodities. So, the political influence, technical we advise the political that for this particular vaccine these are the manufacturers, these are the source, but they make the approval.” (HDP)*

Participants added that as part of political considerations on evidence-informed decision-making, different interests and power dynamics created more complexities, such as the selective use of HTA results. Side-lining scientific evidence not in line with the interests of influential actors leads to inconsistencies in implementation, complicating evidence-adoption with **negotiations, lobbying, and power struggles** among key actors and stakeholders.

*“Yes! So, I think there could be interest and there could be power dynamics as well. So, when the interest is in a particular course of action than what the report seems to suggest and then there is also power to fuel or implement that interest; then you realize that, that combination of interest and power when is contrary to what evidence is saying will have his way” (HHS)*

*“Of course, there are complexities between actors and interests and all those things are very complex, but I think if I add, it’s the complexity of the actor that was a hindrance” (HHS)*

A participant added that the absence of a legal framework to support the implementation of key health evidence leaves room for the use of power in arbitrary decision-making, irrespective of available evidence.

*“But the absence of any legal enforcement or legal backing for the work to be done, then people will be at liberty to do whatever they want. Politically, they can.” (HDS)*

### Health system fragmentation in decision-making

Participants highlighted that despite a well-structured health system in Ghana, fragmentation in coordination, communication, and resource allocation impeded the dissemination of HTA evidence across various levels during the COVID-19 pandemic. Decision-making by MOH primarily targeted the GHS, neglecting other agencies like teaching hospitals, leading to challenges in onboarding HTA recommendations by other stakeholders. Participants pointed out that the system was **highly compartmentalized**, with different agencies, departments, and sectors working in isolation.

*“We will take a decision, where will the decision come from and who will implement it. The ministry will take a decision and only involve GHS and forget the teaching hospitals. One of the things that I saw during the COVID was that all communications were directed to GHS, and I was asking myself ‘is it only GHS which is treating the COVID patients?’” (HDP)*

*“So that is very important in their buy-in. If you don’t get their buy-in, implementation becomes difficult. If you have given to GHS, then they are sitting there unconcerned…” (HDP)*

**Figure 2.**
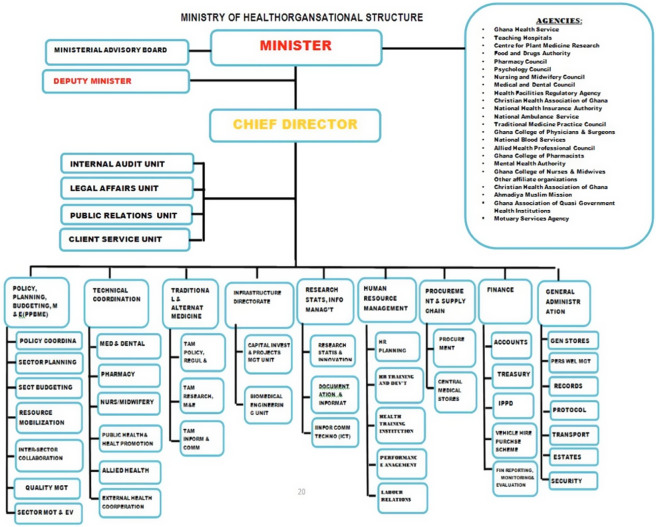
Ministry of Health organizational structure.

To better understand the barriers related to health system fragmentation, a schematic representation of Ghana’s healthcare system is presented in Figure 2 below.

The MOH frames policy and heath sector agenda, while GHS implements healthcare delivery policy with other agencies, including the teaching hospitals, regulatory authorities, and health financing authorities, implementing a specific health-sector agenda. During the COVID-19 pandemic, fragmentation in coordination and communication among these entities led to suboptimal implementation of HTA findings.

### Responsiveness to research findings due to affordability

Participants identified poor responsiveness to research findings due to the affordability of interventions by decision-makers as a barrier to the effective implementation of HTA reports.

*“The recommendations though are efficient recommendations we just couldn’t afford that option … maybe some other factors you see, so recommendations could be good but then it is also possible we are not in the position to just afford it.” (HHS)*

### Overall synthesis of themes

**Figure 3.**
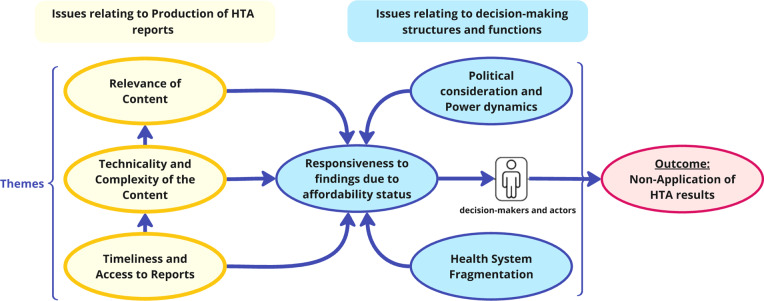
Relationships between identified themes from the thematic analysis.

Figure 3 demonstrates the connections between identified themes. On one hand, challenges relating to the production of HTA reports included being delayed and inaccessible, technically complex with irrelevant content, which are difficult to interpret and apply. These challenges compound limited responsiveness to findings due to affordability constraints. On the other hand, the decision-making environment (structures and functions) is also influenced by political considerations and power dynamics, as different interests and competing priorities often create siloed and fragmented health system operations, hindering responsiveness to evidence. These two broad issues undermine the decision-maker’s application of HTA results and are critical determinants of the implementation of HTA recommendations in the specific context studied.

## Discussion

The uptake of HTA recommendations has been found to vary, influenced by a range of level-specific factors, including socio-political, organizational, and professional dynamics (21;30). These factors refer to phenomena at various strategic and operational levels of health system organization, which affect the uptake of HTA findings. At the socio-political level, the organization and funding of healthcare systems impact the uptake of HTA recommendations. At the level of healthcare institutions, formal organizational structures as well as pre-existing partnerships with HTA bodies facilitate the uptake. For healthcare professionals, the considerable independence of specialists, the significance of peer influence and collective oversight, as well as the clarity of professional obligations, all contribute to the adoption of HTA recommendations into clinical practice (30). Variability in the uptake of HTA findings into national drug reimbursement decisions has also been documented (21). The six barriers identified in this study are not only rooted in socio-political aspects of healthcare organization and funding, but also in HTA process-related issues (see Figure 3).

The first barrier identified by stakeholders – timing of report release – has been identified as an important factor for the uptake of HTA findings by other authors; though in a hospital-level HTA system, with a 2- to 4-month timing projected as ideal (31). Timing was particularly challenging, considering the national rather than hospital-level context in the Ghana case. Ghana developed a national HTA process guideline recommending 37 weeks (9.25 months) to 77 weeks (19.25 months) for a full HTA and 6 weeks (1.5 months) to 18 weeks (4.5 months) for a rapid HTA, respectively, in an attempt to responsively meet evidence needs for policy decision-making (11). Timely access to high-quality, relevant research findings, as well as partnerships, relationship- and skill-building with policymakers, are identified as essentials for evidence use (32;33) with appropriate timing, promoting uptake of recommendations (34). Furthermore, limited access affected effective use, highlighting the importance of timely, accessible communication of HTA results to ensure they can inform decision-making processes. It could be argued that a flexible and iterative decision-making process, open to mid-implementation changes, can adapt to evolving evidence, ensuring responsiveness in rapidly changing situations like a pandemic.

Participants highlighted challenges for non-technical decision-makers and implementers to understand the HTA report (due to technical density), impeding uptake. This emphasizes the breakdown of HTA reports into easily understandable messages for a wide range of stakeholders. This is consistent with studies (35), which identified the complex and technical format of HTA reports to hinder uptake. Inadequate presentation format has been listed among the five most important barriers to the uptake of HTA reports in the Netherlands (36). Capacity building for users of HTA reports can improve understanding and use in decision-making. This includes monitoring and evaluation of the evidence-to-decision process to assess impact.

Participants noted a barrier due to a mismatch between report content and decision-maker needs. This mismatch aligns with the concept of “relevance” in evidence-informed policymaking (32), which promotes research evidence to be aligned with the policy issue at hand. Various factors, including the lack of involvement of decision-makers in the research process, account for this mismatch. Another study (37) highlighted the availability of relevant-HTA research as an essential catalyst in the use of HTA findings, indicating the need to involve decision-makers in the HTA process, align HTA questions with policy needs, among others, as strategies to enhance “relevance.”

Participants noted that the final decisions were largely driven by political considerations through political actors. With the absence of a legal framework on the use of HTA evidence, political actors may express decision-making power considering other factors, potentially neglecting scientific evidence. Non-scientific evidence is reported to play a role in decision-making across various sectors, often overshadowing scientific evidence (38). In public health decision-making, the role of scientific data and distinguishing between reality and methodological assumptions (39) is complex, often hindered by decision-makers’ perceptions and practical constraints (40). The COVID-19 pandemic exposed these limitations of evidence-based decision-making in informing consistent policies across different contexts (41). This was particularly evident in the initial response to health crises, where evidence-based decision-making may not always yield optimal policies (41). In some settings, urgency and precaution were the government’s primary considerations (42). The influence of political considerations and power dynamics on the application of HTA results is consistent with Lukes’ three-dimensional model of power (43), which indicates the exercise of power in three ways: decision-making power, nondecision-making power, and ideological power. This study reveals that political actors wield decision-making and agenda-setting power in vaccine policies, but have little influence over stakeholder perceptions, underscoring the need for stakeholder engagement to effectively bridge the gap between scientific evidence and political decision-making. The MOH has policy processes that promote evidence-based rather than eminence-based approaches (44). These processes are supposed to protect decision-making from overt influences.

Participants noted that although several health and nonhealth agencies were working to contain the pandemic, most decisions taken by the MOH were toward the GHS, neglecting other agencies. Ghana’s health system is well structured with aligned levels of decision-making, yet participants report fragmentations in coordination, communication, and resource allocation under pandemic emergencies, which could have led to distortions in decision-making on HTA recommendations. The reported fragmentation in this study aligns with the health system strengthening framework by the WHO (45). This framework indicates that a well-functioning health system hinges on robust service delivery, an efficient health workforce, a responsive health information system, access to essential medical technologies, sound financing, and effective leadership. The reported fragmentation, particularly in service delivery, workforce, and governance, underscores the necessity for an integrated approach involving all stakeholders in decision-making despite the effect of public health emergencies to promote the use of HTA findings.

Participants noted that research findings not adequately acknowledged, acted upon, or integrated into decision-making can lead to negative outcomes. The study discovered that decision-makers’ inadequate response to the dissemination of the HTA report could be potentially due to the urgency of decision-making, a finding consistent with the concept of “research utilization” in health policy (46). Effective communication and integration of research findings into decision-making processes are often hindered by research complexity, lack of relevance to decision-making needs, decision-makers’ limited capacity to understand and utilize research, and the perceived unaffordability of recommendations.

The barriers identified are similar to those reported by other authors in pandemic evidence-informed decision-making, including rapidly evolving evidence, concerns about scientific integrity, limited capacity for evidence assessment, the need for quick decision-making under uncertainty, and a lack of transparency in decision application (47). In Europe, some barriers to HTA usage included limited generalizability, non-availability of relevant HTA research, lack of consensus between HTA findings, insufficient quality of findings, and lack of access to relevant HTA research (37). In other Sub-Saharan Africa countries, very little use of HTA evidence has been reported due to poor coordination and disconnect from policy (48). In South Africa, a study on challenges to implementing HTA reported: limited political support, local capacity, awareness of HTA, and poor data as barriers (49). The results in all these studies resonate with the findings of this current study. Effective policy reform requires both an understanding of political dynamics and the use of critical technical analysis, as neglecting either can lead to ineffective application of research by technical actors or unattained policy objectives by political actors (50). The effective application of HTA in this public health emergency in Ghana faced multiple barriers, and addressing these through strategies like continuous stakeholder and political engagement, capacity building, health system integration, and adaptive decision-making processes is key to translating HTA results into policy and practice.

## Conclusion

The findings of this study provide an understanding of the barriers to the application of HTA results in Ghana, particularly in the context of the COVID-19 pandemic. Key barriers to the uptake of HTA findings included: inappropriate timing and lack of access to HTA reports; technicality and complexity of content; relevance of content; political considerations and power dynamics; health system fragmentation; and responsiveness to findings due to affordability. Continuous and improved stakeholder engagement (between HTA producers and policymakers) along the HTA production process has the potential to improve uptake of findings, even during public health emergencies.

Supplementary Table S2 highlights the key findings and recommendations (Supplementary Material 8).

## Supporting information

10.1017/S0266462325100342.sm001 Asare et al. supplementary material Asare et al. supplementary material

## Data Availability

All data generated or analyzed during this study will be included in the published article. Where the sources are articles or documents, these would be cited, and references provided to the source articles.

## Supplementary material

The supplementary material for this article can be found at http://doi.org/10.1017/S0266462325100342.

## Limitations

Due to the sensitivity of the subject matter, there were several constraints in securing interviews with all key informants identified in the stakeholder analysis.
