# Supplementary material for: Barriers to the application of Health Technology Assessment (HTA) results: the case of COVID-19 vaccine deployment in Ghana
Source: Int J Technol Assess Health Care. 2026 Feb 2;42(1):e17. doi: 10.1017/S0266462325100342 (PMC12951341; doi:10.1017/S0266462325100342)
Supplement: Asare et al. supplementary material [file S0266462325100342sup001.zip › Supplementary files clean 10.12.25.docx]

**Characteristics of published mini and rapid Health Technology Assessments (HTA) reports: a cross-sectional analysis**

Sharon McLaughlin, MSc, (ORCID: [0009-0004-0787-3434](https://orcid.org/0009-0004-0787-3434))^1^, Aanisa Abeer^1^, Melissa K. Sharp, PhD, (ORCID: [0000-0001-5261-1573](https://orcid.org/0000-0001-5261-1573))^1^, Kieran A. Walsh, PhD, (ORCID: 0000-0002-4386-3012)^2,3^, Cassandra Nemzoff, PhD, (ORCID: [0000-0003-2735-0644](https://orcid.org/0000-0003-2735-0644))^4^, Sheilagh Foley, MSc^5^, Ed Clifton, MSc^6^, Michelle Flood, PhD, (ORCID: [0000-0002-8284-1780](https://orcid.org/0000-0002-8284-1780))^7,8^, Susan Spillane, PhD^2^, Patricia Harrington, PhD, (ORCID: [0000-0003-1774-4420](https://orcid.org/0000-0003-1774-4420))^2^, Conor Teljeur, PhD, (ORCID: [0000-0002-4494-1000](https://orcid.org/0000-0002-4494-1000))^2^, Michelle O'Neill, MSc^2^, Susan M. Smith, MD,^9^ Mairin Ryan, PhD, (ORCID: [0000-0002-3145-0878](https://orcid.org/0000-0002-3145-0878))^2,10^, Barbara Clyne, PhD, (ORCID: 0000-0002-1186-9495)^1^

^1^ Department of Public Health & Epidemiology, School of Population Health, RCSI University of Medicine and Health Sciences, 123 St Stephens Green, Dublin, Ireland

^2^ Health Information and Quality Authority, George’s Court, George’s Lane, Dublin 7, Ireland

^3^ School of Pharmacy, University College Cork, Cork, Ireland

^4^ Department of Global Health and Development, London School of Hygiene and Tropical Medicine, London, England, UK

^5^ Public and Patient Representative

^6^ Scottish Health Technologies Group, Delta House, 50 West Nile Street, Glasgow, Scotland, United Kingdom

^7^ School of Pharmacy and Biomolecular Sciences, RCSI University of Medicine and Health Sciences, 123 St Stephens Green, Dublin, Ireland

^8^ PPI Ignite Network, University of Galway, Ireland

^9^ Discipline of Public Health and Primary Care, School of Medicine, Trinity College Dublin, Dublin, Ireland

^10^ Department of Pharmacology and Therapeutics, Trinity College, Dublin, Ireland

**Corresponding Author:**

Ms Sharon McLaughlin

School of Population Health, Beaux Lane House, Lower Mercer Street,

Dublin 2, D02 DH60

**Email:** [sharonmclaughlin@rcsi.com](mailto:sharonmclaughlin@rcsi.com)

**Supplementary material table of contents**

[Supplementary Table 1: Reasons for exclusion on the International HTA Database and supplementary search (n = 254) 4](#_Toc214291362)

[Supplementary Table 2: Trends over time: Report tags (n= 203) 7](#_Toc214291363)

[Supplementary Table 3: Trends over time: Inclusions of HTA domains (n= 203a) 8](#_Toc214291364)

[Supplementary Table 4: Trends over time: Interest-holder engagement (n= 203a) 10](#_Toc214291365)

[Supplementary Table 5: Trends over time: Evidence synthesis for clinical effectiveness (n= 201a) 12](#_Toc214291366)

[Supplementary Table 6: Trends over time: Evidence synthesis for safety (n= 167a) 14](#_Toc214291367)

[Supplementary Table 7: Trends over time: Evidence synthesis for cost-effectiveness reviews (n= 135a) 16](#_Toc214291368)

[Supplementary Table 8: Report tag vs inclusion of HTA domains (n= 203a) 18](#_Toc214291369)

[Supplementary Table 9: Report tag vs interest-holder engagement (n=203 ^a^) 19](#_Toc214291370)

[Supplementary Table 10: Interest-holder engagement activity within reports 20](#_Toc214291371)

[Supplementary Table 11: STROBE Statement—checklist of items that should be included in reports of observational studies 21](#_Toc214291372)

[Supplementary Table 12: Key recommendations implications for reporting rapid HTAs 28](#_Toc214291373)

[Figure 1 The domains of the HTA Core Model(1) 29](#_Toc214291374)

# Supplementary Table 1: Reasons for exclusion on the International HTA Database and supplementary search (n = 254)

| **Reasons for exclusion** | **Number of studies, N** |
| --- | --- |
| Duplicate reports | 4 |
| Titled “systematic reviews” or "overview of SR" or "rapid SR" either in main title or in main report | 38 |
| Reports assessing causality | 11 |
| Not including two or more full examinations of HTA domains | 45 |
| Reports published before 2014 | 8 |
| Not de novo | 16 |
| Unable to access | 15 |
| Not rapid HTAs (advice documents, modelling exercises) | 7 |
| Not rapid HTAs (evidence review for ionising radiation report) | 2 |
| Not rapid HTAs (descriptive analysis, appropriateness criteria, pilot study assessments, condition level reviews) | 4 |
| Ongoing projects | 2 |
| Not rapid HTAs (review of clinical guidelines, mapping reviews, policy briefs | 10 |
| English version unavailable | 3 |
| Covid-19 quick review | 3 |
| Part of a larger HTA project elsewhere | 1 |
| Rolling/ rapid / living collaborative reviews | 30 |
| Horizon scan | 2 |
| Rapid guidance summary (limited to covid-19) | 21 |
| Not rapid HTAs (field assessment) | 1 |
| Not rapid HTAs (clinical evaluations | 1 |
| Not rapid HTAs (rapid response) | 2 |
| Other (RCT- based economic evaluation, not a classic HTA report) | 2 |
| Not rapid HTAs (Investigation of finance initiatives, review of study procedures, review of indications) | 5 |
| Not rapid HTAs (Strategies available to policy makers) | 1 |
| Not a rapid HTA (review of guidance) | 2 |
| **Supplementary search** | |
| Unable to locate rapid or mini documents from agency websites (no response from agency) | 11 |
| Agency confirmed they had no relevant documents | 5 |
| Agency reports already included in International HTA Database search (RedETS) | 1 |
| Agency handbook confirmed they do not conduct de novo HTAs | 1 |

**Abbreviations:** HTA, Health Technology Assessment; NPHET, National Public Health Emergency Team; SR, systematic review; RCT, randomised controlled trial

# Supplementary Table 2: Trends over time: Report tags (n= 203)

| **Report tag**  **N (%)** | **Year** | | | | | | | | | | |
| --- | --- | --- | --- | --- | --- | --- | --- | --- | --- | --- | --- |
|  | **2014** | **2015** | **2016** | **2017** | **2018** | **2019** | **2020** | **2021** | **2022** | **2023** | **2024** |
| Mini-HTA ^a^ | 2 (100) | 1 (12.5) | 1 (50) | 2 (33.3) | 3 (18.8) | 1 (4.6) | 23 (62.2) | 19 (48.7) | 12 (42.9) | 9 (32.1) | 5 (33.3) |
| Rapid review ^a^ | 0 (0) | 7 (87.5) | 1 (50) | 3 (50) | 13 (81.3) | 21 (95.5) | 14 (37.8) | 20 (51.3) | 16 (57.1) | 18 (64.3) | 10 (66.7) |
| Rapid HTA ^b^ | 0 (0) | 0 (0) | 0 (0) | 1 (16.7) | 0 (0) | 0 (0) | 0 (0) | 0 (0) | 0(0) | 1 (3.6) | 0 (0) |

*^a^* Product tags “mini-HTA” and “rapid review” correspond to the International HTA Database classifications. ^b^ The “rapid HTA” tags refer to the supplementary search, in which two agencies referred to their reports as “rapid HTAs” via email contact.

**Abbreviations:** HTA, Health Technology Assessment.

# Supplementary Table 3: Trends over time: Inclusions of HTA domains (n= 203a)

| **HTA domains**  **N (%)** | **Year** | | | | | | | | | | |
| --- | --- | --- | --- | --- | --- | --- | --- | --- | --- | --- | --- |
|  | **2014** | **2015** | **2016** | **2017** | **2018** | **2019** | **2020** | **2021** | **2022** | **2023** | **2024** |
| CUR | 0 (0) | 6 (75) | 1 (50) | 6 (100) | 13 (81.3) | 19 (86.4) | 13 (35.1) | 16 (41) | 15 (53.6) | 9 (32.1) | 6 (40) |
| TEC | 1 (50) | 7 (87.5) | 2 (100) | 6 (100) | 16 (100) | 18 (81.8) | 36 (97.3) | 31 (79.5) | 21 (75) | 16 (57.1) | 6 (40) |
| EFF | 2 (100) | 8 (100) | 2 (100) | 6 (100) | 16 (100) | 22 (100) | 37 (100) | 38 (97.4) | 28 (100) | 27 (96.4) | 15 (100) |
| SAF | 2 (100) | 4 (50) | 2 (100) | 6 (100) | 13 (81.3) | 18 (81.8) | 31 (83.8) | 33 (84.6) | 27 (96.4) | 20 (71.4) | 11 (73.3) |
| ECO: cost review | 1 (50) | 5 (62.5) | 1 (50) | 1 (16.7) | 5 (31.3) | 8 (36.4) | 29 (78.4) | 29 (74.4) | 24 (85.7) | 22 (78.6) | 10 (66.7) |
| ECO: evaluation | 0 (0) | 2 (25) | 0 (0) | 0 (0) | 1 (6.3) | 4 (18.2) | 6 (16.2) | 12 (30.8) | 12 (42.9) | 7 (25) | 3 (20) |
| ECO: BIA | 0 (0) | 2 (25) | 1 (50) | 0 (0) | 2 (12.5) | 3 (13.6) | 3 (8.1) | 4 (10.3) | 3 (10.7) | 2 (7.1) | 2 (13.3) |
| ORG | 0 (0) | 3 (37.5) | 1 (50) | 1 (16.7) | 7 (43.8) | 13 (59.1) | 29 (78.4) | 26 (66.7) | 22 (78.6) | 10 (35.7) | 3 (20) |
| SOC | 0 (0) | 0 (0) | 1 (50) | 3 (50) | 7 (43.8) | 9 (40.9) | 15 (40.5) | 19 (48.7) | 18 (64.3) | 8 (28.6) | 4 (26.7) |
| ETH | 0 (0) | 1 (12.5) | 1 (50) | 2 (33.3) | 4 (25) | 3 (13.6) | 5 (13.5) | 8 (20.5) | 7 (25) | 2 (7.1) | 1 (6.7) |
| LEG | 0 (0) | 1 (12.5) | 1 (50) | 2 (33.3) | 3 (18.8) | 2 (9.1) | 2 (5.4) | 6 (15.4) | 5 (17.9) | 2 (7.1) | 1 (6.7) |

*^a^* The table does not display reports that had unclear or partial examinations of HTA domains.

**Abbreviations:** BIA, budget impact analysis; CUR, the health problem and current use of technology; ECO, costs and economic evaluation; EFF, clinical effectiveness; ETH, ethical analysis; HTA, Health Technology Assessment; LEG, legal aspect; ORG, organizational aspects; SAF, safety; SOC, patient and social aspects; TEC, description and technical characteristics of the technology.

# Supplementary Table 4: Trends over time: Interest-holder engagement (n= 203a)

| **Interest-holder engagement**  **N (%)** | **Year** | | | | | | | | | | |
| --- | --- | --- | --- | --- | --- | --- | --- | --- | --- | --- | --- |
|  | **2014** | **2015** | **2016** | **2017** | **2018** | **2019** | **2020** | **2021** | **2022** | **2023** | **2024** |
| Advisory group/ expert opinion | 1 (50) | 7 (87.5) | 2 (100) | 3 (50) | 14 (87.5) | 22 (100) | 15 (40.5) | 19 (48.7) | 16 (57.1) | 14 (50) | 7 (46.7) |
| Public consultation | 0 (0) | 7 (87.5) | 1 (50) | 3 (50) | 5 (31.3) | 10 (45.5) | 29 (78.4) | 31 (79.5) | 27 (96.4) | 14 (50) | 11 (73.3) |
| Focus groups | 0 (0) | 0 (0) | 1 (50) | 0 (0) | 1 (6.3) | 0 (0) | 0 (0) | 0 (0) | 0 (0) | 0 (0) | 0 (0) |
| Interviews | 0 (0) | 1 (12.5) | 0 (0) | 0 (0) | 0 (0) | 4 (18.2) | 1 (2.7) | 0 (0) | 2 (7.1) | 1 (3.6) | 1 (6.7) |
| Patient group submission | 0 (0) | 0 (0) | 0 (0) | 0 (0) | 1 (6.3) | 1 (4.6) | 1 (2.7) | 3 (7.7) | 3 (10.7) | 0 (0) | 1 (6.7) |
| Manufacturer contact/ submission | 0 (0) | 1 (12.5) | 1 (50) | 3 (50) | 10 (62.5) | 11 (50) | 4 (10.8) | 2 (5.1) | 3 (10.7) | 2 (7.1) | 2 (13.3) |

*^a^* The table does not display reports that were “unclear” in their inclusion of interest-holders

# Supplementary Table 5: Trends over time: Evidence synthesis for clinical effectiveness (n= 201a)

| **Evidence synthesis: Clinical effectiveness N (%)** | **Year** | | | | | | | | | | |
| --- | --- | --- | --- | --- | --- | --- | --- | --- | --- | --- | --- |
|  | **2014** | **2015** | **2016** | **2017** | **2018** | **2019** | **2020** | **2021** | **2022** | **2023** | **2024** |
| Generic literature search | 0 (0) | 3 (37.5) | 1 (50) | 1 (16.7) | 6 (37.5) | 7 (31.8) | 12 (32.4) | 14 (36.8) | 8 (28.6) | 15 (55.6) | 9 (60) |
| Systematic review | 2 (100) | 2 (25) | 1 (50) | 3 (50) | 5 (31.3) | 6 (27.3) | 4 (10.8) | 6 (15.8) | 5 (17.9) | 4 (14.8) | 4 (26.7) |
| Rapid review | 0 (0) | 0 (0) | 0 (0) | 0 (0) | 0 (0) | 3 (13.6) | 3 (8.1) | 2 (5.3) | 6 (21.4) | 6 (22.2) | 2 (13.3) |
| Overview of systematic review | 0 (0) | 2 (25) | 0 (0) | 0 (0) | 0 (0) | 0 (0) | 0 (0) | 2 (5.3) | 0 (0) | 0 (0) | 0 (0) |
| Update of existing systematic review | 0 (0) | 1 (12.5) | 0 (0) | 0 (0) | 1 (6.3) | 0 (0) | 0 (0) | 0 (0) | 0 (0) | 1 (3.7) | 0 (0) |
| Alternative methods ^b^ | 0 (0) | 0 (0) | 0 (0) | 0 (0) | 1 (6.3) | 1 (4.6) | 0 (0) | 0 (0) | 0 (0) | 0 (0) | 0 (0) |
| Other ^c^ | 0 (0) | 0 (0) | 0 (0) | 1 (16.7) | 3 (18.8) | 5 (22.7) | 8 (21.6) | 6 (15.8) | 5 (17.9) | 0 (0) | 0 (0) |
| Not described ^d^ | 0 (0) | 0 (0) | 0 (0) | 1 (16.7) | 0 (0) | 0 (0) | 10 (27) | 8 (21.1) | 4 (14.3) | 1 (3.7) | 0 (0) |

*^a^* The table does not display reports that had unclear examinations of domains. ^b^ “Alternative methods” refers to reports that conducted multiple types of evidence synthesis for domains; such as a scoping review and a systematic review. ^c^ “Other” refers to reports that had discrepancies in how the reviews were described. ^d^ “Not described” refers to reports that conducted some type of evidence synthesis, but did not specify the method used.

# Supplementary Table 6: Trends over time: Evidence synthesis for safety (n= 167a)

| **Evidence synthesis: Safety**  **N (%)** | **Year** | | | | | | | | | | |
| --- | --- | --- | --- | --- | --- | --- | --- | --- | --- | --- | --- |
|  | **2014** | **2015** | **2016** | **2017** | **2018** | **2019** | **2020** | **2021** | **2022** | **2023** | **2024** |
| Generic literature search | 0 (0) | 1 (25) | 1 (50) | 1 (16.7) | 5 (38.5) | 6 (33.3) | 11 (35.5) | 14 (42.4) | 8 (29.6) | 12 (60) | 7 (63.6) |
| Systematic review | 2 (100) | 2 (50) | 1 (50) | 3 (50) | 4 (30.8) | 6 (33.3) | 4 (12.9) | 5 (15.2) | 5 (18.5) | 3 (15) | 3 (27.3) |
| Rapid review | 0 (0) | 0 (0) | 0 (0) | 0 (0) | 0 (0) | 2 (11.1) | 2 (6.5) | 1 (3) | 6 (22.2) | 4 (20) | 1 (9.1) |
| Update of existing systematic review | 0 (0) | 1 (25) | 0 (0) | 0 (0) | 1 (7.7) | 0 (0) | 0 (0) | 0 (0) | 0 (0) | 0 (0) | 0 (0) |
| Alternative methods ^b^ | 0 (0) | 0 (0) | 0 (0) | 0 (0) | 0 (0) | 1 (5.6) | 0 (0) | 0 (0) | 0 (0) | 0 (0) | 0 (0) |
| Other ^c^ | 0 (0) | 0 (0) | 0 (0) | 1 (16.7) | 3 (23.1) | 3 (16.7) | 5 (16.1) | 5 (15.2) | 4 (14.8) | 0 (0) | 0 (0) |
| Not described ^d^ | 0 (0) | 0 (0) | 0 (0) | 1 (16.7) | 0 (0) | 0 (0) | 9 (29) | 8 (24.2) | 4 (14.8) | 1 (5) | 0 (0) |

*^a^* The table does not display reports that had unclear examinations of domains. ^b^ “Alternative methods” refers to reports that conducted multiple types of evidence synthesis for domains; such as a scoping review and a systematic review. ^c^ “Other” refers to reports that had discrepancies in how the reviews were described. ^d^ “Not described” refers to reports that conducted some type of evidence synthesis, but did not specify the method used.

# Supplementary Table 7: Trends over time: Evidence synthesis for cost-effectiveness reviews (n= 135a)

| **Evidence synthesis: Cost-effectiveness review**  **N (%)** | **Year** | | | | | | | | | | |
| --- | --- | --- | --- | --- | --- | --- | --- | --- | --- | --- | --- |
|  | **2014** | **2015** | **2016** | **2017** | **2018** | **2019** | **2020** | **2021** | **2022** | **2023** | **2024** |
| Generic literature search | 0 (0) | 2 (40) | 0 (0) | 0 (0) | 1 (20) | 1 (12.5) | 10 (34.5) | 11 (37.9) | 6 (25) | 11 (50) | 5 (50) |
| Systematic review | 1 (100) | 3 (60) | 1 (100) | 0 (0) | 2 (40) | 0 (0) | 3 (10.3) | 4 (13.8) | 3 (12.5) | 4 (18.2) | 3 (30) |
| Rapid review | 0 (0) | 0 (0) | 0 (0) | 0 (0) | 0 (0) | 2 (25) | 2 (6.9) | 2 (6.9) | 6 (25) | 6 (27.3) | 2 (20) |
| Other ^b^ | 0 (0) | 0 (0) | 0 (0) | 0 (0) | 2 (40) | 5 (62.5) | 5 (17.2) | 5 (17.2) | 5 (20.8) | 0 (0) | 0 (0) |
| Not described ^c^ | 0 (0) | 0 (0) | 0 (0) | 1 (100) | 0 (0) | 0 (0) | 9 (31) | 7 (24.1) | 4 (16.7) | 1 (4.6) | 0 (0) |

*^a^* The table does not display reports that had unclear examinations of domains. ^b^ “Other” refers to reports that had discrepancies in how the reviews were described. ^c^ “Not described” refers to reports that conducted some type of evidence synthesis, but did not specify the method used.

# Supplementary Table 8: Report tag vs inclusion of HTA domains (n= 203a)

| **Report tag**  **N (%)** | **HTA domains** | | | | | | | | | | |
| --- | --- | --- | --- | --- | --- | --- | --- | --- | --- | --- | --- |
|  | **CUR** | **TEC** | **EFF** | **SAF** | **ECO: cost review** | **ECO: evaluation** | **ECO: BIA** | **ORG** | **SOC** | **ETH** | **LEG** |
| Mini-HTA | 17 (21.8) | 67 (85.9) | 76 (97.4) | 70 (89.7) | 61 (78.2) | 10 (12.8) | 4 (5.1) | 51 (65.4) | 28 (35.9) | 19 (24.4) | 14 (18) |
| Rapid review | 85 (69.1) | 91 (74) | 123 (100) | 95 (77.2) | 72 (58.5) | 37 (30.1) | 17 (13.8) | 64 (52) | 54 (43.9) | 14 (11.4) | 10 (8.1) |
| Rapid-HTA | 2 (100) | 2 (100) | 2 (100) | 2 (100) | 2 (100) | 0 (0) | 1 (50) | 0 (0) | 2 (100) | 1 (50) | 1 (50) |

*^a^* The table does not display reports that had unclear or partial examinations of HTA domains.

**Abbreviations:** BIA, budget impact analysis; CUR, the health problem and current use of technology; ECO, costs and economic evaluation; EFF, clinical effectiveness; ETH, ethical analysis; LEG, legal aspect; ORG, organisational aspects; SAF, safety; SOC, patient and social aspects; TEC, description and technical characteristics of the technology.

# Supplementary Table 9: Report tag vs interest-holder engagement (n=203 ^a^)

| **Report tag**  **N (%)** | **Interest-holder engagement** | | | | | |
| --- | --- | --- | --- | --- | --- | --- |
|  | Advisory group/ expert opinion | Public consultation | Focus groups | Interviews | Patient group submission | Manufacturer contact/ submission |
| Mini-HTA | 22 (28.12) | 67 (85.9) | 0 (0) | 2 (2.6) | 0 (0) | 3 (3.9) |
| Rapid review | 97 (78.9) | 71 (57.7) | 2 (1.6) | 7 (5.7) | 10 (8.1) | 35 (28.5) |
| Rapid-HTA | 1 (50) | 0 (0) | 0 (0) | 1 (50) | 0 (0) | 1 (50) |

*^a^* The table does not display reports that had unclear or partial examinations of HTA domains.

Abbreviations: HTA, Health Technology Assessment

# Supplementary Table 10: Interest-holder engagement activity within reports

| **Advisory group / expert opinion, N (%)** | **Public consultation** | **Focus groups** | **Interviews** | **Patient group submission** | **Manufacturer contact / submission** |
| --- | --- | --- | --- | --- | --- |
| Advisory group / expert opinion ^a^ | 76 (63.3) | 2 (1.7) | 10 (8.3) | 9 (7.5) | 37 (30.8) |
| No advisory group / expert opinion ^a^ | 57 (81.4) | 0 (0) | 0 (0) | 1 (1.4) | 2 (2.9) |

*^a^* Refers to continuous engagement throughout the HTA (e.g. establishment of an expert advisory group or ongoing consultation of experts)

# Supplementary Table 11: STROBE Statement—checklist of items that should be included in reports of observational studies

|  | Item No | Recommendation | Respected? | Comments |  |
| --- | --- | --- | --- | --- | --- |
| **Title and abstract** | 1 | (*a*) Indicate the study’s design with a commonly used term in the title or the abstract | Yes | Study design is indicated in the abstract/ introduction/methods |  |
|  |  | (*b*) Provide in the abstract an informative and balanced summary of what was done and what was found | Yes | This information is stated in the study abstract (study objective described, method and results described) |  |
| Introduction | | |  |  |  |
| Background/rationale | 2 | Explain the scientific background and rationale for the investigation being reported | Yes | Rationale and existing literature are stated in the introduction section |  |
| Objectives | 3 | State specific objectives, including any prespecified hypotheses | Yes | Specific objectives are described in the Introduction section |  |
| Methods | | |  |  |  |
| Study design | 4 | Present key elements of study design early in the paper | Yes | Study design is stated in the first subsection of methods. Key elements are all described in the methods. “This study was a cross-sectional analysis of published rapid HTAs on the International HTA Database” |  |
| Setting | 5 | Describe the setting, locations, and relevant dates, including periods of recruitment, exposure, follow-up, and data collection | Mostly | Setting and the dates of data collection are fully described in the methods section. Recruitment, exposure, follow up is not applicable |  |
| Participants | 6 | *Cross-sectional study*—Give the eligibility criteria, and the sources and methods of selection of participants | Yes | Eligibility criteria (inclusion and exclusion) and sources are described in the methods section |  |
|  |  |  |  |  |  |
| Variables | 7 | Clearly define all outcomes, exposures, predictors, potential confounders, and effect modifiers. Give diagnostic criteria, if applicable | Yes | Variables collected are described in the methods |  |
| Data sources/ measurement | 8* | For each variable of interest, give sources of data and details of methods of assessment (measurement). Describe comparability of assessment methods if there is more than one group | Yes | Data collection and measurement was the same for all variables, and is described in the methods section. Data came from the International HTA Database and HTA agency websites, but were pooled and cleaned on Excel |  |
| Bias | 9 | Describe any efforts to address potential sources of bias | Yes | As the International HTA Database is voluntary to upload, we also carried out a supplementary search of HTA agency websites. This is discussed in the methods section |  |
| Study size | 10 | Explain how the study size was arrived at | Yes | The methods section describes the inclusion and exclusion of reports. The sample size is further described in the results section, the flow diagram, and in Table S1, Supplementary file 1 |  |
| Quantitative variables | 11 | Explain how quantitative variables were handled in the analyses. If applicable, describe which groupings were chosen and why |  | Quantitative variables were analysed using descriptive statistics in the analysis, and are described in the results section and presented in tables |  |
| Statistical methods | 12 | (*a*) Describe all statistical methods, including those used to control for confounding |  | These are described in the methods and results section. We analysed descriptive statistics on the collected variables |  |
|  |  | (*b*) Describe any methods used to examine subgroups and interactions | N/A | Not applicable |  |
|  |  | (*c*) Explain how missing data were addressed | N/A | Not applicable |  |
|  |  | (*d*) *Cohort study*—If applicable, explain how loss to follow-up was addressed  *Case-control study*—If applicable, explain how matching of cases and controls was addressed  *Cross-sectional study*—If applicable, describe analytical methods taking account of sampling strategy | N/A | Not applicable |  |
|  |  | (*e*) Describe any sensitivity analyses | N/A | Not applicable |  |

Continued on next page

| Results | | |  |  |
| --- | --- | --- | --- | --- |
| Participants | 13* | (a) Report numbers of individuals at each stage of study—eg numbers potentially eligible, examined for eligibility, confirmed eligible, included in the study, completing follow-up, and analysed | Yes | The number of eligible reports and excluded reports are reported in the results section and the flow diagram |
|  |  | (b) Give reasons for non-participation at each stage | Yes | Reasons for exclusion are reported in Table S1, Supplementary material file 1 |
|  |  | (c) Consider use of a flow diagram | Yes | The flow diagram is depicted in Figure 1 of the manuscript |
| Descriptive data | 14* | (a) Give characteristics of study participants (eg demographic, clinical, social) and information on exposures and potential confounders | Yes | We have described the general characteristics of the included reports in the results section and Table 1 |
|  |  | (b) Indicate number of participants with missing data for each variable of interest | N/A | Not applicable |
|  |  | (c) *Cohort study*—Summarise follow-up time (eg, average and total amount) | N/A | Not applicable |
|  |  | *Cross-sectional study—*Report numbers of outcome events or summary measures | Yes | All numbers are reported in Tables |
| Main results | 16 | (*a*) Give unadjusted estimates and, if applicable, confounder-adjusted estimates and their precision (eg, 95 percent confidence interval). Make clear which confounders were adjusted for and why they were included | Yes | Findings are presented in the results section, confidence intervals are not applicable |
|  |  | (*b*) Report category boundaries when continuous variables were categorized | N/A | Not applicable |
|  |  | (*c*) If relevant, consider translating estimates of relative risk into absolute risk for a meaningful time period | N/A | Not applicable |
| Other analyses | 17 | Report other analyses done—eg analyses of subgroups and interactions, and sensitivity analyses | N/A | Not applicable |
| Discussion | | |  |  |
| Key results | 18 | Summarise key results with reference to study objectives | Yes | Key results are discussed at the start of the discussion section and summarised in the conclusion |
| Limitations | 19 | Discuss limitations of the study, taking into account sources of potential bias or imprecision. Discuss both direction and magnitude of any potential bias | Yes | Description of limitations is discussed under “Strengths and limitations” heading in the discussion |
| Interpretation | 20 | Give a cautious overall interpretation of results considering objectives, limitations, multiplicity of analyses, results from similar studies, and other relevant evidence | Yes | References were added where possible, and discussed. Limitations were taken into account in the discussion |
| Generalisability | 21 | Discuss the generalisability (external validity) of the study results | Yes | The potential for methods to be underrepresented in our sample has been discussed in the discussion section under “Strengths and limitations” |
| Other information | | |  |  |
| Funding | 22 | Give the source of funding and the role of the funders for the present study and, if applicable, for the original study on which the present article is based | Yes | Funding information has been provided on page 3 of the manuscript |

**Abbreviations:** HTA, Health Technology Assessment;.

# Supplementary Table 12: Key recommendations implications for reporting rapid HTAs

| Detailed reporting of the simplifications/ omissions in rapid HTAs should be improved to support transparency and replicability. |
| --- |
| Detailed justifications of the simplifications/ omissions in rapid HTAs should be improved to support transparency and replicability. |
| Clearer and consistent descriptions of evidence synthesis methods and costing methods are needed to support transparency and replicability. |
| Scope for application of rapid reviews and overview of systematic reviews methods within rapid HTA should be explored. |

# Supplementary Figure 1 The domains of the HTA Core Model(1)

References

1. Kristensen FB, Lampe K, Wild C, Cerbo M, Goettsch W, Becla L. The HTA Core Model(®)-10 Years of Developing an International Framework to Share Multidimensional Value Assessment. Value in health : the journal of the International Society for Pharmacoeconomics and Outcomes Research. 2017;20(2):244-50.
