# Supplementary material for: Barriers to the application of Health Technology Assessment (HTA) results: the case of COVID-19 vaccine deployment in Ghana
Source: Int J Technol Assess Health Care. 2026 Feb 2;42(1):e17. doi: 10.1017/S0266462325100342 (PMC12951341; doi:10.1017/S0266462325100342)
Supplement: Asare et al. supplementary material [file S0266462325100342sup001.zip › Supplementary Material 1_CVIC tool.docx]

**COVID-19 Vaccine Introduction and deployment Costing tool (‎‎‎‎CVIC tool)**

Version 2.3

21 April 2022

 | COVID-19: Vaccines


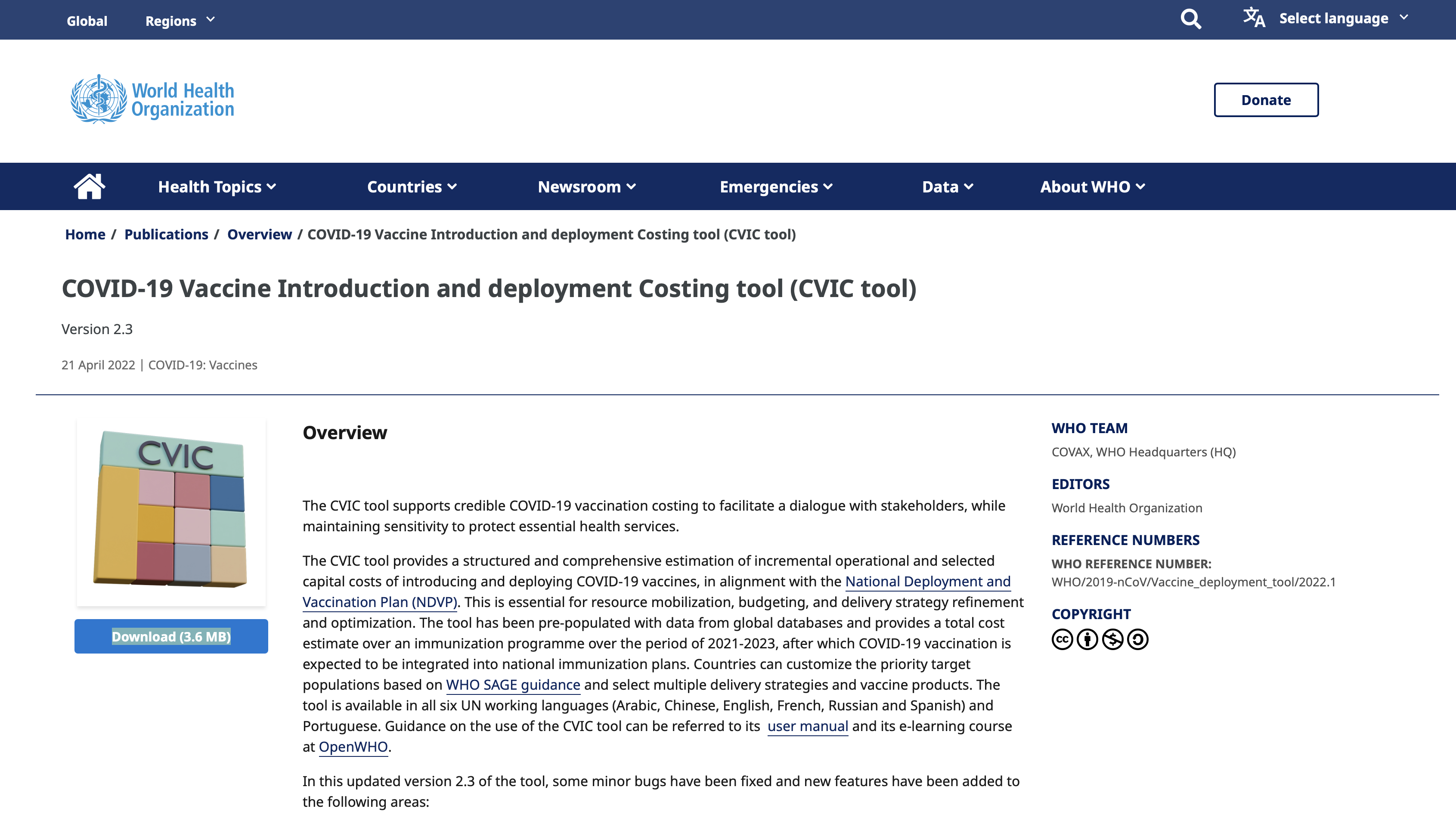


Click to download

<https://iris.who.int/bitstream/handle/10665/353395/WHO-2019-nCoV-Vaccine_deployment_tool-2022.1-eng.xlsx?sequence=1>
