# Supplementary material for: Barriers to the application of Health Technology Assessment (HTA) results: the case of COVID-19 vaccine deployment in Ghana
Source: Int J Technol Assess Health Care. 2026 Feb 2;42(1):e17. doi: 10.1017/S0266462325100342 (PMC12951341; doi:10.1017/S0266462325100342)
Supplement: Asare et al. supplementary material [file S0266462325100342sup001.zip › Supplementary material 2_Report on Costing of COVID-19 vaccinations FINAL 2022.pdf]

Republic of Ghana
Ministry of Health

COSTING OF COVID-19 VACCINATIONS IN GHANA

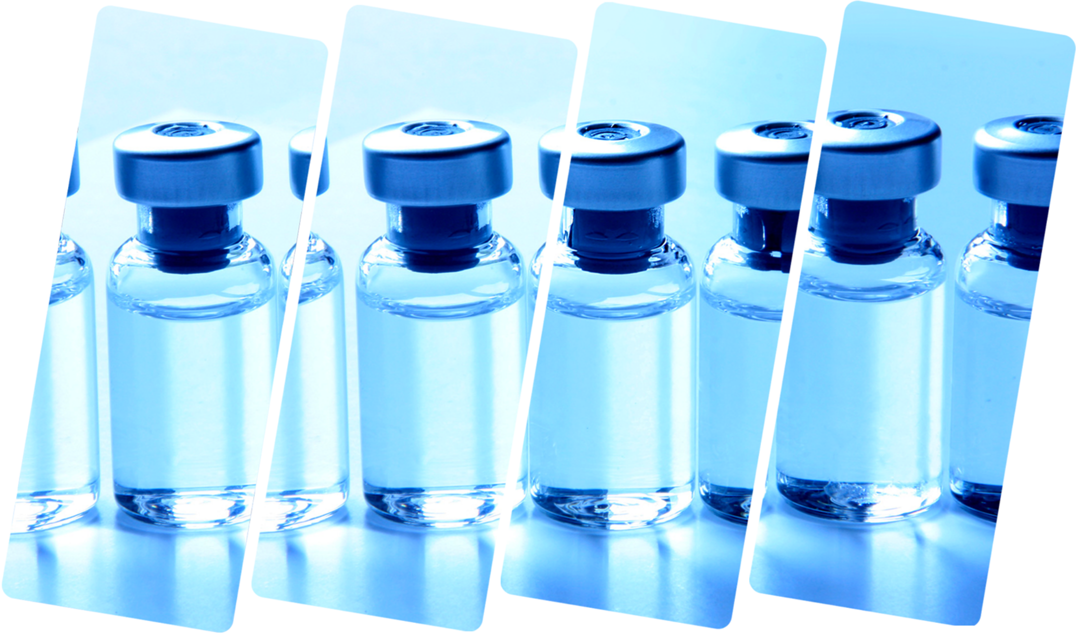

September 2021

A report by the
Ghana Health Technology Assessment
Technical Working Group (**HTA TWG**)

2nd Edition

Cost Analysis of COVID-19 vaccine Introduction and deployment in Ghana

2nd edition

September 2021

A report by the

Ghana Health Technology Assessment

Technical Working Group (**HTA TWG**)

This work was made possible through funding from the World Bank through the Ministry of Health

Content

List of abbreviations 4
List of tables 4
List of figures 4
Acknowledgment 6
Executive Summary 8
1. Background 10
2. Methods 11
Delivery (Deployment) Modalities 11
Scenarios 12
3. Results 14
Targeted Population 14
Grand Total Cost (May 2021 data) 14
Grand Total Cost (September 2021 data) 15
Cost Breakdown by Category (May 2021 data) 15
Cost Breakdown by Category (September 2021 data) 16
Human Resources Requirements for various delivery modalities and Overall Impact of the Vaccination on Health Workforce (May 2021 data) 17
Human Resources Requirements for various delivery modalities and Overall Impact of the Vaccination on Health Workforce (September 2021 data) 18
4. Conclusion and recommendations 19
Conclusion 19
Recommendation 19
Key messages 20
5. References 21
6. Annexes 22
Annex 1 – Technical Workshop on HTA – HTA TWG Analysis Subgroup 22
Annex 2 – [Extract] Report on HTA Steering Committee Meeting on COVID-19 Vaccines 24

List of abbreviations

HTA	Heath Technology Assessment
HTA-SC	Heath Technology Assessment Steering Committee
HTA-TWG	Heath Technology Assessment Technical Working Group
HTA-Sec	Heath Technology Assessment Secretariat
TWG	Technical Working Group
CVIC	COVID-19 Vaccine Introduction and Deployment Costing
UG	University of Ghana
UG-SPH	University of Ghana-School of Public Health
J&J	Johnson and Johnson
EPI	Expanded Programme of Immunization
FTEs	Full Time Equivalents
UNICEF	United National Children Fund
NITAG	National Immunization Technical Advisory Group
NDVP	National Vaccine Deployment Plan
NHIA	National Health Insurance Authority
JSI	John Snow Inc.
PATH	Programme for Appropriate Technology in Health

List of tables

Table 1: Assumptions underlying the various scenarios (May 2021 data) 12
Table 2: Assumptions underlying additional scenarios (September 2021 data) 13
Table 3: Grand Total Cost of COVID-19 Vaccine Introduction and Deployment in Ghana in 2021 – 2022 (May 2021 data) 14
Table 4: Grand Total Cost of COVID-19 Vaccine Introduction and Deployment in Ghana in 2021 – 2022 (September 2021 data) 15
Table 5: Human Resources Implications and Impact (May 2021 data) 18
Table 6: Human Resources Requirements for various delivery modalities and Overall Impact of the Vaccination on Health Workforce (September 2021 data) 18

List of figures

Figure 1: Scenario 1 cost breakdown 16
Figure 2: Scenario 2 cost breakdown 16
Figure 3: Scenario 3 cost breakdown 16
Figure 4: Scenario 4 cost breakdown 16
Figure 5: Scenario 5 cost breakdown 17
Figure 6: Scenario 6 cost breakdown 17
Figure 7: Scenario 7 cost breakdown 17

The scope of HTA encompasses economic evaluation as defined by standard definition.

The National Medicines Policy, 3rd edition 2017, formulated through statutory policy processes, recommends structures to initiate HTA institutionalisation in Ghana.

The policy defines three entities to be tasked with the responsibility for ensuring the successful rollout of HTA in Ghana. Under the section on Health Technology Assessments, the governance structures for implementation of the Ghana HTA strategy 1st edition, 2020, shall be:

1, the **HTA Steering Committee** (responsible for governance),
2, the **HTA Technical Working Group** (responsible for technical functions) as well as
3, the **HTA Secretariat** (responsible for the support and management of all HTA work and processes in Ghana). The HTA Secretariat would work under the Pharmacy Directorate within the MOH structure for Technical Coordination.

While this is the policy position, in order to strengthen the uptake of HTA outputs and enhance transition of HTA recommendations into implementation, the HTA structures and processes would:

1. deliver evidence-based recommendations (as part of its mandate)
2. identify and appropriate policy implementation levers
3. provide recommendations on change management and implementation, informed by implementation research
4. develop impact assessment framework based on explicit criteria
5. assess impact of HTA implementation as part of health systems strengthening

Acknowledgment

The following persons and partners are acknowledged for their support, inputs and efforts in making this analysis possible.

MOH/GHS/other executives

Name	Designation
**Hon. Kwaku Agyeman-Manu (MP)**	Minister for Health
**Hon. Tina Gifty Naa Ayeley Mensah (MP)**	Deputy Minister for Health
**Hon. Mahama Asei Seini**	Deputy Minister for Health
**Mr. Kwabena Boadu Oku-Afari**	Chief Director, MOH
**Dr. (Mrs.) Martha Gyansa-Lutterodt**	Director, Technical Coordination, MOH

National Steering Committee for Health Technology Assessment (HTA-SC)

Name	Designation
**Mr. Kwabena Boadu Oku-Afari**	Chief Director, MOH
**Dr. (Mrs.) Martha Gyansa-Lutterodt**	Director, Technical Coordination, MOH
**Dr. Lydia Dsane-Selby**	CEO, National Health Insurance Authority
**Mrs. Delese Darko**	CEO, Food and Drugs Authority
**Mr. Steve Poku-Kwarteng**	Deputy CEO, Ghana Standards Authority
**Dr. Emmanuel Ankrah Odame**	Director, Policy Planning, Monitoring and Evaluation, MOH
**Dr. Bismark Attah-Adjepong**	Director, Pharmaceutical Services, Ghana Health Service
**Dr. Neema Kimambo**	WHO Representative, WHO Country Office for Ghana
**Mrs. Joycelyn Azeez**	Director, Pharmaceutical Service, MOH
**Mr. Peter Yeboah**	Executive Director, Christian Health Association of Ghana
**Prof. Kwame Ohene Buabeng**	Representative, Academia
**Prof. Francis W. Offei**	Chairman, National Medicine Selection Committee
**Mr. Dan Nuer**	Ministry of Finance
**Mrs. Cecilia Senoo**	Civil Society Organisation
**Dr. Vitus Anaab-Bisi**	Rep. Ghana Association of Quasi Health Institutions

Technical Working Group for Health Technology Assessment (HTA-TWG)

Name	Designation
**Prof. Justice Nonvignon**	School of Public Health, University of Ghana
**Mrs. Joycelyn Azeez**	Pharmacy Directorate, Ministry of Health
**Dr. Augustina Koduah**	School of Pharmacy, University of Ghana
**Dr. Marc Dzradosi**	National Medicines Selection Committee
**Dr. (Mrs.) Angela A. Ackon**	WHO Country Office for Ghana
**Mr. Kwabena Asante Offei**	Private Sector Representative
**Dr. James Akazili**	Research Division, GHS
**Mr. Kwadwo Asante**	Ghana Statistical Service
**Dr. George K. Hedidor**	WHO Country Office for Ghana
**Dr. Kwame Adu Bonsaffoh**	Evidence Summaries Group, NMSC
**Dr. Taiba Afaa Jibril**	Evidence Summaries Group, NMSC
**Dr. Peter Agyei-Baffour**	School of Public Health, KNUST

**Mrs. Ruby Annan**	NHIA
**Dr. Daniel Ankrah**	Rep. Teaching Hospitals
**Mr. Eric Karikari Boateng**	Food and Drugs Authority
**Dr. Maureen Martey**	Policy Planning Monitoring and Evaluation Directorate, MOH
**Dr. Brian Adu Asare**	Pharmacy Directorate, MOH<br>(HTA Country Coordinator)
**Mr. William Omane-Adjekum**	NHIA
**Mr. Saviour Yevutsey**	Pharmacy Directorate, MOH
**Mrs. Elizabeth Adjei-Acquah**	Pharmacy Directorate, MOH
**Dr. (Mrs.) Edith Andrews Annan**	WHO
**Mr. Festus Korang**	Pharmacy Directorate, MOH
**Ms. Edith E. Gavor**	Drug Policy Unit, MOH
**Dr. Richmond Owusu**	Post-doctoral Research Fellow, University of Ghana/MOH
**Mr. Godwin Gulbi**	Health Economist, Korle-Bu Teaching Hospital
**Mrs. Ivy Amankwah**	MOH
**Dr. Emmanuella Abassah-Konadu**	MOH

Secretariat for Health Technology Assessment (HTA-Sec)

Name	Designation
**Dr. Brian Adu Asare**	Pharmacy Directorate, MOH (Head, HTA Secretariat)
**Mr. William Omane-Adjekum**	NHIA
**Mr. Saviour Yevutsey**	MOH
**Mrs. Ivy Amankwah**	MOH
**Dr. Emmanuella Abassah-Konadu**	MOH

Stakeholders Consulted

Name	Designation
**Mr Kwakye Kontoh**	MOH
**Prof. George Armah**	Noguchi Memorial Institute of Medical Research (NMIMR)
**Dr Kwame Amponsa-Achiano**	GHS
**Dr. John Frederick Dadzie**	EPI
**Dr. George Bonsu**	EPI
**Dr. K.O. Antwi-Agyei**	PATH
**Mr. John Tanko Bawa**	PATH
**Mr. Frederick Osei Sarpong**	WHO
**Representative**	UNICEF
**Representative**	JSI

Executive Summary

In addition to the various measures put in place to control and prevent further spread of COVID-19 in Ghana, the government started implementation of the COVID-19 vaccination in March, 2021. Vaccination of the population is one of the strategic measures the country has adopted in line with global efforts to achieve herd immunity.

This report presents the results of the projected cost of COVID-19 vaccine introduction and deployment in Ghana. Using the COVID-19 Vaccine Introduction and Deployment Costing (CVIC) tool developed by World Health Organization (WHO) and United Nations Children's Fund (UNICEF). The Technical Working Group for Health Technology Assessment (HTA-TWG), Ministry of Health, in collaboration with School of Public Health (SPH), University of Ghana (UG), collected data relevant for populating the CVIC tool through a technical workshop with various stakeholders. The data was validated with the Expanded Programme of Immunization (EPI) subsequently. Given prevailing global vaccine market and distribution, **four** main scenarios were analyzed taking into consideration various combinations of vaccines approved for use in Ghana and timelines. Upon further deliberations with the HTA SC, and Presidential advisors on COVID-19, **three** other scenarios were added, resulting in **seven** scenarios.

The scenarios included AstraZeneca (40%), J & J (30%), Moderna, Pfizer, and Sputnik V at 10% each; with full vaccination by second half of 2021 (Scenario 1). AstraZeneca (30%), J & J (40%), Moderna, Pfizer, and Sputnik V at 10% each with full vaccination by first half of 2022 (Scenario 2). There is equal distribution (20%) among AstraZeneca, J & J, Moderna, Pfizer, and Sputnik V; with full vaccination by second half of 2022.

In total, the cost of COVID-19 vaccination ranges between **$348.7 to $436.1 million** for the target population of 17.5 million (i.e., 57% of the population). These translate into cost per fully vaccinated person of **$20.9 to $26.2** and cost per dose including vaccine of **$10.5 to $13.1**. Again, cost per fully vaccinated person excluding vaccine cost between **$4.5 and $4.6**, thus cost per dose excluding vaccine cost also ranged from **$2.2 to $2.3.** The main cost driver is vaccine doses, including shipping, which accounts for between **78% to 83%** of total cost. This is followed by costs to be incurred on vaccinators, which is **8% to 10%** of total cost. Further, between **8,437 to 10,247** vaccinators (non-FTEs) would be required during this period.

COVID-19 vaccine deployment and introduction is estimated to cost about **61% to 76%** of Ghana's 2021 health sector budget allocation for non-remuneration activities and projects. Efforts are required to mobilize the required resources to vaccinate the population against COVID-19, and these findings provide the estimates to inform resource mobilization efforts by government and other partners.

In conclusion, this result can be adopted by the Ministry of Health to support decision making regarding the introduction and deployment of COVID-19 vaccine in the country. Again, it will be an important guide for financing and sustainability of the COVID-19 vaccination programme.

1. Background

Globally, countries have made several efforts to contain the spread of the COVID-19 pandemic since early 2020. In order to prevent the spread, different measures and interventions have been adopted including wearing of nose masks, social distancing, lockdowns, among others. In order to protect global populations from the spread of the virus; effort has been channeled into development of a vaccine and immunization against the virus.

In Ghana, the first two cases of COVID-19 were recorded on the 12th of March 2020 (Ghana Health Service, 2020). Subsequently, 93,711 cases and 783 deaths have been reported as at 22nd May, 2021 (Ghana Health Service, 2021). As of 1st August, 2021, about 198 million confirmed cases of COVID-19 were reported with more than 4.2 million deaths globally (John Hopkins, 2021). In order to control this trend, the government of Ghana decided to vaccinate the population starting March, 2021 in line with the global strategy to achieve herd immunity (Wouters et al., 2021). The decision to vaccinate is informed by best practice. With respect to control and elimination of many infectious diseases, vaccines are the best tools available (Megiddo et al., 2020).

Immunization as a health system intervention against diseases have huge economic and cost implications for countries, and vaccine and immunization deployment for the prevention of COVID-19 is no exception. As a result of this huge cost implication of COVID-19 vaccine introduction and deployment for countries, it is necessary for countries to effectively appreciate the resources required for successful rollout and sustainability. In view of this, the World Health Organization and UNICEF have introduced a costing tool that aims at guiding countries that seek to introduce COVID-19 vaccines.

The tool is used to estimate the incremental costs for resource mobilization purposes including the World Bank's COVID-19 Fast-Track Loan Facility (WHO, 2021). Countries can submit request of their resource needs to the WHO COVID-19 Partners Platform through uploading a completed CVIC tool (WHO, 2021). Countries can also use the tool to prepare budgets for vaccination beyond 2021 as COVID-19 vaccine is deployed. The tool aligns with the COVID-19 vaccine introduction readiness assessment tool, the guidance on developing a national deployment and vaccination plan, and the WHO SAGE values framework for the allocation and prioritization of COVID-19 vaccination.

2. Methods

The CVIC tool is Microsoft Excel-based, with data pre-populated as much as possible – using available country-specific data from global databases. The tool is by default set up to support a rapid cost estimate in a modular approach. In this exercise the version 2.1 of the CVIC tool was used[1]. In order to estimate COVID-19 vaccination costs, the Ministry of Health's Technical Working Group for Health Technology Assessment (HTA-TWG) organized a two-day workshop that brought together relevant stakeholders. These included representatives from National Immunization Technical Advisory Group (NITAG), MOH, GHS, UNICEF, WHO, Expanded Programme for Immunization (EPI), National Health Insurance Authority (NHIA), University of Ghana-School of Public Health, among others. Individuals from these bodies were purposively selected because they were in a position to provide relevant data to populate the tool. It is also worth emphasizing that, relevant information was retrieved from the National Vaccine Deployment Plan (NDVP) for COVID-19 vaccines. Over the two-day period, the mandatory sections of the tool – *population and delivery, unit costs, central costs,* and *target populatio*n were adequately completed. After the workshop, further work was done for cross-examination and validation of the data with the EPI.

Preliminary results for **four** scenarios analysis were presented to the special session of the Ghana HTA Steering Committee with the Presidential Advisors on Health and COVID-19, and the WHO Country Representative in attendance. Additional inputs were collected and incorporated as necessary including additional **three** scenarios, resulting in **seven** scenarios. The final stage of the exercise was a further validation with the EPI and other partners (WHO, UNICEF, JSI, PATH, etc.) to finalize the analysis where additional logistical needs were updated including ultra-cold chain equipment upgrade.

Data analysis was done using the CVIC tool and the results generated are as presented in **Section 3: Results** below.

Delivery (Deployment) Modalities

In the estimation of the cost of deployment, a combination of immunization service delivery modalities available in Ghana were considered. These included:

1. fixed sites with cold-storage (Modality 1),
2. campaigns at fixed sites with no cold-storage (Modality 2),
3. residential institutions (Modality 3) and
4. Outreach & Mobile sites (Modality 4).

[1] https://www.who.int/publications/i/item/who-2019-ncov-vaccine_deployment_tool-2021.1

The separation of these delivery modalities was necessary because each of them has different cost implications. It is noteworthy that delivery modality 3, defined within the CVIC tool in reference to nursing homes for aged people, is unavailable in Ghana, and therefore was excluded from the analysis.

Scenarios

Table 1: Assumptions underlying the various scenarios (May 2021 data)**Table 1** below, presents the various assumptions underlying each scenario. Four main scenarios were analysed. These were based on two overarching assumptions with respect to the types of vaccines used and the time period over which the target population is expected to be fully vaccinated. It was assumed that given the global situation surrounding the vaccine market, it is possible that the time needed to fully vaccinate the target population may extend into the year 2022.

Table 1: Assumptions underlying the various scenarios (May 2021 data)

Scenario	Vaccine type (%)				2021 (%)		2022 (%)	
	Covidshield	Sputnik V	Coronavac	J & J	H1*	H2*	H1*	H2*
1	50	50	-	-	30	70	-	-
2	70	30	-	-	20	50	30	-
3	40	20	20	20	20	30	30	20
4	50	25	15	10	20	30	30	20
Prices	$4.00	$10.00	$5.00	$8.50				

**H1 and H2 – first half and second half of the year respectively*

J&J – single dose, Covidshield, Sputnik V, Coronavac are double doses.

Table 2 below presents the various assumptions underlying each further scenario. Three main scenarios were analysed. These were also based on two overarching assumptions; the types of vaccines to be used and the time period over which the target population is expected to be fully vaccinated. It was assumed that given the global situation surrounding the vaccine market, it is possible that to fully vaccinate the target population may extend into the year 2022. The determination of the prices of vaccines for the analysis was obtained from the UNICEF's COVID-19 Vaccine Market Dashboard[2]. Given that different countries are buying the vaccines at different prices, the team decided to use the median prices for the various vaccines. The main vaccines considered in this analysis are AstraZeneca, Sputnik V, Moderna, Pfizer, and J & J.

[2] https://www.unicef.org/supply/covid-19-vaccine-market-dashboard

In Scenario 5, the assumption is that, majority of the vaccines will be AstraZeneca (40%), this will be followed by J & J (30%), the remaining 30% will be equally distributed between Moderna, Pfizer, and Sputnik V at 10% each.
In Scenario 6, AstraZeneca will be 30% while J & J will be 40%. Moderna, Pfizer, and Sputnik V will be 10% each.

In Scenario 7, there is equal distribution 20% among AstraZeneca, J & J, Moderna, Pfizer, and Sputnik V.

It is worth emphasizing that by the time of this analysis (September 2021), Ghana had covered only **2.7%** of the target population in the **first half of 2021.**

Table 2: Assumptions underlying additional scenarios (September 2021 data)

Scenario	Vaccine type (%)					2021 (%)		2022 (%)	
	AstraZeneca	Sputnik V	Moderna	Pfizer	J & J	H1*	H2*	H1*	H2*
5	40	10	10	10	30	2.7	97.3	-	-
6	30	10	10	10	40	2.7	67.3	30	-
7	20	20	20	20	20	2.7	47.3	30	20
Prices	$4	$19	$18	$14	$10				

**H1 and H2 – first half and second half of the year respectively*

Snapshot of the WHO CVIC tool

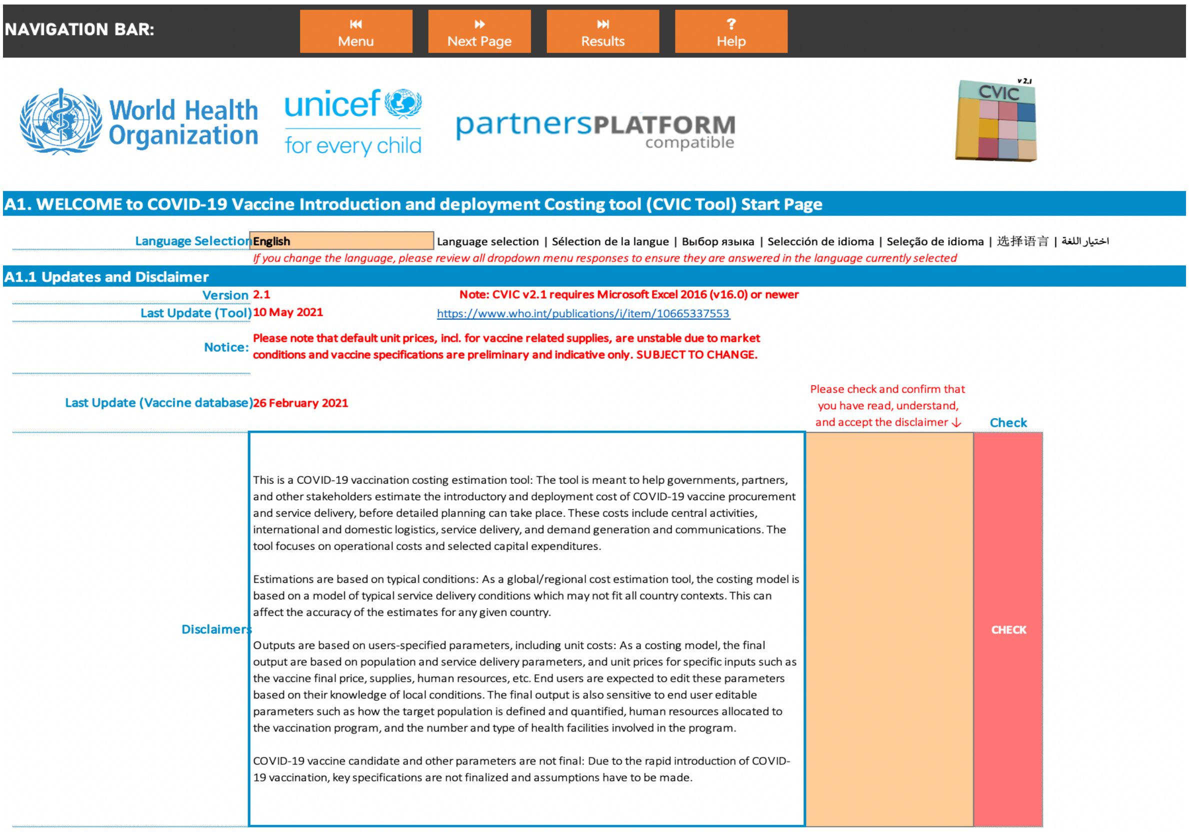

3. Results

This section presents the results from the analysis of data on population and delivery, unit costs, central costs, and target population.

Targeted Population

Ghana plans to vaccinate the total eligible population in the country by the end of the year 2021. However, given the global situation surrounding the vaccine market, it is possible that to fully vaccinate the target population, this may **extend into 2022**. In this, an estimated **17.5 million** of the population are considered to be eligible and will be vaccinated during this period. The projections show that cumulatively **16.7 million** (50.4%) of the total population [(**33 million; 2023 est.**)] will be vaccinated by end of 2022 given the expected uptake rates. Even though, the target is 17.5 million, some people may not volunteer to vaccinate.

Grand Total Cost (May 2021 data)

Table 3 below, shows the grand total cost of the vaccine introduction and deployment in Ghana (May 2021 data). The total cost is estimated to range from **$316.1 million** (Scenario 4) to **392.5 million** (Scenario1). These translate into cost per fully vaccinated person of **$16.7** to **$20.6.** Also, cost per fully vaccinated person excluding vaccine cost between **$3.8** and **$3.9**, thus per dose excluding vaccine also ranged from **$1.90** to **$1.94**.

Table 3: Grand Total Cost of COVID-19 Vaccine Introduction and Deployment in Ghana in 2021 – 2022 (May 2021 data)

					2021		2022		
Scenario	Per fully vaccinated person ($)	Per fully vaccinated person excluding vaccine ($)	Per dose including vaccine ($)	Per dose excluding vaccine ($)	H1	H2	H1	H2	Grand total
1	*20.6*	*3.8*	*10.3*	*1.90*	151.7	240.8	-	-	**392.5**
2	*18.1*	*3.9*	9.05	1.92	72.5	195.8	77.0	-	**345.3**
3	*17.5*	*3.9*	*8.75*	*1.94*	88.5	105.3	90.6	47.5	**331.9**
4	*16.7*	*3.9*	*8.35*	*1.94*	106.4	97.4	65.4	46.9	**316.1**

**H1 and H2 – first half and second half of the year respectively*

Grand Total Cost (September 2021 data)

Table 4 below shows the grand total cost of the vaccine introduction and deployment in Ghana (September 2021 data). The total cost is estimated to range from **$348.7 million** (Scenario 1) to **436.1 million** (Scenario 3). These translate into per fully vaccinated person cost of **$20.9 to $26.2.** Also, per fully vaccinated person excluding vaccine cost between **$4.5 and $4.6**, thus per dose excluding vaccine also ranged from **$2.2 – $2.3.**

Table 4: Grand Total Cost of COVID-19 Vaccine Introduction and Deployment in Ghana in 2021 – 2022 (September 2021 data)

Scenario	Per fully vaccinated person ($)	Per fully vaccinated person excluding vaccine ($)	Per dose including vaccine ($)	Per dose excluding vaccine ($)	2021 H1 ($mil)	2021 H2 ($mil)	2022 H1 ($mil)	2022 H2 ($mil)	Grand total ($mil)
5	*20.9*	*4.5*	*10.5*	*2.2*	33.1	315.6	-	-	**348.7**
6	*21.2*	*4.6*	10.6	2.3	31.4	209.0	112.9	-	**353.3**
7	*26.2*	*4.6*	*13.1*	*2.3*	31.4	238.4	87.0	79.4	**436.1**

H1 and H2 – first half and second half of the year respectively

Cost Breakdown by Category (May 2021 data)

As shown in Figures 1 - 4, the biggest cost driver is vaccine doses, including shipping. This accounts for between **77% (scenario 4)** to **82% (scenario 1)** of total cost. This is followed by the human resources for health; including vaccinator training, deployment, and compensation, which ranged between **7%** to **9%** of total cost.

Other major cost drivers were the Cold Chain and Data Management and Monitoring, Pharmacovigilance, and Oversight. On the other hand, the lowest cost drivers were Domestic Logistics and Transport, excluding cold chain, security, and Standalone Technical Assistance (Figure 1 – 4).

Figure 1: Scenario 1 cost breakdown

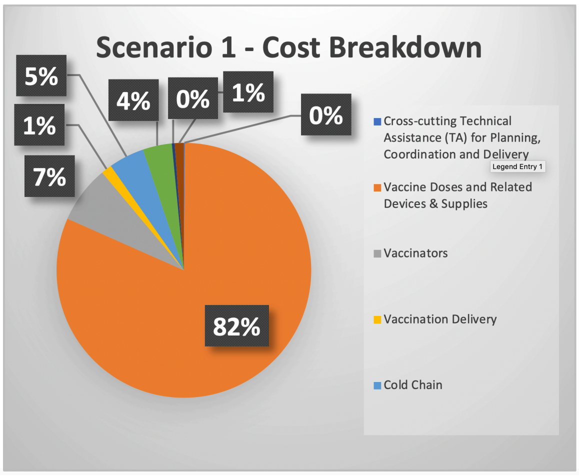

Figure 2: Scenario 2 cost breakdown

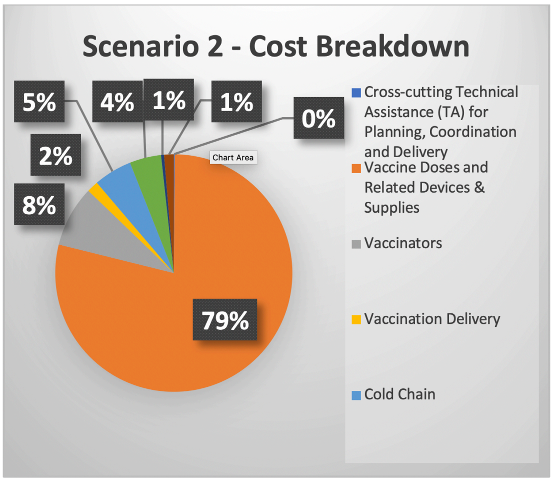

Figure 3: Scenario 3 cost breakdown

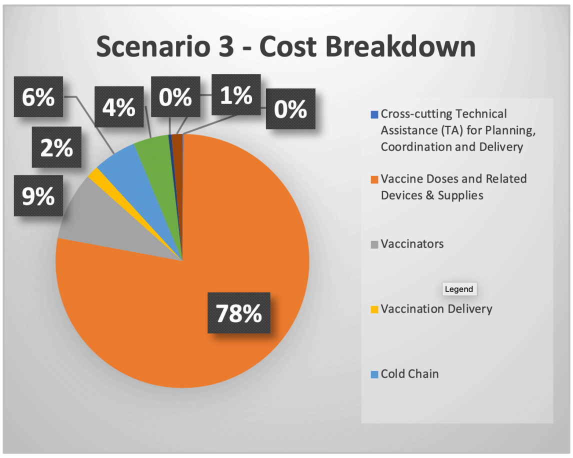

Figure 4: Scenario 4 cost breakdown

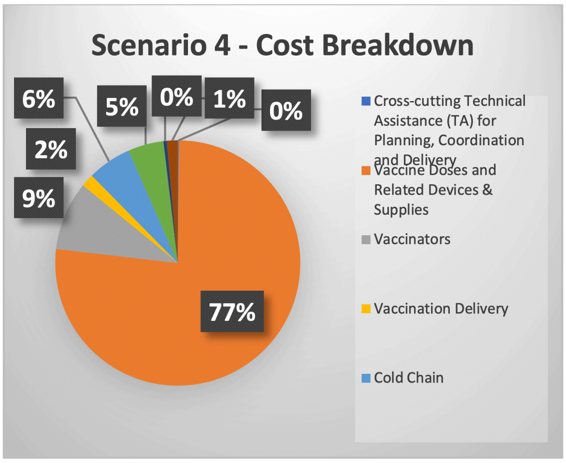

Cost Breakdown by Category (September 2021 data)

As shown in Figures 1 - 3, the biggest cost driver is vaccine doses, including shipping. This accounts for between 78% (scenario 2) to 83% (scenario 3) of total cost. This is followed by the human resources for health including vaccinator training, deployment, and compensation which ranged between 8% - 10% of total cost. Other major cost drivers were the Cold Chain and Data Management and Monitoring, Pharmacovigilance, and Oversight. On the other hand, the least cost drivers were Domestic Logistics and Transport excl. cold chain, security, and Standalone Technical Assistance (Figure 1 – 3).

Figure 5: Scenario 5 cost breakdown

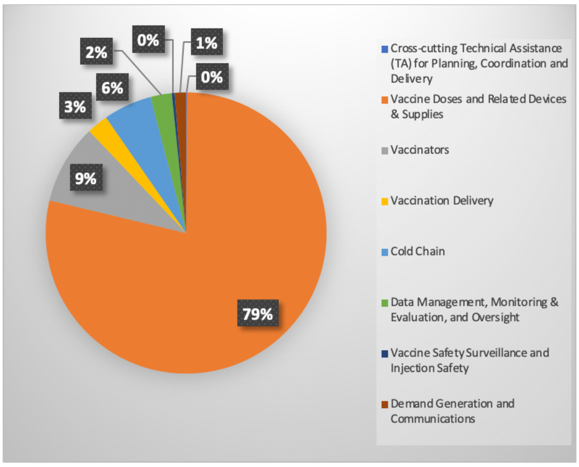

Figure 6: Scenario 6 cost breakdown

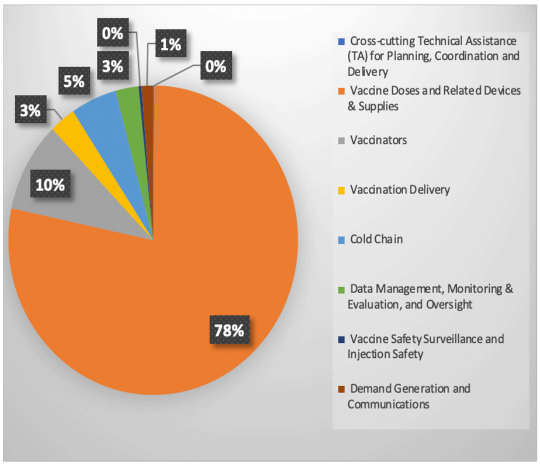

Figure 7: Scenario 7 cost breakdown

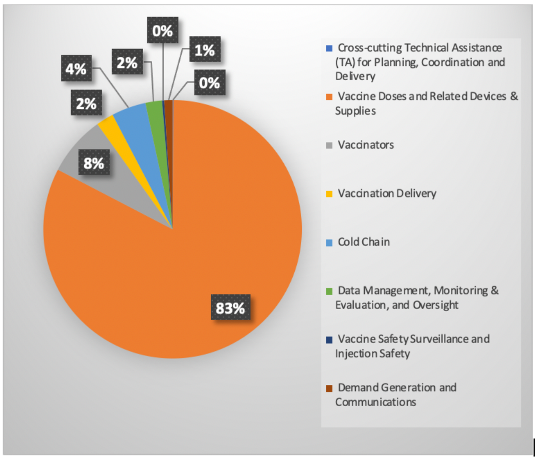

Human Resources Requirements for various delivery modalities and Overall Impact of the Vaccination on Health Workforce (May 2021 data)

Human resources for health are crucial for vaccinations. Regarding the three delivery modalities that Ghana will adopt, majority of the personnel (not FTEs) will be for fixed sites with cold storage (n = 2,554). Here the same number will be required for all scenarios. However, for campaigns at fixed sites with no cold-storage the numbers range from **507** to **1,170.** Also, for outreach and mobile sites the numbers range from **520** to **1188**. In all, a maximum of between **3,589** (scenario 3&4) and **4,912** (scenario 1) of vaccinators will be required (Table 5).

Table 5: Human Resources Implications and Impact (May 2021 data)

Item	Scenario 1	Scenario 2	Scenario 3	Scenario 4
Delivery Modality 1	2,554	2,554	2,554	2,554
Delivery Modality 2	1,170	897	507	507
Delivery Modality 4	1,188	520	528	528
Total Current vaccinators (non-FTEs)	**4,912**	**3,971**	**3,589**	**3,589**
Delivery Modality 1	2,515	1,925	1,100	1,100
Delivery Modality 2	1,143	876	495	495
Delivery Modality 4	914	400	406	406
Total Full-time Equivalents	**4,572**	**3,202**	**1,962**	**1,962**
Total Vaccinator days	**839,406**	**845,222**	**845,314**	**845,314**
HRH supply impact (non-FTEs)	**9.5%**	**7.6%**	**6.9%**	**6.9%**
HRH supply impact (FTEs)	**8.8%**	**6.2%**	**3.8%**	**3.8%**

In addition, a maximum of **1,962** to **4,572** vaccinators (FTEs) will be required, with more than half being in fixed sites with cold-storage (Table 5). Elsewhere, it is estimated that **839,406** to **845,222** vaccinator days will be used during the period under review. This is largely dominated by vaccination at the fixed sites with cold-storage (Table 5). The rollout of this COVID-19 vaccination will impact supply of human resource for health. Table 5 shows that Current Vaccinators (non-FTEs) will impact **6.9%** to **9.5%** of HRH supply and Current Vaccinators (FTEs) will impact HRH supply by **3.8%** to **8.8%.**

Human Resources Requirements for various delivery modalities and Overall Impact of the Vaccination on Health Workforce (September 2021 data)

Human resources for health are crucial for immunization activities. Regarding the three delivery modalities that Ghana will adopt, majority of the personnel (not FTEs) will be for outreach (n = 4,128 - 4,768). For fixed sites with cold storage, the required number of vaccinators (non-FTE) is the same for all three scenarios (n = 2,554). However, for campaigns at fixed sites with no cold-storage the numbers range from 1,755 – 2,925 (Table 3). In all, a maximum of between 8,437 (scenario 7) and 10,247 (scenario 5) of vaccinators (non-FTEs) will be required (Table 6).

Table 6: Human Resources Requirements for various delivery modalities and Overall Impact of the Vaccination on Health Workforce (September 2021 data)

Item	Scenario 5	Scenario 6	Scenario 7
Delivery Modality 1	2,554	2,554	2,554
Delivery Modality 2	2,925	1,833	1,755

Item	Scenario 5	Scenario 6	Scenario 7
Delivery Modality 4	4,768	4,128	4,128
Total Current vaccinators (non-FTEs)	**10,247**	**8,515**	**8,437**
Delivery Modality 1	1,415	943	904
Delivery Modality 2	2,858	1,791	1,715
Delivery Modality 4	2,751	2,382	2,382
Total Full-time Equivalents	**7,023**	**5,115**	**5,000**
Total Vaccinator days	**938,038**	**979,265**	**959,532**
HRH supply impact (non-FTEs)	19.7%	16.4%	16.2%
HRH supply impact (FTEs)	13.5%	9.8%	9.6%

In addition, a maximum of 5,000 to 7,023 vaccinators (FTEs) will be required, with many being in outreach delivery (Table 6). Elsewhere, it is estimated that 938,038 to 979,265 vaccinator days will be used during the period under review; this is largely dominated by vaccination using the outreach modality (Table 6). The rollout of this COVID-19 vaccination will impact human resource for health supply. Table 6 shows that Current Vaccinators (non-FTEs) will impact 16.2% to 19.7% of HRH supply and Current Vaccinators (FTEs) will impact HRH supply by 9.6% to 13.5%.

4. Conclusion and recommendations

Conclusion

In conclusion, this report provides evidence to the MOH to support decision making regarding the introduction and deployment of COVID-19 vaccine in the country. Again, it will be an important guide for financing and sustainability of the COVID-19 vaccination programme.

Recommendation

The cost estimates are important for advocacy and mobilization of financial and other resources for COVID-19 vaccination. Therefore, it is important for government to use these estimates in fundraising discussions with development partners/donors – first, government needs to realistically identify what proportion of the costs or what categories of costs could be borne domestically, as a way to motivate development partner support with the remaining costs.

The estimates generated from the CVIC and reported here are comprehensive and ensures that critical cost elements are not ignored in the quest to raise financial and other resources required for COVID-19 vaccination.

There is the need for consistency in the cost estimates put forward for various discussions, and the results produced in this report are useful for that purpose. To date, varying estimates of costs have been mentioned (in the media, to development partners, etc.), some of which do not include all the categories required for a comprehensive analysis.

Key messages

- Implementation of Vaccination and Deployment of COVID-19 vaccine plans in Ghana will cost **$316** million to **$436** million which translates into cost per fully vaccinated person of **$17** to **$26**
- The estimates show that the biggest cost drivers are
    - vaccine doses, including shipping accounting for **77%** to **83%** of total cost;
    - followed by the human resources for health; including vaccinator training, deployment, and compensation, between **7%** to **10%** of total cost.

The current COVID-19 vaccines supply market means Ghana, like many other LMICs will have to rely on a combination of different sources/types of vaccines to meet their demand for the COVID-19 vaccination programme.

- The CVIC tool is useful for comprehensive COVID-19 vaccine deployment costing and resource planning

5. References

- Ghana Health Service (2021). COVID-19 Ghana's Outbreak Response Management Update Available: https://www.ghanahealthservice.org/covid19/(Accessed, 19th April, 2021)

- Ghana Health Service (2021). Ghana Health Service COVID-19 Situation update Available: https://www.ghanahealthservice.org/covid19/dashboardm.php (Accessed, 19th April, 2021)

- Johns Hopkins University. COVID-19 Map - Johns Hopkins Coronavirus Resource Center [Internet]. 2021 [cited 2021 August 1]. Available from: https://coronavirus.jhu.edu/map.html

- Megiddo, I., Nonvignon, J., Owusu, R., Chalkidou, K., Colson, A., Gad, M., ... & Morton, A. (2020). Fairer financing of vaccines in a world living with COVID-19. *BMJ Global Health*, *5*(7), e002951.

- World Health Organization (2021) COVID-19 Vaccine Introduction and deployment Costing tool, 14 May 2021, version 2.1. Available at: https://www.who.int/publications/i/item/who-2019-ncov-vaccine_deployment_tool-2021.1

- Wouters, O. J., Shadlen, K. C., Salcher-Konrad, M., Pollard, A. J., Larson, H. J., Teerawattananon, Y., & Jit, M. (2021). Challenges in ensuring global access to COVID-19 vaccines: production, affordability, allocation, and deployment. *The Lancet*.

6. Annexes

Annex 1 – Technical Workshop on HTA – HTA TWG Analysis Subgroup

Ministry of Health
HTA Secretariat

1st Technical Workshop on HTA on COVID-19 vaccines in Ghana

Date: 10am on 22nd and 23rd February 2021
Alisa Hotel ridge

Preamble

Ghana is planning the introduction of COVID-19 vaccines for 2021. HTA structures have been established in Ghana, and have a role to play in COVID-19 vaccine deployment. Hence, the HTA structures working closely with key stakeholders including the NITAG, EPI etc. would contribute to the vaccine development and through estimation of the incremental costs for resource mobilization purposes.

The **COVID-19 Vaccine Introduction and deployment Costing** (**CVIC**) tool[3] has been developed to provide a rapid and comprehensive estimation of the incremental costs of introducing and deploying COVID-19 vaccine. This helps in resource mobilization, budgeting, among others.
Ghana may also use the tool to prepare budgets for vaccination beyond 2021 as COVID-19 vaccine is deployed.

Objectives

1. To introduce national stakeholders to the CVIC tool
2. To estimate the costs of the **National Deployment and Vaccination Plan (NDVP**) using the CVIC tool
3. To estimate the incremental costs for resource mobilization purposes
4. To support the preparation of budgets for vaccination beyond 2021 as COVID-19 vaccine is deployed

Expected outputs

- Cost estimates of the NDVP using CVIC tool and Incremental costs on COVID-19 vaccine deployment in Ghana

Schedule

Day 1

3 World Health Organization & United Nations Children's Fund (UNICEF). (2020). COVID-19 vaccine introduction and deployment costing tool (CVIC tool), 7 December 2020, version 1.0 first public release (beta). https://apps.who.int/iris/handle/10665/337553. Licence: CC BY-NC-SA 3.0 IGO

Time	Activity	Lead
10:00am – 10:20am	Opening remarks	MOH Director, Technical Coordinator and TWG Co-Chairs
10:20am – 11:00am	Overview of the National Deployment and Vaccination Plan	EPI Programme Manager
11:00 am -11:20am	Coffee break	
11:30 – 11: 55am	Introduction to the CVIC tool<br>Downloading, and a tour through the tool	Dr. Richmond Owusu
12:00 noon – 1:30pm	Using the Covid-19 vaccine introduction and deployment costing tool (CVIC tool)	Prof. Stephen Resch<br>(Harvard University)
1:30pm – 2:30pm	Lunch break	
2:30pm – 4:00pm	Determination of inputs<br>Population & Delivery	Prof. Justice Nonvignon

Day 2

Time	Activity	Lead
10:00am – 11:00am	Determination of inputs<br>Unit Costs	Mrs. Joycelyn Azeez
11:00 am -11:20am	Coffee break	
11:20am – 12:30pm	Determination of inputs<br>Central Costs	Prof. Justice Nonvignon
12:30pm – 1:30pm	Lunch break	
1:30pm – 2:20pm	Determination of inputs<br>Target population	Mrs. Joycelyn Azeez
2:30pm – 4:00pm	Generation and Discussion of Results	Prof. Justice Nonvignon/ Mrs. Joycelyn Azeez
4:00pm	Closing	

Annex 2 – [Extract] Report on HTA Steering Committee Meeting on COVID-19 Vaccines

2021

Ministry of Health, Ghana HTA Steering Committee
Date: Wednesday 23rd June 2021
Venue: MOH conference room
Time: 1pm

Report of the 3rd Ghana HTA Steering Committee meeting
Meeting on Costing of COVID-19 Vaccines

Preamble

HTA is a relevant tool in decision making in the context of vaccines selection. HTA tools have therefore been used to conduct a scenario-based costing exercise

Objectives

- To present findings from a simulation of various cost scenarios relevant for decision on COVID-19 vaccine selection (informed by the National COVID-19 vaccine deployment plan)
- To take the inputs of the HTA SC on (any) specific parameters of interest to be factored into the scenario-based costing analysis (which has been conducted using the WHO-UNICEF COVID-19 vaccine introduction and deployment costing tool (CVIC tool).
- To contribute data to inform decisions on vaccine selected and procurement

Schedule

	Activity	Lead
1.	Introductions	
2.	Opening Remarks	Hon. Minister of Health
3.	Purpose of the meeting and introduction of the agenda	Dr Mrs Martha Gyansa-Lutterodt,<br>Director Technical Coordination, MOH
4.	HTA technical updates	Mr William Omane-Adjekum, NHIA, HTA Secretariat<br>Dr Brian Adu Asare, Country HTA Coordinator<br>**[8 mins]**
5.	Updates on Cost Analysis of COVID-19 vaccine Introduction and deployment in Ghana	Dr Brian Adu Asare, Country HTA Coordinator,<br>Prof Justice Nonvignon, HTA TWG Co-Chair<br>Mrs Joycelyn Azeez, HTA TWG Co-chair<br>**[15 mins]**

6.	Discussions	HTA SC members
7.	Closing remarks and closing	Hon. Minister of Health

Meeting details

Mode	Physical meeting
Participants	1. Hon. Minister for Health<br>2. Advisors of state/experts on COVID-19<br>3. HTA SC chair and members<br>4. HTA Secretariat<br>5. Coopted HTA TWG members<br>6. MOH/GHS executives
Co-Chairs HTA TWG	Justice Nonvignon, Ghana HTA TWG Chair<br>Joycelyn Azeez, Director Pharmaceutical Services, HTA TWG Co-Chair

Opening

The meeting began at 1pm. The Chairperson, Dr. Mrs Martha Gyansa-Lutterodt, welcomed participants and called on the WHO representative to give an opening remark. Dr. Francis Kasolo, in his opening remark, stated that he is looking forward to the presentation to enable inputs to be made and wished members a successful meeting. The Chairperson introduced the HTA Technical Working Group (TWG) as well as the Co-Chairs of the TWG to all members present.

Priority

1. HTA technical updates
2. Updates on Cost Analysis of COVID-19 vaccine Introduction and deployment in Ghana

Presentations

Presentation 1 - HTA Technical Updates

Dr. Brian Asare gave the HTA Technical Updates which is summarized in the table below:

**Technical Work**	**Update**
Ongoing HTA Analysis	HTA in Diabetes, HTA in Burkitts Lymphoma.<br>Protocols have been written. Pre-situational analysis has been done on Diabetes.
Completed HTA work	Hypertension Model, Costing Analysis of COVID-19 Vaccine Deployment, Cost-effectiveness of Amoxicillin dispersible tablet
Data released by NHIA	Data on top cost drivers has been released by NHIA to help in the optimization the NHIS Medicines List. The purpose is to determine if cost savings can be achieved
HTA Strategy	HTA Strategy completed. HTA Strategy to be launched (sponsored by WHO). Strategy has been costed (cost of institutionalization)
Ongoing (HTA Institutionalization)	Legal assessment to assess whether the current legislation in Ghana is enough to implement HTA in Ghana

Presentation 2 - Cost Analysis of COVID-19 vaccine Introduction and deployment in Ghana

The results from the cost-analysis was presented to the Steering Committee. The presenter mentioned that the COVID-19 Vaccine Introduction and Deployment Costing (CVIC) tool, which was developed by

World Health Organization (WHO) and United Nations Children's Fund (UNICEF), was used in the analysis. The Technical Working Group for Health Technology Assessment (HTA-TWG), Ministry of Health, in collaboration with School of Public Health (SPH), University of Ghana (UG), collected data relevant for populating the CVIC tool through a technical workshop with various stakeholders. The data was validated by the Expanded Programme of Immunization (EPI) subsequently. Given prevailing global vaccine market and distribution, four main scenarios were analyzed taking into consideration various combinations of vaccines approved for use in Ghana and timelines.

From the analysis, in total, the cost of COVID-19 vaccination ranged between **$316.1 and $392.5** million for the target vaccination population of 20 million. These translated into cost per fully vaccinated person of **$16.7 to $20.6** and cost per dose including vaccine of **$8.35 to $10.3**. Again, cost per fully vaccinated person excluding vaccine cost was between **$3.8 and $3.9**, thus cost per dose excluding vaccine cost also ranged from **$1.90 to $1.94.** The main cost driver from the analysis was the vaccine doses, including shipping, which accounted for **77% to 82%** of total cost. This was followed by costs to be incurred on vaccinators, which was **7% to 9%** of total cost. Further, between 3,589 and 4,912 vaccinators (non-FTEs) would be required during this period.
In conclusion, it was iterated that the results of the analysis can be adopted by the Ministry of Health to support decision making regarding the introduction and deployment of COVID-19 vaccine in the country. Again, the findings would serve as an important guide for financing and sustainability of the COVID-19 vaccination programme.

The key messages from the analysis were as follows:

1. Estimates suggest that national vaccination over a 2 year period is relatively more realistic than shorter periods
2. The estimates show that the biggest cost drivers are
    - vaccine doses, including shipping accounting for **77% (scenario 4)** to **82% (scenario 1)** of total cost;
    - followed by the human resources for health; including vaccinator training, deployment, and compensation, between **7%** to **9%** of total cost.
3. Further scenario-based costing is possible in order to present further specific settings of interest to the HTA SC
4. The costing analysis (**for the scenarios considered**) reveals costs from **$316.1 million** (Scenario 4) to **392.5 million** (Scenario1). These translate into cost per fully vaccinated person of **$16.7** to **$20.6**

Discussions

Following the presentations, the following comments were made:

- Discounting was accounted for during the cost analysis after year one.
- Some Efficacy data on the vaccines is available, therefore it would be beneficial to conduct a cost-effectiveness analysis of the various COVID-19 Vaccines. Beyond efficacy, real world effectiveness data could also be used to convert the current analysis to a cost-effectiveness analysis.
- The EPI has used this tool during introduction of other vaccines. It would be ideal to consider the non-vaccine costs that may have been observed in the analysis.

- Price of vaccines is currently being driven by shortage in supply. It should be anticipated that there will be a drop in prices of vaccines next year. The analysis should consider various scenarios where there has been a drop in prices and their possible effect.
- Aside the vaccines that are currently approved by FDA, the analysis should consider looking at vaccines that have not been approved by FDA as they may be cheaper. Also, factor different combinations of vaccines should be considered in the analysis.
- UNICEF prices (global database prices) was used in the analysis. It would be good to factor into the analysis different prices such as the Covax prices and price to the government buying directly from source.
- Cost is extremely relevant when it comes to procurement of the vaccines. The cost element should be developed/ expanded further.
- Stakeholders are essential as they provide different perspectives that may be useful in an analysis. It would be useful if stakeholders are engaged right at the beginning of the analysis rather than at the end in order for inputs to reflect in the analysis right from the beginning
- Assumptions made in the analysis should be clear right from the beginning.
- Cost-effectiveness analysis should be considered when rolling out any health programme
- From a policy perspective, figures from the analysis should be rounded up.
- The Assumption made in the analysis was that existing infrastructure will be leveraged, however cold chain infrastructure that is not available currently could also be factored in the analysis.
- In vaccine deployment, it is cheaper to deploy within a short time rather than a longer period of time. The first Key message from the analysis (*Estimates suggest that national vaccination over a 2 year period is relatively more realistic than shorter periods*) should be explained further. Issues that were considered before concluding that national vaccination over a 2 year period is relatively more realistic than a shorter period should be stated.
- There is a need to state whether economic cost or financial cost was considered for the cost drivers.
- There is a need to look at potential impact of deploying COVID-19 vaccines on routine services (eg. immunization in the children) especially given that the same human resource will be used.
- Key message to focus on is the cost drivers for deployment of the vaccine.
- The TWG should determine the impact of other cost drivers such as training on vaccination, supervision in the analysis.
- Implementation of COVID vaccination has started and is still ongoing. Some information is available at the operational level. The report of the analysis may have to be fine-tuned based on information obtained on the ground.
- Subsequently Pharmacy Directorate should liaise with the EPI and NITAG right from the beginning of such an analysis.

Next Steps

- A policy brief should be written taking into consideration the analysis that has been done and this can be used as advocacy tool
- A final report should be prepared taking into consideration the comments and inputs from the steering Committee
- The HTA-TWG should present findings at NTCC meetings after final report is ready

Closing

The meeting came to a close at 3pm. Dr. Francis Kasolo gave the closing remarks. He congratulated the TWG for the work done and urged the TWG to factor all comments and inputs made into the analysis. Dr. Nsiah Asare urged the TWG to perform cost-effectiveness analysis for various health programmes.

Dr. Mrs Martha Gyansa Lutterodt thanked all members present on behalf of the president and iterated the fact that decision making is not just about priotity setting. She called on Dr. Maureen Martey to give the vote of thanks and Mr. William Agyekum gave the closing remarks.

Appendix

Attendance

NO.	NAME	ORGANIZATION
**1.**	Dr. Mrs. Martha Gyansa-Lutterodt	MOH
**2.**	Mrs. Joycelyn Azeez	MOH
**3.**	Prof. Justice Nonvignon	University of Ghana
**4.**	Dr. Francis Kasolo	WHO
**5.**	Douglas Adu-Fokuo	Presidential Health Advisor's Secretariat
**6.**	Madam Edith Gavor	MOH, DPU
**7.**	Mr. Saviour Yevutsey	MOH
**8.**	Dr. Brian Adu Asare	MOH
**9.**	Prof Kwame Ohene Buabeng	KNUST
**10.**	Christopher Tamal	WHO
**11.**	John Bawa	PATH
**12.**	Ebow Dadzie	EPI
**13.**	Steve Poku-Kwarteng	GSA
**14.**	Asamoah Baah	-
**15.**	Mawunya Akpeke	FDA
**16.**	Karen Boateng	FDA
**17.**	Peter Owusu Asante	MOH
**18.**	Kwasi Abuobi-Yiadom	MOH
**19.**	Dr. Angela Ackon	WHO
**20.**	Dr. Maureen Martey	MOH
**21.**	Fred Osei-Sarpong	WHO
**22.**	George E. Armah	NITAG
**23.**	Mrs. Elizabeth Adjei Acquah	MOH
**24.**	Mrs. Cecilia Senoo	HFFG
**25.**	Georgina Benyah	CHAG
**26.**	Mr. William Omane-Agyekum	NHIA
**27.**	Dr. Richmond Owusu	University of Ghana
**28.**	Godwin Gulbi	KBTH
**29.**	Ivy Amankwah	MOH
**30.**	Dr. Emmanuella Abassah-Konadu	MOH

Apologies

1. Dr. Kwame Amponsa-Achiano (Sent Representative)
2. Prof. Francis W. Ofei – Chairman, NMSC

Copyright © 2021
