# Supplementary material for: Barriers to the application of Health Technology Assessment (HTA) results: the case of COVID-19 vaccine deployment in Ghana
Source: Int J Technol Assess Health Care. 2026 Feb 2;42(1):e17. doi: 10.1017/S0266462325100342 (PMC12951341; doi:10.1017/S0266462325100342)
Supplement: Asare et al. supplementary material [file S0266462325100342sup001.zip › Supplementary Material 3_ Ghana_COVID-19 Vaccine Deployment Plan.pdf]

MINISTRY OF HEALTH
REPUBLIC OF GHANA | A HEALTHY POPULATION FOR NATIONAL DEVELOPMENT

COVID-19 Vaccine Deployment and Vaccination Plan

2021

Acknowledgement

The Ministry of Health and the Ghana Health Service acknowledge the efforts of programmes, departments and divisions for their contribution in the development of this document. The efforts of development partners and individuals in the developing of various sections of the Plan and its harmonization are highly appreciated.

Thank you all.

Executive Summary

Ghana confirmed the first two cases of COVID-19 on 12 March 2020 following which a national emergency was declared by the Government. The Inter-Ministerial Coordinating Committee (IMCC) was constituted as the highest body that coordinates COVID-19 response mechanism in the country. The IMCC is chaired by His Excellency, the President of Ghana and includes representatives from key ministries and the Office of the President. The National Technical Coordinating Committee (NTCC) serves as a technical expert committee in the monitoring of the implementation of activities. The NTCC is chaired by Director General, Ghana Health Service and co-chaired by the WHO.

The national surveillance system was heightened in order to detect cases timely for appropriate management and contact tracing. The Ministry of Health also followed closely the progress on the various vaccine candidates that were being developed.

The National Deployment and Vaccination Plan (NDVP) is the plan of action (POA) developed by the Ministry of Health/Ghana Health Service with the support of health partners to guide health workers on the delivery of COVID-19 vaccines to identified population groups. The Ministry identified vaccination as one of the strategies for the country to combat this pandemic. The decision to access COVID-19 vaccines was based on a rigorous process that involved Ghana's National Immunization Technical Advisory Group (NITAG) and other statutory and regulatory bodies.

The overall target population for the plan is **17,459,408** persons which has been segmented as the deployment will be done in a phased approach. COVID-19 vaccines will be delivered through static, mobile, campout or a combination of these vaccination campaign strategies. Ghana shall deploy **12,471** vaccinators, **37,413** volunteers and **2,079** team supervisors for a planned 2 rounds of vaccination campaigns. Vaccine deployment will be through MOH/GHS decentralized institutions, facilities and structures with oversight responsibility by the country's Expanded Programme on Immunization (EPI).

Support from community volunteers, private providers and Civil Society Organizations (CSOs) will compliment efforts of the GHS for COVID-19 vaccination campaigns. Cascaded training using revised and standardized manuals and guidelines have been planned for all levels of health care. A comprehensive data system that explores synergies among existing data system and new frontiers that include cloud hosting and biometrics are being explored. The objective is to roll out a system that establishes an electronic vaccination register (EVR) in Ghana. Communication and advocacy are essential to the success of the vaccination drive. As a result, strategic plans that deal with risk communication and addresses rumours have been outlined all anchored around generating demand for vaccination.

The current cold chain capacity and volume of COVID-19 vaccines expected requires some minimal expansion of existing cold chain infrastructure. Overall, **15** districts will require new cold chain equipment (CCE) whilst the 7 new regional health directorates need Walk-in Cold

Rooms (WICR). This assumes that the country will deploy vaccines that can be stored at +2 - +8 ^{O}C. Vaccines stored at negative 20 degree Celsius and Ultra cold chain would require completely new installation as contained in this document. About **47** districts will require at least 1 incinerator whilst another **35** districts will require replacement of their non-functional incinerators.

Ghana's Public Health Act 851, 2012 provides the legal basis for vaccine deployment whilst the nations' FDA ensures vaccine safety through existing framework for vaccine registration and regulation in collaboration with partners. An active surveillance system for adverse events following immunization (AEFI) and adverse events of special interest (AESI) will be established to monitor the safety of vaccines and safeguard the health of the people of Ghana. Vaccination teams shall use only Auto Destruct (AD) syringes and shall be provided with safety boxes during the campaign. Additionally, each vaccination point shall carry an emergency kit that includes adrenalin and hydrocortisone.

A comprehensive and integrated monitoring of preparatory, implementation and post campaign activities have been planned. These usually involve deployment of field staff who provide both direct guidance and support to teams at all levels. All levels shall appraise the vaccination campaign locally. The national level in addition will engage institutions to conduct independent assessment of the campaign.

The total amount needed to cover the target population of approximately 17.5m persons is **US$51,662,276 .00**m. The cost of operations per person is about US$3.

The deployment of safe and efficacious vaccines against COVID-19 is expected to protect livelihood, restore economic activities and put the country on course to recover and BRING BACK BETTER.

Table of Content

ACKNOWLEDGEMENT **2**

EXECUTIVE SUMMARY **3**

TABLE OF CONTENT **5**

TABLE OF FIGURES **7**

LIST OF TABLES **7**

ABBREVIATIONS **8**

CHAPTER ONE: INTRODUCTION **10**

1.1 Background and Introduction 10
1.2 General Profile and Demography 10
1.3 Macroeconomics of Ghana 10
1.4 Health Status of Ghana 11
1.5 Burden of COVID-19 12
1.6 Lessons learned from influenza A H1N1 and other relevant activities 13

CHAPTER TWO: PLANNING AND COORDINATION **15**

2.1 Coordination mechanism at country level 15
2.2 Coordination of vaccine deployment and vaccination 15

CHAPTER THREE: REGULATORY PREPAREDNESS AND SAFETY MONITORING **17**

3.1 Background 17
3.2 Objectives 17
3.3 Registration and Importation Pathways 17
3.3.1Emergency Use Authorization (EUA) 17
3.3.2 Requirements for importation and clearance at the port of entry 19
3.3.3 Reliance 19
3.4 Safety Monitoring of COVID-19 Vaccines 20
3.4.1Enhanced spontaneous reporting 20
3.4.2 Active surveillance of AESIs 20
3.4.3 Activities by the MAH 21
3.4.4 Data analysis and causality assessment 21
3.5.5 Training and supportive supervision 21
3.5.6 Communication and information sharing 21
3.5.7 Actors in the surveillance system and their roles 21
3.5 Post-market surveillance of authorized COVID-19 vaccines 22
3.6 Summary 22

CHAPTER FOUR: VACCINATION STRATEGIES **24**

4.1 Overview of strategies 24
4.2 Vaccine choice 24
4.3 Vaccine deployment scenarios 25
4.4 Strategies for vaccine delivery 27
4.5 Integration and continuation of routine immunisation services 28
4.6 Defaulter tracing and reminders 31
4.7 Infection Prevention and Control (IPC) measures 31
4.8 Vaccination data management 31

CHAPTER FIVE: DEPLOYMENT SYSTEMS AND MODALITIES **32**

5.1 Legal basis 32
5.2 Existing Health Infrastructure 32
5.3 Training 32

5.4 Monitoring and Supportive supervision 33

CHAPTER SIX: IMMUNIZATION MONITORING SYSTEMS 35

6.1 DATA NEEDS 35

6.2 Indicators to Monitor Progress 36
6.3 System to record, report, analyze and use vaccination data 36
6.4 COVID-19 vaccination dashboard 37
6.5 Timelines 37

CHAPTER SEVEN: OPERATIONAL RESEARCH 39

7.1 Background 39
7.2 Guidance on priority areas for research 39
7.3 Remobilizing resources to support operational research 40
7.4 Opportunities for dissemination of COVID-19 vaccine related research 40
7.5 Monitor implementation and dissemination of research findings 40
7.6 Timelines 41

CHAPTER EIGHT: COMMUNICATION AND INFORMATION 42

8.1 Introduction 42
8.2 Goal 42
8.3 Expected Outcomes 42
8.4 Objectives 42
8.5 Strategic Approach 43
8.6 Proposed Channels and Measures 44
8.7 Crisis Communication approach 44
8.7.1 Purpose 44
8.7.2 Monitoring and Evaluation 45

CHAPTER NINE: SUPPLY CHAIN PROCESSES 46

9.1 Cold chain capacity 46
9.1.1 National Level 46
9.1.2 Quantifications 47
9.1.3 Regional Level 48
8.1.4 District Level 49
8.1.5 Service delivery points 49
9.2 Dry Storage 50
9.3 Transportation 50
9.4 Vaccine Distribution Pattern 51

CHAPTER TEN: WASTE MANAGEMENT 52

10.1 Policy on Injection Waste Management 52

CHAPTER ELEVEN: EVALUATION AND TERMINATION 53

11.1 Evaluation 53
11.2 Termination of Deployment 53

CHAPTER TWELVE: BUDGET AND FINANCING 55

12.1 Total budget 55
12.2 Financing 55
12.3 Strategy to address the budget gaps 56

1. ANNEXES 57

1-Plan of Action 57

ANNEX II: SUMMARY BUDGET 58

Table of Figures

FIGURE 1:COVID-19 DASHBOARD - GHANA 12
FIGURE 2: COORDINATION OF VACCINE DEPLOYMENT 16
FIGURE 2 *:AEFI FLOW AND CHANNEL* 23
FIGURE 3:SEGMENTATION OF HEALTH FACILITIES (LOWEST DISTRIBUTION POINTS – DISTRICT LEVEL) 49
FIGURE 4: SEGMENTATION OF HEALTH FACILITIES (SERVICE DELIVERY POINTS – SP......... 50
FIGURE 5: VACCINE DISTRIBUTION PATHS 51

List of Tables

TABLE 1:TRENDS IN EPI PERFORMANCE......... 11
TABLE 2: LESSONS LEARNED FROM H1N1 VACCINATION......... 13
TABLE 3:COVID-19 VACCINE PLATFORMS AND CHARACTERISTICS......... 24
TABLE 6: SEGMENTED POPULATION GROUPS AND JUSTIFICATION 25
TABLE 9: VACCINE DEPLOYMENT SCHEDULE (SCENARIO 1)......... 28
TABLE 10: HUMAN RESOURCE NEEDS FOR VACCINE DEPLOYMENT (SCENARIO 1)......... 29
TABLE 11: VACCINE DEPLOYMENT SCHEDULE (SCENARIO 2)......... 29
TABLE 12: HUMAN RESOURCE NEEDS FOR VACCINE DEPLOYMENT (SCENARIO 2)......... 30
TABLE 13: *VACCINE DEPLOYMENT STRATEGIES* 30
TABLE 15: *CADRE OF STAFF FOR TRAINING*......... 33
TABLE 16: TIMELINES FOR DATA MONITORING 37
TABLE 17: TIMELINES FOR RESEARCH 41
TABLE 18: COLD CHAIN EQUIPMENT REQUIREMENTS FOR +2^{0}C TO +8^{0}C AND -20^{0}C 47
TABLE 19: COLD CHAIN EQUIPMENT REQUIREMENTS FOR -70^{0}C 47
TABLE 20: SUMMARY LOGISTICS......... 48
TABLE 21: STATE OF INCINERATORS IN 2020 52

Abbreviations

AD	Auto-destruct
AEFI	Adverse Events Following Immunisation
AESI	Adverse Events of Special Interest
CBO	Community Based Organization
CCEOP	Cold Chain Equipment Optimization Project
CDC	US Centers for Disease Control
CFR	Case Fatality Rate
CHPS	Community-Based Health Planning and Services
COVAX	COVID-19 Vaccine Global Access Facility
COVID-19	2019 Corona Virus Disease
CSO	Civil Society Organizations
CTD	Common technical document
EPI	Expanded Programme on Immunization
EUA	Emergency Use Authorization
EVR	Electronic Vaccination Registry
FAQ	Frequently Asked Question
FDA	Ghana Food and Drugs Authority
GHS	Ghana Health Service
GoG	Government of Ghana
H1N1	Hemagglutinin Type 1 and Neuraminidase Type 1
HCD	Human-Centered Design
IPDC	Infection Prevention, Detection and Control
KAP	Knowledge, Attitude and Practices
MAH	Marketing Authorization Holders
MDA	Ministries, Departments and Agencies
MOFA	Ministry of Food and Agriculture
MOH	Ministry of Health
NADMO	National Disaster Management Organization
NGO	Non-Governmental Organization
NITAG	National Immunization Technical Advise Group
NMIMR	Noguchi Memorial Institute for Medical Research
NTCC	National Technical Coordination Committee
OPV	Oral Polio Vaccine
PASS	Post-Authorization Safety Studies
PHC	Primary Health Care
PHD	Public Health Division
PIE	Post-Introduction Evaluation
PPE	Personal Protective Equipment
QPPV	
QR	Quick Response, a machine readable optical matrix barcode

RMP	Risk Management Plan
SARS	Severe Acute Respiratory Syndrome
TAC-VBP	Technical Advisory Committee on Safety of Vaccines and Biological Products
TOT	Trainer of Trainers
UHC	Universal Health Care
USAID	United States Agency for International Development
VSD	Veterinary Services Directorate
VVM	Vaccine Vial Monitor
WICR	Walk-In Cold Room
WIF	Walk-in Freezer
WB	World Bank
WHO	World Health Organization
YF	Yellow Fever

CHAPTER ONE:
INTRODUCTION

1.1 Background and Introduction

The World Health Organization (WHO) declared COVID-19 a public health emergency of international concern (PHEIC) on 30 January 2020. On 11th March 2020, the WHO declared the novel COVID-19 a pandemic. Ghana confirmed the first two cases on 12 March 2020 following which a national emergency was declared by the Government.

The National Deployment and Vaccination Plan (NDVP) is the plan of action (POA) developed by the Ministry of Health/Ghana Health Service with the support of health partners to guide health workers on the delivery of COVID-19 vaccines to identified population groups.

This plan was developed using the core principles of the WHO Strategic Advisory Group of Experts (SAGE) values framework for the allocation and prioritization of COVID-19 vaccination and the prioritization roadmap. Recommendations by the National Immunization Technical Advisory Group (NITAG) for Ghana informed the final prioritization for the country. This document outlines the various strategies for the deployment, implementation and monitoring of the COVID-19 vaccine deployment.

1.2 General Profile and Demography

Ghana is located in West African and shares borders with Togo, Cote d'Ivoire, and Burkina Faso. According to the 2010 population census, the population of Ghana was 24.2 million and was projected to reach 30,412 million in 2019[1]. According to United Nations estimates, Ghana's population is projected to reach 33.545 million in 2024 at an annual grow rate of 2.5 percent (Ghana Statistical Service). According to the 2010 census, population less than one year is about 3% and one to four-year-old estimated at 10.8 percent (Ghana population and housing census, May 2012). In Ghana, there is no discrimination so far as health service delivery, including immunization, is concerned[2]. However, some equity gaps have been identified among children with different socio-demographic backgrounds. This needs to be addressed.

Ghana has a tropical climate throughout the year with two major seasons – a dry (Harmattan) season and a wet (rainy) season. The dry harmattan season is always severe in the northern part of Ghana, triggering seasonal meningitis outbreaks.

1.3 Macroeconomics of Ghana

According to the 2020 Budget Statement, real gross domestic product grew by 6.2 percent in the first half of 2019 compared to 5.4 in the same period in 2018. The service sector recorded a

[1] UN estimate, World population review, www.worldpopulationreview.com
[2] Ghana PSR, 2020-2024, page 14

significant growth of 6.9 percent as a percent of gross domestic product between January and June 2019 compared to 0.9 percent in the same period in 2018. It is significant to note that the growth in the social sector was predominantly from information and technology (37 percent) and health and social work (22 percent).

Ghana's growth target for 2019 was 7.4% and mainly driven by the industry sector, especially oil, gas and mining. Manufacturing is also expected to post higher growth. In the medium-term (2019-2022), overall Gross Domestic Product (GDP) is projected to grow on average at 7.0%, as the effects of oil on growth decline and non-oil growth strengthens. Inflation is expected to remain in the central bank's target range of 6-10%, while the fiscal deficit is expected to be marginally higher at 4.2% of GDP.

1.4 Health Status of Ghana

The health system of Ghana is organized under four functional areas; health administration and financing, healthcare service delivery, training and regulatory. These functional areas are performed by public, private-not-for profit, private–for-profit and traditional sectors. All services in the health sector are organized at national, regional and districts levels. Though services are performed below the district level, not all functions have organizational structures below the district level.

Healthcare services are categorized under primary, secondary and tertiary levels. Community-Based Health Planning and Services (CHPS) approach is the national strategy in addressing service delivery gaps in access to quality health services at the community level. The Ghana Health Service serves as the supervising agency for the delivery of primary and secondary healthcare services in the country on behalf of the MoH. It is also the largest implementing agency for primary and secondary care services (both preventive and curative care) in addition to non-state actors. CHPS is equity-focused and has an implementation modality that has strong support of the government and development partners in the health sector. The health sector is strongly supported, both financially and technically, by Development Partners (DPs) in all the functional areas.

The organization and governance of the health system in Ghana has set the pace for the current strengths of the country's routine immunization system as well as national immunization campaigns which augment routine efforts.

Table 1:Trends in EPI Performance

Antigen	2015	2016	2017	2018	2019
BCG	95	100	100	98	96
OPV-3	96	93	90	98	99
Pentavalent-3	95	94	98	98	99
PCV-3	96	94	97	98	100
Rotavirus-2	94	91	93	94	95
MR1	94	95	95	95	95
Measles (MR-2)	72	70	78	83	87
Td+	65	63	65	64	66

1.5 Burden of COVID-19

Coronaviruses are a large family of viruses that may cause illness in animals or humans. In humans, several coronaviruses are known to cause respiratory infections with symptoms ranging from the common cold to more severe diseases such as Middle East Respiratory Syndrome (MERS) and SARS. The most recently discovered coronavirus, severe acute respiratory syndrome coronavirus 2 (SARS-CoV-2), causes coronavirus disease 2019 (COVID-19). COVID-19 was unknown prior to the outbreak in Wuhan, China, in December 2019, but is now a pandemic affecting most countries globally.

By December 2020, the total number of COVID-19 cases had risen to 67,939,443 million with 1,550,271 deaths, according World Health Organization (WHO) reports[3]. On the African continent the pandemic has affected 47 African countries with 738,344 cases and 12,476 deaths. A similar situation pertains in Ghana since the country recorded its first cases on 12 March,2020. By December 2020, the country has recorded a total of 37,812 cases and 191 deaths (CFR of 0.51%). Total discharges and recoveries are 34,313 (90.7%) and 3,308 (9.3%) respectively. All 16 regions have reported cases.

The understanding of COVID-19 epidemiology continues to evolve and is rapidly changing. A description of the COVID-19 disease and what is currently understood of its transmission patterns can be found in https://ghanahealthservice.org/covid19/.

Figure 1:COVID-19 Dashboard - Ghana

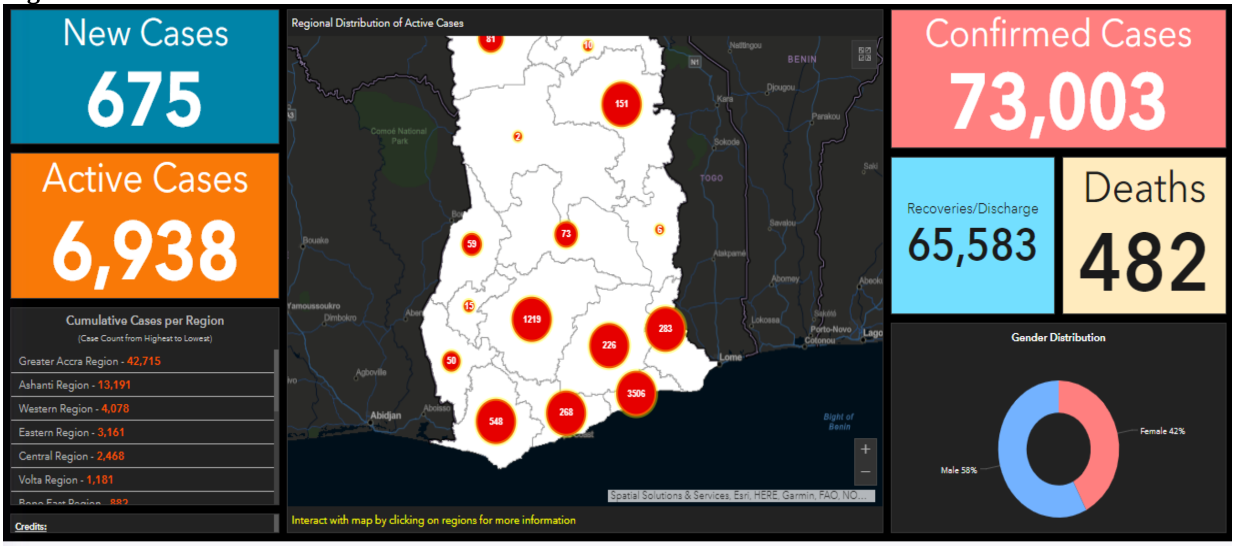

Source: ghanahealthservice.org/covid19 | Date: 09/02/2021 | time: 16:56GMT

[3] WHO Live COVID-19 dashboard assessed at https://covid19.who.int/

1.6 Lessons learned from influenza A H1N1 and other relevant activities

Ghana recorded a total of 789 (12%) cases of Pandemic Influenza A H1N1 2009[4] as of 27 May 2010 with one death. The country deployed Pandemrix Pandemic Influenza Vaccine to priority population groups and learned valuable lessons which are expected to guide future response to pandemics. The lessons learned are tabulated below;

Table 2: Lessons learned from H1N1 Vaccination

Lessons Learned	Solutions / Action Points
**Coordination**<br>• The country reorganized existing administrative and organizational structures and institutions for Severe Acute Respiratory Syndrome (SARS) and Avian Influenza (AI) to form the National Technical Coordination Committee (NTCC) for Pandemic Influenza A H1N1.	• The NTCC has been maintained to oversee all national public health emergencies. Technical Working Group with sub-committees has been constituted to oversee various components of the introduction
**Prioritization/segmentation**<br>• Ghana identified population sub-groups that were at high-risk of the disease and prioritized them for the vaccine	• Ghana plans to vaccinate the entire population against COVID-19. However, population groups have been prioritized and that will inform the vaccine deployment
**Technical guidance**<br>• Decision on the whether to introduce the vaccine, the choice of vaccine, prioritization etc were advised by the technical working group and approved by ICC	• The country currently has a functional National Immunization Technical Advisory Group (NITAG) that advises the Ministry of Health on immunization related issues. The NITAG provided guidance which influence all decisions on COVID-19 vaccines
**Public education and communication**<br>• Communication and public education were minimal and mostly among health staff. The general public were not adequately informed of the vaccination	• The Ghana Health Service will conduct extensive advocacy with all stakeholder and conduct social mobilization activities at all levels to sensitive the public
**Training and reference materials**<br>• Trainings were conducted at all levels. However, no reference materials were	• Trainings will be conducted at all levels. Guidelines (job aids) will be prepared, printed and distributed before to all actors

[4] H1N1 cases in Ghana assessed at https://www.ghanahealthservice.org/downloads/Weekly_Epidemiological_Bulletin_Web_20.pdf

developed as job aids for vaccinators and other actors	
**Data Management**<br><br>• Details of vaccinees were entered into an MS Excel Database. The database became corrupted at some point and data retrieval became very difficult	• Ghana intends to use Oracle Application to build a robust database of all vaccinees that can be easily retrieved. The data will also be protected under the country's data protection act
**Unavailability of emergency drugs in some vaccination points**<br><br>• During the H1N1 Vaccination campaign, there were a considerable number of vaccination sites where emergency drugs were not available. Also, people were not made to sit and wait for about 15-30 minutes to be observed for any untoward event	• Emergency drugs will be procured and distributed widely to all vaccination sites and health facilities. Vaccinators will be trained to ensure all vaccinees wait for at least 15 minutes to enable health workers observe them before they leave
**Rumours**<br><br>• There was a rumour from a lead person of a political party which nearly marred the campaign in one region of the country. There were other rumours which affected patronage of the campaign	• The country is in the process of cataloguing all circulating rumours on the COVID-19 vaccines and will develop a Q&A session with the media as well as documents to get the public informed/sensitized. The communication team will also be proactive in dealing with all rumours during implementation

CHAPTER TWO:
PLANNING AND COORDINATION

2.1 Coordination mechanism at country level

The Inter-Ministerial Coordinating Committee (IMCC) is the highest body that coordinates COVID-19 response mechanism in the country. The IMCC is chaired by His Excellency, the President of Ghana and includes representatives from the Ministries of Finance, Health, Local Government, Gender, Children and Social Protection, Information, Transport, Interior, Defense and the Office of the President.

The National Technical Coordinating Committee (NTCC) serves as a technical expert committee in the monitoring of the implementation of activities. The NTCC is chaired by the Minister of Health, co-chaired by the WHO. The National Public Health Emergency Operations Centre (PHEOC) is the mechanism responsible for coordinating COVID19 response activities and is chaired by the Director-General of the Ghana Health Service and commandeered by the Director, Public Health. It is activated when there is an outbreak and the activation is scaled-up depending on the risks and epidemiological situation of the outbreak. In addition, the National Disaster Management Organization (NADMO) is a government agency responsible for the management of natural disasters and other emergencies (reporting to the Ministry of Interior).

At the regional and district levels, preparedness and response activities are coordinated by the respective regional and district Public Health Emergency Management Committees (PHEMCs). Health partners, including WHO, UNICEF, World Bank, Gavi, DFID, JICA etc, are standing members of the National Technical Coordinating Committee, the PHEOC and the sub-group on Risk Communication and Community Engagement (RCCE).

2.2 Coordination of vaccine deployment and vaccination

Coordination of the vaccine deployment will be within the IMCC and the NTCC governance framework. The National Immunization Technical Advisory Group (NITAG) is responsible for providing independent, evidence-informed advice to policy makers and the EPI Programme on policy issues related to COVID-19 vaccine deployment. The NITAG reports to the Ministry of Health and recommendations from the Group are fed into meetings of the NTCC and the IMCC.

Inter-agency Coordinating Committee (ICC) for Immunization plays an oversight role for the EPI Programme. The committee coordinates technical and material inputs to the programme, increase technical coordination, ensuring efficient use and greater impact of technical, material and financial resources.

With regards to COVID-19 vaccine deployment, the ICC provides technical and managerial support and leads the resource mobilization drive. It participates in the planning, monitoring and evaluation mechanisms of the immunization programme and provide recommendations where necessary.

The Director-General of the Ghana Health Service has established the Technical Working Group (TWG) for COVID-19 Vaccine Readiness and Deployment which is responsible for planning and deploying the COVID-19 vaccines. The TWG has seven sub-committees which are responsible for the following; coordination and resource mobilization, training and service delivery, regulatory and safety, data management, logistics and waste management, communication and research and surveillance

Figure 2: Coordination of Vaccine Deployment

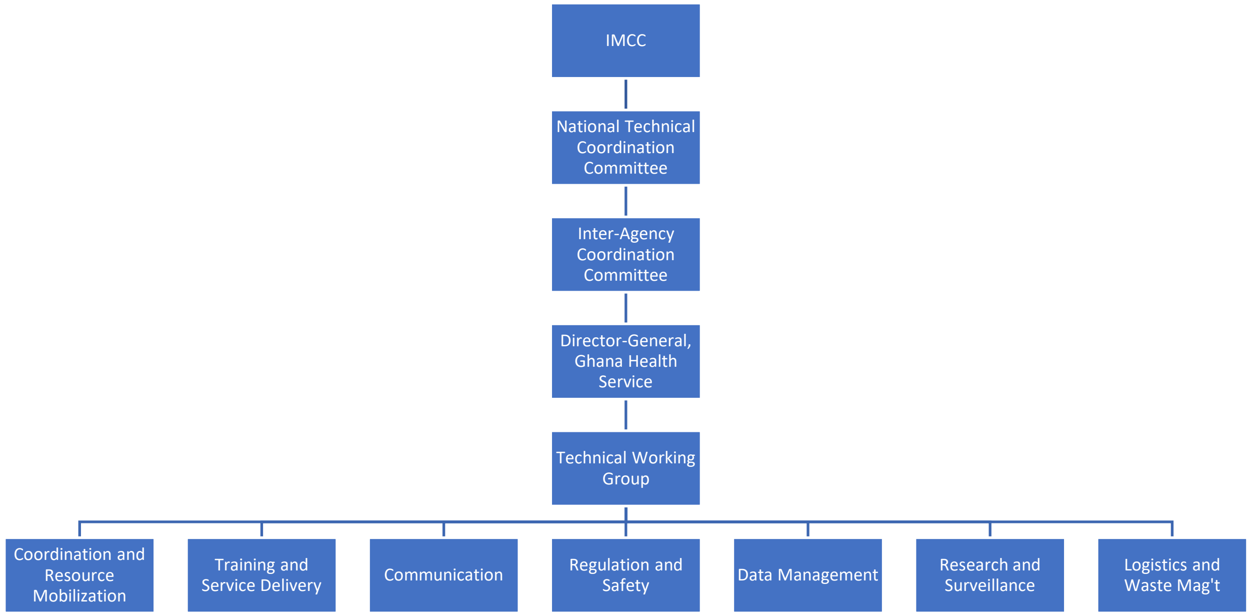

The Expanded Programme on Immunization (EPI) is in-charge of immunizations in Ghana and is responsible for vaccine deployment. It is located within the Diseases Control Department (DCD) of the Public Health Division (PHD) of the Ghana Health Service. The Programme is headed by a Public Health Specialist who reports to the Director for Public Health. He is assisted by trained personnel who are specialists in areas that include logistics management, data management, monitoring and evaluation, cold chain management, injection safety, social mobilization and communication. The EPI Programme coordinate the activities of the sub-committees

CHAPTER THREE:
REGULATORY PREPAREDNESS AND SAFETY MONITORING

3.1 Background

Ghana's Food and Drugs Authority (FDA) is mandated by the Public Health Act 2012, Act 851 to ensure only safe and efficacious vaccines are made available to Ghanaians. This chapter seeks to expedite registration of COVID-19 vaccines to be deployed during the pandemic and to carry out the safety monitoring of these vaccines to identify any new safety information arising from these vaccines.

3.2 Objectives

i. Describe the regulatory pathway and requirements to expedite the registration, importation and clearance of any COVID-19 vaccine
ii. Rapidly detect, investigate and manage any safety issues during the deployment of COVID-19 vaccines
iii. Conduct prompt and effective communication of new safety information arising from the safety monitoring of COVID-19 vaccines and effect corresponding regulatory action when needed

3.3 Registration and Importation Pathways

The underlisted pathways will be employed.

3.3.1Emergency Use Authorization (EUA)

The FDA Ghana has defined regulatory pathways and systems in place to expedite the processing of COVID-19 vaccine applications towards authorization, and to monitor the safety of these vaccines to identify new safety information once they are deployed.

Generally, all COVID-19 vaccine applications shall be expeditiously processed towards Emergency Use Authorization. However, if a COVID-19 vaccine has been authorized for use by a stringent National Regulatory Authority (NRA) (EMA, USFDA, TGA, Health Canada and Japan), and the applicant decides to share the full assessment report with the FDA, the process of evaluation and authorization is significantly expedited. This is because the FDA may decide to rely on the decision of the stringent NRA to arrive at a decision while maintain its regulatory responsibilities and decision making.

In situations where the COVID-19 vaccine has not been authorized for use by any of the stringent NRAs, the application shall be subjected to full evaluation which might delay the processing timeline, albeit, receiving a response from the FDA within the designated timeline of 15 working days.

The FDA shall require EUA applicants to at least submit the underlisted regulatory requirements to enable them make an informed regulatory decision on the Authorization request.

1. **Format for submission**: Product development dossier shall be submitted in an electronic format, (two (2) copies either saved on a USB flash drive or on CDs), together with an application letter addressed to the Chief Executive Officer (CEO) of the Food and Drugs Authority (FDA).
2. The application should be submitted through the authorized local agent by the regulatory contact person to the following address:
3. All documents submitted for the purpose of an EUA shall be in English, and must be legibly printed and not handwritten. The FDA expects material to be provided in a reviewable form and sufficiently complete to permit substantive evaluation
4. Description of the product and its intended use (e.g., identification of the serious or life-threatening disease or condition for which the product may be effective)
5. Description of the product's global/international license /registration/Marketing Authorization (MA) status or whether the medical product is prequalified by an international organization such as WHO. The application should list countries the product is registered or licensed or authorized for use and provide proof/evidence to establish the fact.
6. A list of each site where the product, if authorized, would be (or was) manufactured and the evidence of current Good Manufacturing Practices (GMP) status of the listed manufacturing site(s)
7. Identification of any approved alternative products, including their availability and adequacy for the proposed use (if known)
8. Available safety, efficacy and effectiveness information/data on the medical product (i.e., non-clinical and clinical data)
9. A detailed discussion of risks and benefits balance of the medical product
10. A description of the information for health care providers or authorized dispensers and recipients of the product, (e.g., two separate “Fact Sheets”), and the feasibility of providing such information to health care providers or authorized dispensers and recipients in emergency situations
11. Information on the Chemistry, Manufacture, and Controls – Quality part of the product development dossier. Data should be submitted on the product stability and conditions of storage.
12. Certificate of Analysis of the finished medical product
13. Instructions for use as an EUA product (e.g., if follow-up treatment is required)
14. Proposed product labelling of the medical product. Labeling should at least comply with the WHO labelling requirements for the product. It should include Packaging Insert or Patient Information Leaflet.
15. Proposed Summary of Product Characteristics (SmPC). It should at least comply with the WHO SmPC requirements guidelines.

16. Risk Management Plan (RMP). It should at least comply with the content, format and submission prescribed in the EMA RMP guidelines or the guideline on good pharmacovigilance practices (GVP) - Module V – Risk management systems. Applicants will be requested to incorporate local RMP requirements in the final RMP document (refer to the Guidelines for safety monitoring of medicinal products on the FDA website)
17. Proposed medical product handling, storage and transportation logistics necessary to maintain product integrity.
18. Name of reference substance/material (if applicable).

Before a vaccine will be allowed to be used in Ghana, the product development dossier will have to be evaluated for its quality, safety and efficacy profiles by the FDA, albeit, within the stipulated timeline of 15 working days in the context of the declared public health emergency. A satisfactory outcome will lead to the COVID-19 vaccine authorization for use by the FDA.

Provided a COVID-19 vaccine is authorized for use by the FDA, a consignment of that vaccine will be cleared on arrival at the port upon presenting the evidence of authorization. This process is expected to be completed within a working day. Generally, all COVID-19 vaccine applications will not be subjected to local testing before introduction. However, an incidental occurrence such as cold chain/temperature excursions may warrant testing prior to introduction.

3.3.2 Requirements for importation and clearance at the port of entry

All emergency use authorized COVID-19 vaccines will be cleared at the port of entry. Applicants/importers will be required present the authorization letter that bears the authorization number and the validity of the authorization to the officer at the port of entry.

In addition, the applicant will be required to share with the FDA for the purposes of evaluation, approval and documentation, the summary lot/batch release report and certificate in advance, at least 4 weeks, before the consignment is received at the port.

In some cases, samples of the vaccine may be randomly picked and subjected to selected quality control testing before release. This is will be done on a case - by – case basis.

3.3.3 Reliance

This pathway will be activated in situations where the vaccine has been granted EUA by a well-resourced NRA. A risk-based approach, concentrating on what is locally relevant or critical resources (such as storage conditions, transport, etc.) will be employed. Decisions will be made within 10 working days of submission of registration application.

The requirements for any COVID-19 vaccine registration will be in line with the Public Health Act 2012, Act 851 and applicable guidelines.

The under listed documents should be submitted to the FDA for evaluation towards authorization:

1. An application letter addressed to the Chief Executive Officer of the FDA
2. Completed application form for vaccine registration
3. Product development dossier
    a. Preferably in the common technical document (CTD) format (Modules 1-5)
    b. Evidence of registration and evidence of use in the country of origin
    c. Risk Management Plan (RMP)
    d. A Local Agent (QPPV and Regulatory Contact person)

The Summary batch release report and certificate should be submitted for evaluation by the FDA before a registered COVID-19 vaccine will be imported into the country.

3.4 Safety Monitoring of COVID-19 Vaccines

The safety monitoring system for new vaccines shall employ the under listed strategies:

- Enhanced spontaneous reporting
- Active surveillance of adverse events of special interest (AESIs)
- Post Authorization Studies by Marketing Authorization Holders (MAH)

Activities to be employed by the FDA in collaboration with the Expanded Programme on Immunization (EPI) to promote the safety of vaccine recipients are:

3.4.1Enhanced spontaneous reporting

Enhanced spontaneous reporting system shall be employed for the safety monitoring of all COVID-19 vaccines to be deployed. Training will be provided for healthcare professionals on the identification, management, reporting of AEFIs/AESIs and investigation of serious AEFIs. There will also be dissemination of updated adverse event following immunization (AEFI) monitoring guidelines and tools.

AEFI reporting will also be done by MAHs, healthcare professionals and the general public through the AEFI reporting form, the Safety Watch System and the Med Safety Mobile App.

3.4.2 Active surveillance of AESIs

Active surveillance will be done in selected districts. This may be designed as a cohort event monitoring study or hospital surveillance study. Active case search for AESIs will also be implemented to collect data on pre-defined medically-significant events.

3.4.3 Activities by the MAH

MAHs of COVID-19 vaccines are required to submit underlisted safety related documents in line with applicable FDA guidelines:

1. Risk Management Plans and
2. Periodic Safety Update Reports

MAHs of COVID-19 vaccines will be required to carry out Post-Authorization Safety Studies (PASS) to gather additional data on the safety of the vaccines post-approval to further characterize the events, identify potential risks and investigate missing information with respect to the vaccine.

3.4.4 Data analysis and causality assessment

Progress reports on safety of COVID-19 vaccines will be reviewed by the TAC-VBP and causality assessment performed for serious cases, clusters and AEFIs of community concern and feedback provided to the reporter and the community.

3.5.5 Training and supportive supervision

Training will be provided for all stakeholders involved in the safety monitoring of COVID-19 vaccines in order to ensure the safety of the vaccine recipients and also obtain accurate data for decision. Supportive supervision to ensure that activities are being carried out as expected and data generated will be performed.

3.5.6 Communication and information sharing

Safety information will be shared with relevant national and international stakeholders.

3.5.7 Actors in the surveillance system and their roles

The actors in the surveillance system will include.

- Peripheral level: *Vaccinees or Care Givers*, Community Health Workers, Vaccinators and Facility Focal Persons.
- District and Regional levels: District and Regional Focal Persons, Regional AEFI Investigation Teams.
- Central (National) level: Central Focal Team, National AEFI Investigation Committee and the TAC-VBP.

3.5 Post-market surveillance of authorized COVID-19 vaccines

The post-marketing surveillance of the safety of deployed COVID-19 vaccines will be carried out to ensure that deployed COVID-19 vaccines are handled and stored appropriately and in compliance with the manufacturer's requirements/instruction.

Further, post-market surveillance system in place will ensure that only authorized COVID -19 vaccines are in circulation and that fake/counterfeit products are rapidly identified and controlled. The FDA has a functional inter-departmental operational system (Safety Monitoring, Market Surveillance, Registration and Laboratory Services Departments) for processing vaccine safety and quality claims either reported or identified during surveillance activities.

3.6 Summary

Procedure for Adverse Events Following Immunization (AEFI) monitoring of EPI vaccines exist through the use of investigations forms from regions through national EPI to the Food and Drugs Authority. AEFI surveillance for COVID-19 will be incorporated into the existing procedure through the review and re-design of the existing tools to incorporate the COVID-19 surveillance data and health workers will be trained to include this in the normal AEFI surveillance system.

Capacity building sessions for health workers on AEFI (Adverse Events Following Immunizations) will be conducted. Emergency drugs for AEFI management will be available at each post. Supervisors at all levels will monitor investigation and management of AEFIs during the campaign.

Investigating serious AEFI and responding to AESI that are identified through different systems (passive and active surveillance) are essential, with important roles played by different stakeholders gathering key information during the process. Ghana's team is adequate and prepared for causality assessment for AEFI and specific specialized analyses for AESI. Feedback on findings of the investigation and causality assessment will be communicated to all stakeholders including the reporting health worker and the patient.

The country team will address concerns of health care professionals and maintain community confidence by creating and sharing a COVID-19 vaccine safety communication plan with relevant stakeholders. Train and support personnel at all levels to address concerns that may arise before, during and after COVID-19 vaccine introduction. Develop, print, and distribute messages concerning the safety COVID-19 vaccines.

Figure 3 *:AEFI flow and channel*

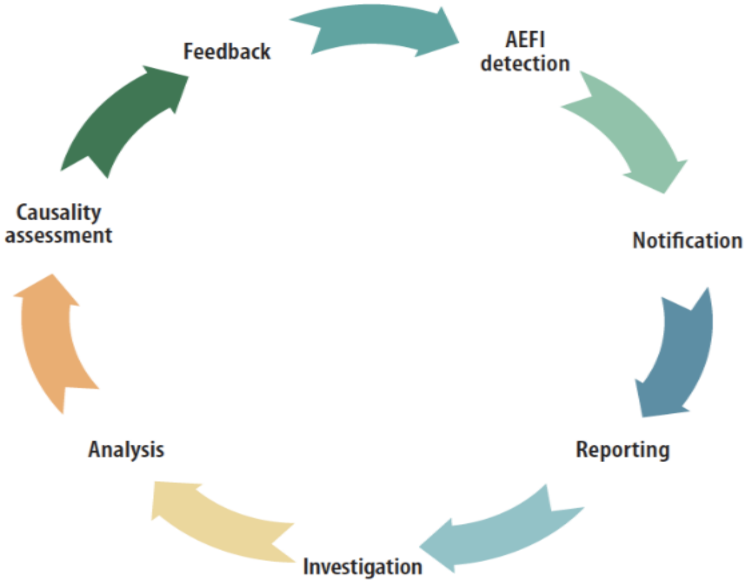

CHAPTER FOUR:
VACCINATION STRATEGIES

4.1 Overview of strategies

Ghana is expected to receive adequate quantities of the vaccine to cover about 20% of the population at the initial phase of deployment through the COVAX facility. The Government of Ghana will explore other opportunities to arrange for additional doses to cover the rest of the eligible population. Given that this initial supply will not be enough for the whole population in the country, the strategy is to target population sub-groups using a risk-based approach and subsequently roll out the exercise to all segments of the population depending on the availability of additional vaccines and the evolution of the pandemic.

The main goal of the strategy is to define target populations and ensure equity in vaccine access.

4.2 Vaccine choice

There are several vaccine development platforms for COVID-19. Currently, efficacy and safety data from advanced clinical trials in different sub-populations are available for different vaccines. The ultimate choice(s) of a COVID-19 vaccine will be aligned to the safety and efficacy profile in the context of the country and population characteristics, cold chain and other ancillary logistic requirements. The NITAG has provided an initial list of prioritized vaccines after reviewing the available data and evidence (Table 3). The introduction of these new vaccines will offer the opportunity to strengthen Ghana's health system and improve the existing immunisation infrastructure and service delivery. The country will critically explore other vaccine platforms to complement the initial choice that will be made.

Table 3:COVID-19 vaccine platforms and characteristics

Manufacturer	Vaccine candidate	Type	Doses	VE[5]	Storage Temp	Age range of trial participants
University of Oxford/AstraZeneca	AZD1222	Viral vector (NR[6])	2 doses, (0, 28d)[7]	70% (90% in sub-group with LD[8])	+2 – +8ºC	≥ 16 years
Moderna TX, Inc.	mRNA-1273	mRNA	2 doses, (0, 28d)	94.5%	-20ºC	18 – 56 years
Pfizer, Inc/BioNTech	mRNA-BNT162	mRNA	2 doses, (0, 28d)	95%	-70ºC	16 – 85 years

[5] *Vaccine efficacy*
[6] *NR – non-replicating*
[7] *Interval between doses*
[8] *LD- low dose (half standard dose)*

4.3 Vaccine deployment scenarios

Population sub-groups have been segmented based on recommendations by the NITAG and SAGE. The segmentation was based on vulnerability, business continuity and national security.

Two scenarios are proposed for the national deployment. The scenarios are premised on vaccine characteristics, available quantities, delivery schedule, human resource capacity, procurement and supply chain management. A phased approach will be used for the deployment of the vaccine according to the segmented population simultaneously across all the sixteen (16) regions if there are adequate vaccines. In an instance where vaccine supply is inadequate, targeted populations in high burden regions or hot spots will be prioritized.

The first segment of the population that will benefit from the initial supply of up to 3% of vaccines will be health workers (0.65%), personnel of the security services (0.3%) and persons with known underlying co-morbidities in identified COVID-19 hotspots (2.05%). Persons with known underlying co-morbidities in other areas other than hotspots (1.9%), adults 60 years and above (6.5%), tertiary and second cycle students, teachers at all levels (7.9%) and other essential workers within identified COVID-19 hotspots (0.7%). The rest of the population would be covered by vaccines from the COVAX platform and other sources.

Scenario 1: Assumption is that the country has adequate vaccine doses, ancillary logistics and operational capacity. This deployment is expected to be completed in 5 months (Table 9)

Scenario 2: This scenario envisages global vaccine supply over a longer period and hence vaccine deployment will be staggered in 3 phases (Table 11).

Table 4: Segmented population groups and justification

Segmented population	Estimated proportion (%)	Population	Justification
1. Health care workers[9] (clinical and non-clinical)	0.65	206,338	Health care workers provide critical medical care (in and outpatients), emergency care and immunisation. They are at high-risk of exposure and occupational infection and spread. Loss of a critical number of HCWS can adversely affect the quality of the health delivery system.
2. Security Service[10]	0.3	95,233	Security services are at the core of the country's national security and will be relied on should a major disaster (health and other natural disasters)

[9] *Consideration will be given to volunteers and social mobilizers who will support the vaccination.*
[10] *Prisoners to be considered as part of this group*

Segmented population	Estimated proportion (%)	Population	Justification
			occur. Safeguarding their health is paramount. Security personnel also interact with large segments of the population in the execution of their duties and are at high risk of exposure, infection and disease transmission.
3. Persons with known underlying com-morbidities [11](Hypertension, Diabetes Mellitus, Asthma/COPD, Sickle Cell Disease etc.)	3.95	1,253,903	Individuals with underlying medical conditions are within the highest risk bracket of disease morbidity and mortality. Protecting this group would reduce incidence and mortality
4. Adults 60 years and above	6.5	2,063,385	Older persons are at high risk of severe disease and mortality
5. Tertiary and second cycle students, teachers at all levels	7.9	2,507,806	Tertiary and second cycle students and teachers are mostly confined in their various campuses. Exposure to large populations at a given time and are at risk of high exposure and infection
6. Essential service providers (ECG, GWCL, UN staff and other DPs, Bankers and professions in contact with large populations at a time (market women, commercial drivers etc.)	5.0	1,587,219	People in this category provide essential services which includes coming into contact with people, providing meals and utility services. Exposure to large populations at a given time and are at risk of high exposure and infection.
7. Special group on national assignments (contact sports e.g. football, boxing etc.)	0.2	63,489	Potential for focal outbreaks during tournaments, travel etc.
8. Executive, Judiciary and Legislature, Ministries & Civil service	0.3	95,233	It is important to ensure that the governing structure of the country is protected. The leadership of the country are engaged in several physical meetings to advance the course of the country. Exposure to large populations

[11] *To consider persons with disability*

Segmented population	Estimated proportion (%)	Population	Justification
			at a given time and are at risk of high exposure and infection
9. Media	0.2	63,489	Exposure to large populations at a given time and are at risk of high exposure and infection
10. Other members of the population excluding children under 16 years and pregnant women	30	9,523,313	General risk of exposure and infection. Important to build herd immunity
**Total**	**55.0%**	**17,459,408**	

4.4 Strategies for vaccine delivery

The overall vaccine deployment strategy will be hinged on vaccine availability, vaccine choice, country context and the characteristics of the segmented populations. The country will leverage on existing vaccination strategies (fixed, temporary and mobile sites) and lessons from previous mass vaccination campaigns to deliver the vaccine to the targeted population. The human resource requirement using the different strategies to reach all the eligible population for the different deployment scenarios are provided in Table 10 and Table 12.

The MOH will explore other strategies based on current best practices and adapt to the country context to effectively reach all the targeted population. The MOH will use the existing cross-sectoral collaboration platforms to support the deployment of the vaccine including integration with other health programmes.

The private sector will play a key role in supporting effective vaccine deployment. The MOH will develop a collaborative platform to engage the private sector. The aim is to ensure that the private sector fully participates and align with the national COVID-19 vaccine deployment plan.

Adequate quantities of emergency drugs will be provided at all vaccination posts and all healthcare workers involved in the administration of the vaccine will be appropriately trained to promptly manage potential adverse events such as severe anaphylactic reactions. Deployment will take into consideration other special and vulnerable population (persons with disability, prisoners, etc.) that were not captured under segmented population. Strategies will be adopted to ensure that they are included in the appropriate phase of the vaccine deployment.

The vaccination phase which targets the rest of the population will require additional healthcare workers to support the vaccine deployment as well as re-training. Based on the quality and

coverage of the different phases of the vaccination, appropriate mop-up strategies will be adopted to cover missed populations.

4.5 Integration and continuation of routine immunisation services

Vaccination of all the targeted population is likely to take between 3 – 5 months and may interrupt the delivery of routine immunization services and other healthcare services. To ensure the continuous provision of essential services, all fixed and outreach vaccination service points will dedicate days to integrate routine immunisation activities during the campaign. Health facility managers will be required to develop strategies to re-organize staff to ensure that both routine health care services and the campaign are seamlessly integrated.

There are plans to leverage the additional infrastructure, resources and capacity improvement that will accompany the COVID-19 vaccine deployment. This will improve coverage for all antigens especially in the hard-to-reach areas and urban centers which account for a significant proportion of unimmunized children in the country. This integration will also optimize the use of the drone technology to supplement rapid delivery of vaccines to areas within the catchment area of the Zipline drone technology which has been complementing service delivery of vaccines to hard-to-reach areas.

The identification of vulnerable groups especially those with co-morbidities and the aged would improve the existing database for such groups to aid continuous surveillance, follow up and the development of tailor-made interventions that will improve their overall health towards the attainment of improved Primary Health Care (PHC) as part of the Universal Health Care (UHC) targets.

Table 5: Vaccine deployment schedule (Scenario 1)

Phase	Targeted Pop	Period/Duration[12]	Target population	Proposed dates
Phase 1	Segmented population	2 months<br>Each round lasting for at least 14 days.	7,936,095	**R1:** 14 – 28 April 2021<br>**R2:** 12 May – 02 June 2021
Phase 2	Other members of the population excluding children under 16 years and pregnant women	2 months<br>Each round lasting for at least 14 days.	9,523,313	**R1**: 07 – 21 July 2021<br>**R2**: 04 – 25 August 2021

[12] *2 rounds for 2-dose vaccine with a minimum of 28 days between doses*

**Total**	**17,459,408**

Table 6: Human resource needs for vaccine deployment (Scenario 1)

Phase	Targeted population	Number of Teams				Number of Persons		
		Static	Mobile	Outreach	Total	Vaccinators	Volunteers	Supervisors[13]
Phase 1	7,936,095	1890	1890	1890	5669	5669	17006	945
Phase 2	9,523,313	2267	2267	2267	6802	6802	20407	1134
**Total**	17,459,408	4157	4157	4157	12471	12471	37413	2079

**280 VAMs[14] will be deployed during each round of vaccination*

Table 7: Vaccine deployment schedule (Scenario 2)

Phase	Targeted Population	Period/ Duration[15]	Target population	Proposed dates
Phase 1	• Health Care Workers<br>• Security Personnel<br>• Persons with known underlying medical conditions	2 months<br>Each round lasting for at least 14 days.	1,555,475	**R1:** 14 – 28 April 2021<br>**R2:** 12 May – 02 June 2021
Phase 2	• Adults above 60 years<br>• Secondary and tertiary students & all teachers<br>• Essential service providers<br>• Specialized groups on national assignment<br>• Executive, legislature & Judiciary, Ministries, Civil Service<br>• Media personnel	2 months<br>Each round lasting for at least 14 days.	6,380,620	**R1:** 23 June – 07 July 2021<br>**R2:** 21 July – 11 August 2021
Phase 3	1. Other members of the population excluding children under 16 years and pregnant women	2 months<br>Each round lasting for at least 14 days.	9,523,313	**R1**: 01 – 15 September 2021<br>**R2:** 29 September – 20 October 2021

[14] *VAM – Vaccine accountability monitors*
[15] *2 rounds for 2-dose vaccine with a minimum of 28 days between doses*

**Total**	**17,459,408**

Table 8: Human resource needs for vaccine deployment (Scenario 2)

Phase	Targeted population	Number of teams				Number of Persons		
		Static	Mobile	Outreach	Total	vaccinators	volunteers	Supervisors[16]
Phase 1	1,555,475	370	370	370	1111	1111	3333	185
Phase 2	6,380,620	1519	1519	1519	4558	4558	13673	760
Phase 3	9,523,313	2267	2267	2267	6802	6802	20407	1134
**Total**	**17,459,408**	**4157**	**4157**	**4157**	**12471**	**12471**	**37413**	**2079**

**280 VAMs will be deployed during each round of vaccination*

Table 9: ***Vaccine deployment strategies***

Target groups	Potential delivery strategy	Potential vaccination sites
Health workers	• Fixed sites	Health centres, Hospitals (public and private)
Persons (60 years and above)	• Fixed and outreach sites<br>• Temporary/mobile clinics<br>• Mass campaigns	Health Centres, Hospitals, Outreach points, pharmacies, marketplace and other public places, drive-through.
Persons with underlying medical conditions	• Fixed sites and outreach sites<br>• Temporary/mobile clinics	Primary health care facilities, outpatient clinics, hospitals, workplaces, through mobile teams for those with underlying medical conditions confined at home, other public and private establishments
Other targets groups	• Fixed site and outreach sites<br>• Temporary/mobile clinics<br>• Mass campaigns	Any of above plus special strategies to reach specialized population groups

[16] *Excluding national, regional and district level supervisors*

4.6 Defaulter tracing and reminders

The Government intends to go fully electronic in terms of data capture on vaccinees. There will be an in-built defaulter tracing mechanism as part of the development of the system. Data on persons who are due for the next dose will be automatically generated and followed-up.

Additionally, electronic print-out of vaccination will issued to vaccinees and reminders will also be sent through text message.

4.7 Infection Prevention and Control (IPC) measures

As recommended by WHO, Ghana will ensure that existing IPC policy, guidelines and protocols are adhered to in providing immunization services. The MOH will ensure adequate access to appropriate PPE and related WASH logistics to enable health workers to adhere to COVID-19 safety protocols. There will be continuous capacity building of HCWs to ensure optimal adherence to all preventive and safety protocols during the deployment of the vaccine.

4.8 Vaccination data management

Robust data collection and management systems and protocols will be developed by the data management sub-committee to ensure that accurate data is generated to guide decision making. These will include provision of standardized list of legal identification documents to verify demographic characteristics of vaccinees, data collection tools (preferably digitized), systems to identify already vaccinated individuals, defaulter tracing system to track and remind second-dose defaulters amongst others.

CHAPTER FIVE:
DEPLOYMENT SYSTEMS AND MODALITIES

5.1 Legal basis

The vaccines will be deployed in accordance with the legal structures as provided in the Public Health Act 851, 2012 and with oversight enforcement by the appropriate state agencies. The Food and Drugs Authority (FDA) will ensure the safe delivery of the vaccine through existing framework for vaccine registration, safety and regulation in collaboration with partners.

5.2 Existing Health Infrastructure

The vaccine will be deployed using the MOH/GHS decentralized administrative and service delivery structures. The Expanded Programme on Immunization (EPI) under the Public Health Division will have the oversight responsibility for vaccine deployment in collaboration with other programmes and divisions.

The EPI will leverage on existing immunization infrastructure at the different levels with opportunity to expand and develop new infrastructure to strengthen the vaccination programme beyond the pandemic.

5.3 Training

Training and microplanning should be completed not more than thirty (30) days before vaccine deployment (not later than 15 March 2021). Ghana currently has adequate health workforce for routine immunization delivery service. However mass vaccination campaigns will require support from community volunteers, private providers and CSOs.

The Training and Service Delivery sub-committee will plan and develop training manuals, guidelines and review relevant existing vaccination tools for the purpose of introducing the COVID vaccine. Managers, supervisors and vaccinators will be trained on the new vaccine. Cascade strategy will be utilized to train service providers at all levels. A Trainer of Trainers (TOT) workshop for National and Regional level staff will be conducted at the central level. Subsequent cascade training will be decentralized to regions and districts.

All training modules will be adapted and contextualized from generic training materials for COVID-19 vaccine deployment developed by WHO. The methodology used for the training will be presentations, plenary discussion, question and answer sessions, role play, group work, demonstration and practice sessions.

Topics to be covered in the training will include:

- Background & epidemiology of COVID-19 disease and rationale for vaccination
- Brief information on the COVID-19 vaccine
- Objectives of the vaccination programme
- Communication
- Infection Prevention and Control measures
- Logistics and cold chain management
- Vaccine accountability and monitoring
- Conducting quality vaccination sessions
- Handling of the vaccines and logistics
- Injection safety and waste management
- Recording and documentation
- Monitoring and management of AEFI and AESI

All trainings will be restricted to smaller groups with a mix of virtual and in-person practical approach. This will ensure adherence to COVID-19 preventive protocols and adequate acquisition of knowledge and skills. Table 15 shows the categories of cadres identified for training:

Table 10: *Cadre of staff for training*

Employment Category	Estimated Total
Allied Health Professionals	10351
Doctors	4787
Health Intern	7908
Management Staff	375
Nurses and Midwives	83353
Other Clinical Staff	4244
Community Pharmacists	4868
Support Staff	38982
Volunteers	16,412
**Grand Total**	**171, 280**

5.4 Monitoring and Supportive supervision

GHS will use the existing supportive supervision strategy in the deployment of the COVID-19 vaccine. Supervisors would be deployed prior to the implementation phase to ensure micro plans, trainings, logistics etc. are in place before the campaign. Supervision will continue during the implementation phase to ensure the overall quality and success of the campaign. The EPI

guideline for supervision (*see Annex X*) will be adapted for monitoring and supervision. The data management team will develop a post-campaign evaluation methodology in alignment with global best practices and standards.

CHAPTER SIX:
IMMUNIZATION MONITORING SYSTEMS

6.1 Data needs

As Ghana prepares to introduce COVID-19 vaccine, there will be high demand for data by public health decision-makers and other stakeholders viz; national and subnational authorities, the public, communities, civil society organizations, the press; national, regional and global immunisation partners, including donor organizations; and vaccine manufacturers and regulatory bodies, health researchers and academics.

To meet key anticipated needs of these different stakeholders, Ghana's immunisation programme will design a monitoring system for COVID-19 vaccines and vaccination that is able to:

a. Measure equitable uptake and coverage over time by geography, population groups, and risk groups.

b. Monitor extent of national policies to segment at-risk groups and settings (e.g. hospital, health centres, islands and riverine communities).

c. Produce a personal vaccination record/certificate for any health, occupational, educational and travel purposes (as per national policies).

d. Ensure necessary records and documentation are in place for use in surveys, safety monitoring, disease surveillance and vaccine effectiveness studies etc.
e. Monitor full course of response in the likely case a multidose vaccine schedule is required, to reduce incidence of drop-outs and also enable follow-ups

To address these needs, an electronic vaccination registry has been proposed. Ghana piloted an ORACLE®[17] cloud database supported system during the Yellow Fever Preventive Mass Vaccination Campaign (Phase B, November 2020). Discussions are ongoing with other partners notably Simprint®[18] to provide an enhanced platform for a vaccination registry. An electronic vaccination registry will leverage off existing systems which are currently deployed and implemented at scale, thus replace paper-based systems.

Already the country uses DHIS2[19]s' e-tracker model and is being considered for COVID-19. Synergies among these platforms will be explored and adapted for optimal use.

[17] https://www.oracle.com/corporate/citizenship/health/
[18] https://www.simprints.com/
[19] https://www.dhis2.org/

This proposed electronic vaccination registry will support collection and provision of the following information;

a. **Client information**; demographics (age, sex, contact address, number of doses, etc.
b. **Health Facility details** where service is accessible (name and type, e.g. clinic, level,)
c. **Vaccine administered** (manufacturer, batch number, diluent batch number, etc.)
d. **Safety information** as part of a pharmacovigilance plan (Adverse Events Following Immunization – AEFI)
e. **Proof of vaccination, a record of vaccination issued to individuals**

6.2 Indicators to Monitor Progress

Indicators to measure progress with COVID-19 vaccines and vaccination will be similar to those used for routine immunisation and vaccination campaigns.

Vaccine uptake: The number or proportion of persons vaccinated with a certain dose of the vaccine in a certain time period (e.g. during a month or year). These will be expressed as a percentage to provide coverage rates.

Vaccination coverage: The vaccinated proportion of the target population. This is similar to uptake but considers vaccination in previous time periods. Depending on the time period, uptake and coverage can be used interchangeably.

The system will track and represent uptake of COVID-19 vaccines as COVID, by dose as follows:

- COVID-1: The number of people receiving a first dose of the vaccine, or the proportion of a target group that did so. For example: 50 000 doses of COVID-1, corresponding to 5% of the total population;
- COVID-2,: The number or proportion of people receiving a second dose of the vaccine.
- Drop-out from 1st and 2nd dose: The proportion of persons who received at least one dose of a COVID-19 vaccine but did not receive the last dose in the schedule yet. Calculated as:
  [(1st Dose – 2nd Dose)/1st Dose] x 100

Vaccine uptake will be tracked and evaluated periodically.

6.3 System to record, report, analyze and use vaccination data

COVID-19 vaccines uptake will be monitored through an “administrative system” and or evaluated through household surveys. Persons vaccinated will keep and access a record of their

status history in a digital "Health Wallet" app on a smart phone. Clients without a smartphone receive a paper-based version of personal record containing a QR (Quick Response) code to scan in order to access their details. With a digital health wallet directly connected to an affiliated laboratory or institution, users are able to demonstrate their vaccination status when required, eg returning to school or work, travelling abroad etc. Alternatively, Ghana's paper Vaccination Card (Yellow Card) meets international standards and may be use for this purpose.

6.4 COVID-19 vaccination dashboard

The country will develop a COVID-19 vaccination dashboard to provide insights into a variety of programmatic aspects. This will aid real-time decision making by programme managers and field coordinators.

The COVID-19 vaccination dashboard will be build based on experiences during previous vaccination campaigns (mOPV2, YF and IPV). During these campaigns, the country deployed an agile dashboard that supported and provided insight information to all stakeholders.

The dashboard will focus on key performance indicators, for the following:

- **Precampaign**: service availability and readiness (human resource capacity, cold chain and supply, training, social mobilization, funding, local government involvement, etc);
- **During Campaign:** vaccine uptake and coverage by geography, population groups, and risk groups, and over time series, safety monitoring & AEFI, refusals, disease surveillance, preventive protocols, vaccine wastage, drop-out, VVM stages cold chain, etc
- **Post Campaign:** coverage surveys, AEFI, Post-Introduction Evaluation (PIE)

6.5 Timelines

Table 11: Timelines for Data Monitoring

Activity	Expected Output	Timeline
Engagement meeting with software partners & DHIS2 team	Harmonized/synergized platform for use	TBD
Sub-committee meetings	Hold 12 meetings	Weekly (every Wednesday)
Review and adapt tools for COVID-19	Recording and reporting tools adapted	01- 05 Feb 2021
Develop dashboards	Dashboard developed	08-12 Feb 2021

Pilot	Draft tools and dashboard piloted	3rd week Feb 2021
Training	Committee members<br>Cascaded<br>• National, regional, districts, etc	1st week Mar 2021<br><br>2nd& 3rd week Mar 2021
Deploy tools		4th Week Mar 2021

CHAPTER SEVEN:
OPERATIONAL RESEARCH

7.1 Background

COVID-19 is a novel disease; hence its vaccines are new with little or no information at the population level. Research into vaccine deployment for emerging and re-emerging infectious diseases is important in driving implementation strategies. As Ghana plans to roll out COVID – 19 Vaccine, it is essential to conduct studies at pre-deployment phase, deployment phase and post deployment phase to guide the Ministry of Health/ Ghana Health Service in taking evidence -based decisions. In line with this, research will be an integral component of the COVID-19 vaccine deployment plan for Ghana.

The goal of the research thematic group is to promote social, behavioral and biomedical research studies into COVID – 19 vaccine deployment to improve safety, coverage and policies on COVID -19 vaccine deployment.

The specific objectives are;

1. Provide guidance on priority areas for research on COVID-19 vaccine in Ghana
2. Provide support in mobilizing resources to support operational research on COVID -19 vaccine and vaccination in Ghana
3. Monitor implementation of COVID-19 vaccine related research in Ghana
4. Provide opportunities for the dissemination of COVID-19 vaccine related research

7.2 Guidance on priority areas for research

A central coordinating group will be constituted to coordinate operational and long-term research on COVID-19 vaccination in Ghana. Timely dissemination of research finding will be undertaken to ensure Ghana contributes to the knowledge base on COVID-19. In line with risk communication, the findings of operational research will inform the crafting of appropriate information to be communicated to the general population as far as COVID-19 vaccination is concerned. Research would employ both qualitative and quantitative research methods. Areas of research would include but not limited to;

Pre-deployment phase

a) Knowledge and information on COVID-19 vaccine among health care workers
b) Knowledge, attitude and practices (KAP) of the general population on COVID – 19 vaccination

Deployment Phase

a) Exploration into factors contributing to COVID – 19 vaccine refusal

Post Deployment phase

Short to mid-term studies

a) Strengths and challenges of health delivery system in the provision of COVID – 19 vaccination services in Ghana
b) Seroconversion rates among recipients of COVID – 19 vaccines

Long Term studies

a) Impact of vaccination on the general population
b) Impact of delayed second dose on immunity to COVID-19
c) Effect of the vaccination of breastfeeding mothers on infants
d) Effect of COVID-19 vaccination on SARS-CoV-2 viral shedding

7.3 Remobilizing resources to support operational research

To support research activities, a research fund will be created at the Research Division of the Ghana Health Service with seed money to provide grants to researchers to support operational research on COVID-19 vaccine roll-out. Proposals will also be shared with collaborating agencies of Ghana Health Service to support research areas of national interest. The team would also search for Notice of Funding Opportunities (NOFO) which are in line with the research areas and submit applications for consideration

7.4 Opportunities for dissemination of COVID-19 vaccine related research

The dissemination of research through publications, dissemination meetings and conferences will be essential in further validation of research findings and the application of findings to implement policy and practice. In line with this, there will be organised dissemination meetings and conferences by the MoH/GHS on research related to COVID-19 vaccine and vaccination activities.

7.5 Monitor implementation and dissemination of research findings

A system for monitoring and synergizing research activities across all regions will be established. We plan to achieve this by engaging the various ethical review committees to build a database of ongoing and completed research on COVID-19 vaccine and vaccination in Ghana. In addition, the Ethical Review committees will be engaged to help expedite review of proposals where need be.

7.6 Timelines

Table 12: Timelines for Research

Description	Start Date	End date
Pre-deployment phase research activities	February 2021	March 2021
Deployment Phase research activities	TBD	TBD
Post Deployment phase research activities	TBD	TBD

CHAPTER EIGHT:
COMMUNICATION AND INFORMATION

8.1 Introduction

Communicating about COVID-19 Vaccine is critical in order to ensure acceptability and reduce misconception. This communication plan will draw lessons from previous vaccine introductions such as H1N1and most recently, the Malaria Vaccine introduction in Ghana. Key messages and approach will rely mainly of various researches conducted on COVID-19 disease and vaccination. A comprehensive communication strategy on COVID-19 Vaccine is being finalized by the Communication Sub Committee on COVID-19 Vaccine deployment. Key highlight of the strategy are:

8.2 Goal

The overall goal of this strategy is to ensure accurate understanding of the benefits of COVID-19 vaccination and alleviate apprehension and ensure its acceptance and encourage uptake across various audiences.

8.3 Expected Outcomes

1. Appreciable level of acceptability and accessibility through demand creation activities
2. A clear-cut information on COVID-19 vaccination that will be understood by everyone
3. Efficient and effective management of rumors, myths and misinformation
4. A well-coordinated community engagement mechanism to promote COVID-19 Vaccination
5. Enhanced Partnership

8.4 Objectives

a. Create demand, promote acceptability and accessibility of COVID-19 vaccine among at least 90% of the general public.
b. Develop appropriate messages and identify channels to communicate the potential benefits and risks of the vaccine to all concerned parties, including decision-makers at all levels.
c. Provide timely and accurate information to address misinformation, rumors and other crisis situations.
d. Effectively mobilize and empower communities to ensure participation and ownership of the vaccination process

e. Strengthen communication mechanisms and partnerships among key stakeholders to support the national communication effort.
f. Build public confidence on safety and efficacy of the new vaccine to prevent vaccine hesitancy
g. Promote COVID-19 appropriate behaviour including adhering to preventive protocols – social distancing, washing of hands and wearing of mask.

8.5 Strategic Approach

Variety of strategic approaches will be used. These include;

a) Identify and prioritize audiences and stakeholders.
b) Make effective use of multiple and diverse channels to deliver information
c) Focus on clear, evidence-based messages tailored to audience needs
d) Develop and pretest COVID-19 vaccine educational materials among target audiences (including FAQs, flip charts)
e) Collaborate with relevant MDAs, NGOs, CBOs and other civil society groups to roll out the national communication campaign to all levels
f) Conduct Target Audience Analysis- e.g., use Human-Centered Design (HCD) to identify audience and appropriate messaging.
g) Conduct stakeholder mapping to identify relevant stakeholders and partners across all levels
h) Identify and train multiple spokespersons at different levels of care and interactions with public
i) Adopt a multi-level integrated approach that will combine a variety of mutually reinforcing channels of communication to build synergy and strengthen partnerships, programs and activities.
j) Conduct impact analysis on educational materials to establish the preferred and effective material to be adopted for awareness raising engagements.
k) Collaborate with key personalities/celebrities and influencers (who would be first recipient of vaccines) as ambassadors and their endorsements to raise awareness on vaccine deployment and usage.
l) Launch of vaccine deployment campaign.

8.6 Proposed Channels and Measures

Varied channels will be adopted to ensure that appropriate information is communicated to the right people at the right place and at the right time. Some **measures** will include;

a. Sensitization activities on COVID-19 vaccine in every health facility.
b. Segmented groups for vaccine deployment need transparent public debate to build support for ethical principles, risks, recommendation and preventive measures.
c. Work closely with partner agencies, representatives of local communities with critical populations, to achieve consensus on actions, consistency in messages, and coordinated communication activities.
d. Health Policies on COVID-19 immunization need to be coordinated and decentralized manuals developed to support local authorities and community engagements.
e. Collaborate with the media to ensure that the general public is well informed on COVID-19 vaccines.
f. Train media personnel with the right and essential information on COVID 19 vaccines deployment and usage.
g. Develop job aids for health workers
h. Develop audio visual materials including posters. Leaflets, jingles, recorded messages on survivors lived experiences with COVID 19 (testimonials) and technical briefs for advocacy
i. Conducting social announcements via Community Information System (including mobile vans, market, religious places
j. Raise awareness via social media
k. Use traditional announcement channels including 'gong-gong' beating; folklores.
l. Conduct child-to-child education on COVID 19

8.7 Crisis Communication approach

8.7.1 Purpose

The purpose of this approach is to prevent information about COVID 19 vaccines from resulting in a potential negative impact on acceptance of COVID 19 vaccines and deployment. In case of any crisis, this approach would aim at minimizing the impact of a potential crisis on the COVID-19 vaccine and to build a crisis communication team to respond to potential and imminent crisis situations. A crisis communication strategy has been developed and can be found in the National Communication Strategy for COVID 19 vaccine deployment (Chapter 4, page 23-30).

8.7.2 Monitoring and Evaluation

Monitoring of communication activities will done at all levels guided by a technical monitoring and evaluation plan.

CHAPTER NINE:
SUPPLY CHAIN PROCESSES

9.1 Cold chain capacity

The current capacity of the cold chain in the country is inadequate to support and sustain the deployment of COVID-19 vaccine. In addition, Ghana is in the process of expanding the available cold chain capacity through the Cold Chain Equipment Optimization Project (CCEOP) to improve the cold chain capacity at the district and health facility level. However, there is still the need to increase the cold chain capacity at the National level and regional level especially the six new regions. The cold chain equipment available in districts and health facilities will be used.

There is adequate human resource for cold chain management at national, regional and district levels. These levels have designated officers who are in-charge of vaccine and cold chain.

9.1.1 National Level

A comprehensive cold chain inventory was conducted in 2018. This has recently been updated in 2021. The central store is currently equipped with four (4) 40m3 positive walk-in cold rooms (WICR) with a net capacity of 35,700 litres. The required net storage capacity to accommodate the current vaccines is 48,334 liters. Considering the introduction of the COVID-19 vaccines, there will be an added requirement of 15,925 litres of positive cold storage. Therefore, there will be the need for additional two 40m3 positive walk in cold rooms at the National Level. (Refer to attached Ghana Cold Chain Supply Chain Sizing tool). Out of the four existing cold rooms, two of them are very old (more than 25 years) which incurs frequent breakdown and would require replacement. Hence, four new 40m3 walk-in cold rooms are required (two replacement and two additional (expansion)).

There is a separate 20m3 walk-in freezer (WIF) available with insufficient volume for Oral Polio and Rotavac vaccine storage and may require additional 40m3 WIF (-20°C) to cater for any COVID-19 vaccine that may require negative storage temperature. Ultra-cold chain facilities will be installed at the national level in anticipation of receiving vaccines that must be stored in such temperatures.

Additionally, there is a standby generator of 100KVA which is enough to power all the existing and new walk-in cold rooms that may be installed.

Injection materials like AD syringes and mixing syringes will be estimated with a wastage rate of 10%. The COVID-19 vaccines would be calculated with a wastage rate of 10%.

Personal protective equipment (PPE) including face masks and hand sanitizers have also been estimated.

9.1.2 Quantifications

Table 13: Cold Chain Equipment requirements for +2⁰c to +8⁰c and -20⁰c and accessories

Level	Item	Capacity	Quantity
National	WICR	$40m^3$	4
National	WIF	$20m^3$	1
Regional	WICR	40m3	7
Regional	WIF	20m3	7
District	Refrigerators	100-200 litres	43
District & Health facility	Stabilizers		686
Health facilities	Refrigerators	100 litres	619

Note: Investment in the cold chain equipment would boost EPI cold chain capacity even after COVID-19 vaccine deployment. The cost of procuring the above equipment is estimated at USD1,571,059.80

Table 14: Cold Chain Equipment Requirements for -70⁰c and accessories

Level	Item	Capacity	Quantity
National	Freezer	895L	5
Regional	Freezer	895L	4
Regional	Power Generators	50KVA	7
Regional Hospital	Freezers	567L	16
Teaching Hospital	Freezers	567L	5
Regional Hospital and Teaching Hospitals	Stabilizer		21

Note: The cold chain equipment are not used in EPI routine services and therefore would not be in use after the covid-19 vaccine deployment. The cost of procuring the above ultra-cold chain equipment is estimated at USD500,000

Table 15: Summary logistics

Item	Target pop.	Wastage Rate	Quantity	Unit of Measurement	Comments/Assumptions
Vaccines	17,459,408	1.1	7,682,134	vials	wastage rate of 10%, 10 dose per vial and 2 dose schedules in vials
AD syringes	17,459,408	1.1	38,410,698	pieces	wastage rate of 10% and calculated in doses
Cotton Wool (500g)	12,471	1.1	54,872	rolls	4 rolls of cotton wool per team with wastage rate of 10%
Safety Boxes	17,459,408	1.1	384,107	pieces	100 AD syringes per safety box with wastage rate of 10%
Hand Sanitizers	60,000		240,000	bottles	4 per worker with wastage of 10%
Face mask	60,000		33,600	boxes	28 per worker with 10% wastage rate in 50 per box
Vaccination Cards	17,459,408	1.1	19,379,943	pieces	1 per target pop. Including 10% wastage rate.
Emergency medicines (adrenalin or Hydrocortisone)	12,471	1.1	13,718	Ampoules	1 per team including wastage rate of 10%

9.1.3 Regional Level

Two regional stores (Northern and Greater Accra) are equipped with 80m3 WICR. Three regions namely Central, Ashanti and Bono are equipped with 40m3 WICR each while five regional stores Upper West, Upper East, Eastern, Western and Volta are equipped with a 30m3 WICR. These ten regional stores have adequate storage capacity to accommodate current and additional vaccines (COVID-19).

Greater Accra and Ashanti regions have Walk-in Freezers (WIFs) that have temperatures up to -20°C. All other regions will require WIFs should the country decide to go in for COVID-19 vaccines that will require storage at -20 temperatures. Should the country opt for vaccines requiring ultra-negative temperatures, all regions will require a revamp in the cold chain system.

The six newly created regions do not have regional cold rooms for storage and distribution of vaccines to the lower levels at all and rely on sister regions. Hence, it will be critical to establish walk-in cold rooms prior to the deployment of COVID-19 vaccines. Each region would require one 40m3 WICR and one 20m3 WIFR. Additionally, each of the cold rooms would require 50KVA standby generator.

The Central regional cold room has been experiencing frequent breakdown with high maintenance cost. Hence, there would be the need to replace the existing WICR.

8.1.4 District Level

The country currently has 260 districts out of which 44 are newly created districts. Through the support of the GAVI CCEOP, 2,343 CCE have been deployed into the 260 districts to boost the Cold chain capacity at the lowest distribution points (districts) and service delivery points. However, the country still has 15 districts with inadequate cold chain capacity. Each of these districts should be provided with CCE with 145 litre capacity.

Figure 4:Segmentation of Health Facilities (Lowest Distribution Points – District level)

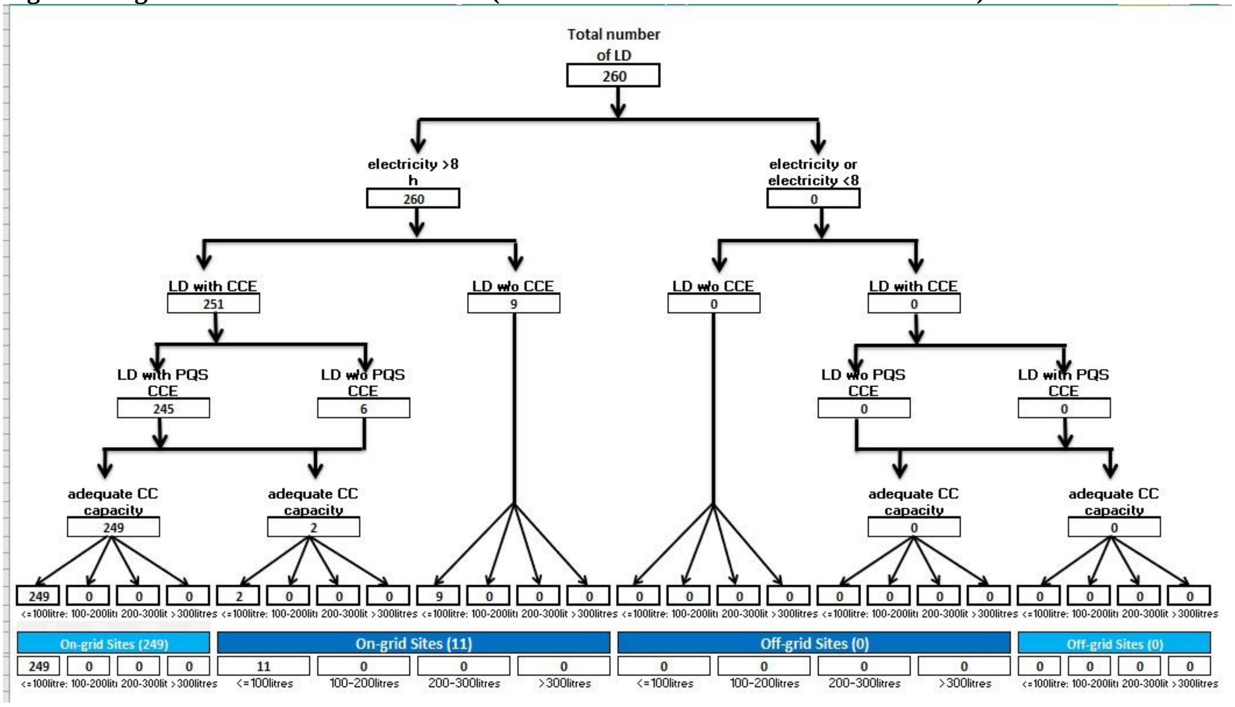

8.1.5 Service delivery points

With the deployment of the CCEOP, the country still has 215 service delivery points that will require CCE of more or equal to 5 litres. Out of this, 10 do not have adequate electricity, hence would require SDD equipment. Only 205 would need electric powered refrigerators.

Figure 5: Segmentation of Health Facilities (Service Delivery Points – SP

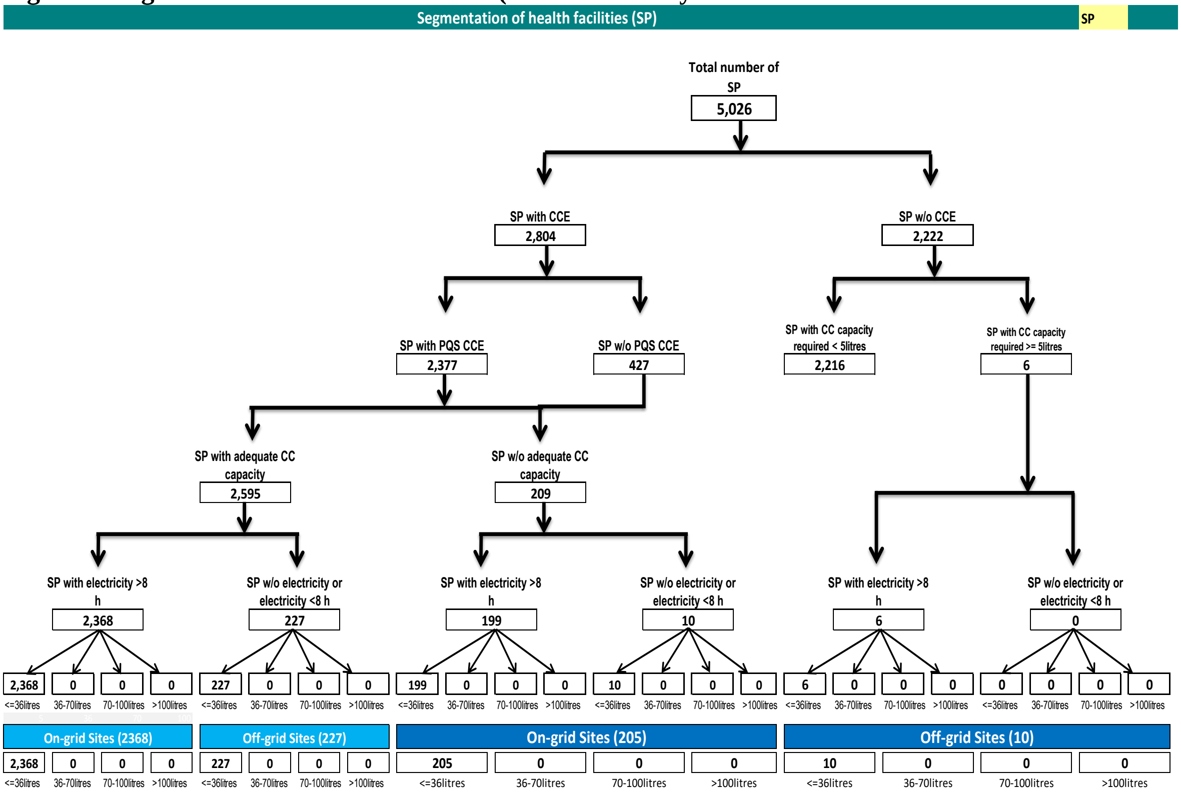

9.2 Dry Storage

There is adequate dry storage capacity at both national and the 10 old regions. The 6 new regions do not have dry storage space. Resources would therefore be required to establish dry stores in each of the new regions.

9.3 Transportation

For transportation purposes to achieve efficient deployment of vaccines from national to regions, an additional 2 cold vans (one -20°C and one -70°C) will be required.

Logistics distribution will be a decentralized activity, each level taking its share from the immediate upper level up to the districts. The districts will then distribute it to health facilities and vaccination sites.

The transport requirements for logistic distribution and as well as for campaign implementation will be mainly organized by each district by mobilizing resources from local governmental and non-governmental partners. Partners, particularly UNICEF and WHO, are also involved in providing support for transportation of vaccines and logistics to regions as required.

9.4 Vaccine Distribution Pattern

The movement of vaccine from the national level to the regions is illustrated in the diagram below. The six new regions are currently operating from their mother regional cold rooms. All vaccines will be delivered to regions by road using the cold vans. Regions will make similar distributions to districts using appropriate vehicles to maintain the cold chain.

Figure 6: Vaccine Distribution Paths

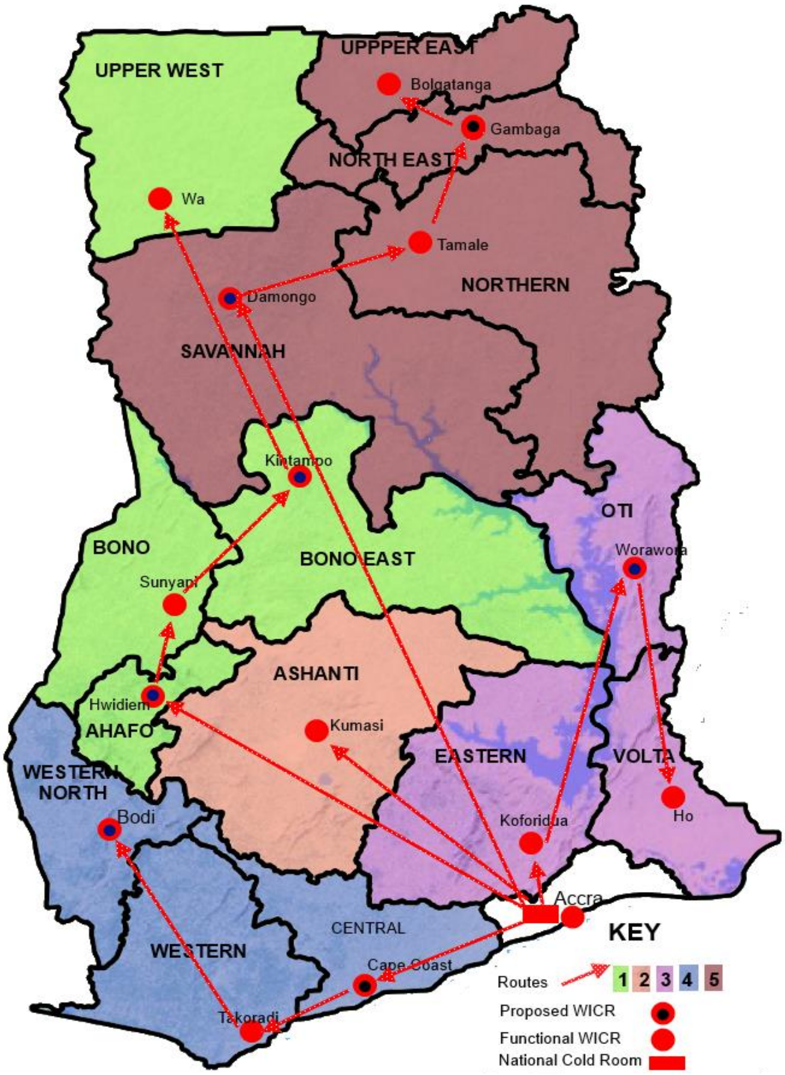

CHAPTER TEN:
WASTE MANAGEMENT

10.1 Policy on Injection Waste Management

According to the EPI policy on Injection Safety, the bundling principle is followed for the procurement and distribution of vaccines with auto disable (AD) syringes and safety boxes for the collection of injection sharps.

The final disposal of used syringes and sharps is done by incineration. Each district is required to have a functional incinerator. Currently, 47 districts do not have incinerators while 35 have incinerators that are dilapidated and hence not functional. So new incinerators would have to be constructed to replace the old ones. In total 82 would have to be constructed to support the COVID-19 vaccine deployment.

Only AD syringes with safety boxes will be used during the campaign and each vaccination site will be equipped with emergency kits including adrenalin. The waste will be incinerated in the incinerators at the district level. All the safety boxes shall be collected at the end of each session to the district level for incineration A plan will be prepared at the district level on collection of filled safety boxes and incineration at the district level.

Table 16: State of incinerators in 2020

Description	Quantity
Total number of Districts	260
Number of Districts with Incinerators	213
Number of Districts with dilapidated Incinerators	35
Number of Districts without Incinerators	47
Number of Incinerators to be constructed	82

CHAPTER ELEVEN:
EVALUATION AND TERMINATION

11.1 Evaluation

There will be a strong and urgent demand for data on COVID-19 vaccination by in-country and international stakeholders. The data team will lead the discussion on data needs and strengthen information systems to be able to provide fast, frequent and accurate reporting.

As we prepare for COVID-19 vaccine introduction we will use both traditional and electronic registration systems to provide estimates for each target population to be able to measure equitable coverage across different target populations.

The preparation of the campaign will be monitored based on the pre-designed checklist. Regular meetings will be conducted at all levels to monitor the preparations. Checklists will be prepared and used during and after the campaign. During the campaign, supervisors will conduct daily monitoring and supervision by observing vaccination posts and performances of teams. Supervisory checklists will be used as monitoring tools to collect necessary information and to take appropriate actions which improve the quality of the intervention. The supervisors will also be conducting intra campaign rapid convenient survey on each day of the campaign to validate coverage in areas that are already covered by teams.

At the end of the campaign, post campaign rapid convenient survey will be conducted in all districts by an independent monitoring institution as in the previous campaigns.

11.2 Termination of Deployment

Deployment operations would end with the completion of all vaccination activities. Health staff in areas where second doses would be administered need to plan to improve delivery of the second dose.

It is important for deployment operations to be terminated in a planned manner, resources returned to offices that deployed them, unused vaccines also returned to the National Cold Room and lessons learnt from the deployment documented for future emergency responses.

All supervisors would need to prepare and submit reports to the next level after end of vaccination activities. The reports will describe what would be done differently another time around and identify what went well and what should be changed to improve the efficiency of future responses. This information will be the basis of an overall consolidated country report on the response to the pandemic. The heads of facilities or administrative levels should complete all the data collection tools.

The National Coordination Committee upon the advice of the National Technical Coordinating Committee would then inform the public, national and international institutions involved in the

response that a particular phase of vaccination is over. In the event that the pandemic is over, similar processes would be used to communicate the termination of response measures.

CHAPTER TWELVE:
BUDGET AND FINANCING

12.1 Total budget

The total budget for operational activities is **Fifty-one million, six hundred and sixty-two thousand, two hundred and seventy-six United States dollars only (US$51,662,276 .00)** as presented in the table below (the detailed budget is attached):

No.	Category	Amount (GHS)	Amount (USD)
1	Coordination	12,875,525	2,258,864
2	Communication	4,532,120	795,109
3	Logistics and Waste Management	141,255,589	24,781,682
4	Training and Service Delivery	119,798,770	21,017,328
5	Data Management, Monitoring and Evaluation	6,236,960	1,094,204
6	Disease Surveillance and Safety Monitoring	9,313,600	1,633,965
7	Operational Research	462,410	81,125
	**TOTAL**	**294,474,974**	**51,662,276**
	Cedi-Dollar Exchange rate	5.7	

12.2 Financing

Parameter	Amount (USD)
Total budget	51,662,276
Funding (Source)	
Government	
World Bank	7,900,000
Gavi	
WHO	
UNICEF	
UNDP	
USAID	
CDC	
Private sector	
Funding gap	43,762,276

12.3 Strategy to address the budget gaps

The Ministry of Health in conjunction with the COVID-19 taskforce will complete stakeholder mapping, engage the identified stakeholders and advocate for stakeholder buy-in. The Ministry will then engage these stakeholders, development partners and private sector and present the funding gap and make an investment case for funding to support the vaccine deployment.

The Ministry will also leverage on the successes chalked with fund raising mechanism that supported the provision of resources and infrastructure for COVID-19 case management through the private sector fund to support vaccine procurement and operations.

ANNEXES

1-Plan of Action

Objectives	Activity	Time (Months) 2021				Implementers	Indicators
		Jan	Feb	Mar	Apr		
a) To receive COVID-19 vaccines and have them deployed to point of use	Procurement and Shipment Vaccines and other logistics	X				WHO/UNICEF	Vaccines procured and shipped on time
	Regulatory processes and Receipt of vaccines	X	X	X		Vaccine Deployment Sub Committee	Percent of sites receiving vaccines within 7 days of deployment
b) To disseminate Public Information	Radio and TV announcements	X	X	X	X	Communication Sub Committee	Number of radio and TV announcements
	Production of fact sheets, bulletins	X	X			Disease Surveillance Dept & Health Promotion Dept.	Number of bulletins and fact sheets produced
c) To vaccinate all target groups	Vaccination of target groups			X		District Health Directorates	Number of target groups immunized
	Good waste management practices	X				District Health Administrations	Number of districts with waste incinerated at close of day
e) To train all health workers involved in the deployment and vaccination of target groups	Planning meetings	X	X	X		National EPI	Planning meeting held<br>All health workers involved in vaccine deployment and vaccination trained
	Training of health workers, social mobilization, advocacy (Management and Organization)	X	X	X		National EPI, Reg. Health Administrations and District Health Administrations	
f) Post marketing surveillance	Conduction of AEFI activities	X	X	X	X	Disease Surveillance Dept. & National EPI	Number of reports submitted on AEFI activities

Annex II: Summary Budget

COVID_19 VACCIINE
DEPLOYMENT PLAN_
