# Supplementary material for: Barriers to the application of Health Technology Assessment (HTA) results: the case of COVID-19 vaccine deployment in Ghana
Source: Int J Technol Assess Health Care. 2026 Feb 2;42(1):e17. doi: 10.1017/S0266462325100342 (PMC12951341; doi:10.1017/S0266462325100342)
Supplement: Asare et al. supplementary material [file S0266462325100342sup001.zip › Supplementary Material 4_Policy Brief HTA COVID-19 costing.pdf]

Policy Brief

Cost Analysis of COVID-19 vaccinations in Ghana

perspectives from the Ghana Health Technology Assessment Structures

Introduction

In addition to the various measures put in place to control and prevent further spread of COVID-19 in Ghana, the Government of Ghana started implementation of the COVID-19 vaccination in March, 2021. Vaccination of the population is one of the strategic measures the country has adopted in line with global efforts to achieve herd immunity.

This Policy brief presents the analysis of the projected cost of COVID-19 vaccine introduction and deployment in Ghana. Using the COVID-19 Vaccine Introduction and Deployment Costing (CVIC) tool developed by World Health Organization (WHO) and United Nations Children's Fund (UNICEF).

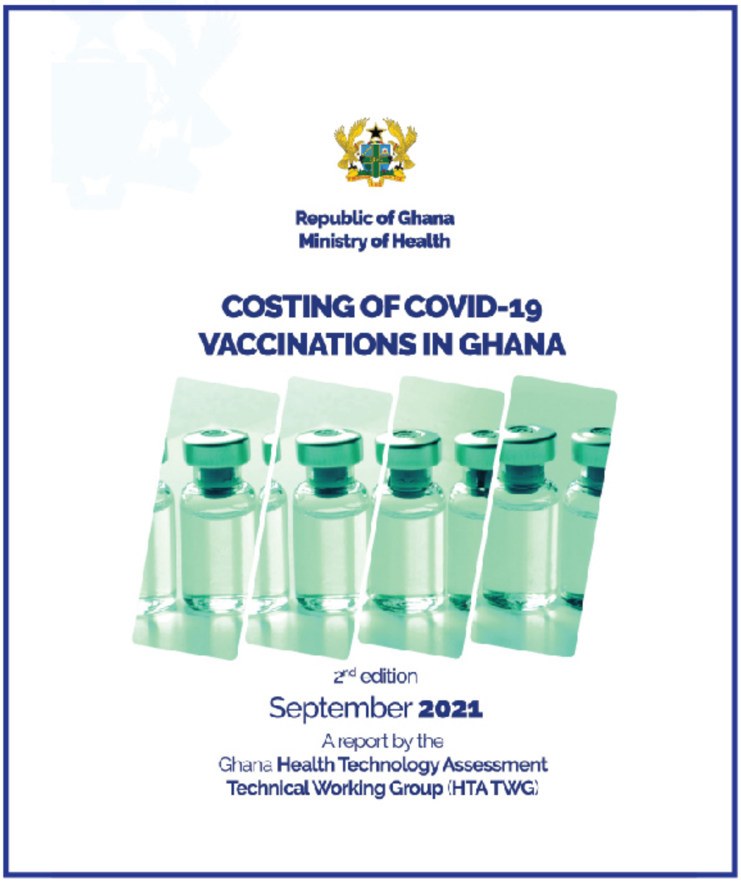

This work was made possible through the support of the World Bank.

SUMMARY

Key findings

The cost of COVID-19 vaccination ranges between **$349 to $436** million for the target population of **17.5 million.**

The majority (**78% to 83%**) of total cost above is due to the cost of vaccine doses, including shipping.

Between **8,437 to 10,247** vaccinators (non-FTEs) would be required during this period.

COVID-19 vaccine deployment and introduction is estimated to cost about **61% to 76%** of Ghana's 2021 health sector budget allocation for non-remuneration activities and projects.

Policy priorities

Containing financial risk on the health sector due to COVID-19

Contain vaccines costs in order to implement a sustainable vaccination programme with wide coverage.

What we did

The Technical Working Group for Health Technology Assessment (HTA-TWG), Ministry of Health, in collaboration with School of Public Health (SPH), University of Ghana (UG), collected data relevant for populating the CVIC tool through a technical workshop with various stakeholders. The data was validated with the Expanded Programme of Immunization (EPI) subsequently. Given prevailing global vaccine market and distribution, four main scenarios were analyzed taking into consideration various combinations of vaccines approved for use in Ghana and timelines. Upon further deliberations with the HTA Steering Committee, and Presidential advisors on COVID-19, three other scenarios were added, resulting in seven scenarios.

The scenarios included AstraZeneca (40%), J & J (30%), Moderna, Pfizer, and Sputnik V at 10% each; with full vaccination by second half of 2021 (Scenario 1). AstraZeneca (30%), J & J (40%), Moderna, Pfizer, and Sputnik V at 10% each with full vaccination by first half of 2022 (Scenario 2). There is equal distribution (20%) among AstraZeneca, J & J, Moderna, Pfizer, and Sputnik V; with full vaccination by second half of 2022.

Summary findings and policy implications

[1] In total, the cost of COVID-19 vaccination ranges between **$349 to $436** million for the target population of **17.5 million** (i.e., 57% of the population). These translate into cost per fully vaccinated person of **$21 to $26** and cost per dose including vaccine of **$10 to $13**. Again, cost per fully vaccinated person excluding vaccine cost between **$4.5 and $4.6**, thus cost per dose excluding vaccine cost also ranged from **$2.2 to $2.3**.

[2] The main cost driver is vaccine doses, including shipping, which accounts for between **78% to 83%** of total cost. This is followed by costs to be incurred on vaccinators, which is **8% to 10%** of total cost. Further, between **8,437 to 10,247** vaccinators (non-FTEs) would be required during this period.

[3] COVID-19 vaccine deployment and introduction is estimated to cost about **61% to 76%** of Ghana's 2021 health sector budget allocation for non-remuneration activities and projects. Efforts are required to mobilize the required resources to vaccinate the population against COVID-19, and these findings provide the estimates to inform resource mobilization efforts by government and other partners.

The implications for the policy processes could be a contraction of the funding space, for other priority policy actions within the Health sector unless innovative measuers are established to contain the financial and economic impact of COVID-19 vaccination on prioritization of policy action.

Considering that Vaccine Cost is the main cost driver for the entire vaccination programme, critical efforts must be made to contain vaccine cost, in order to ensure a sustainable vaccination programme, and wide coverage of the Ghanaian population.

Cost breakdown analysis under various scenarios

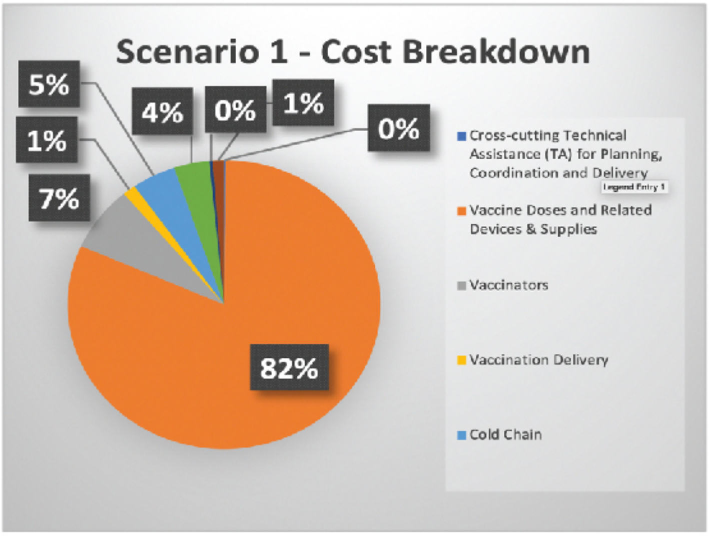

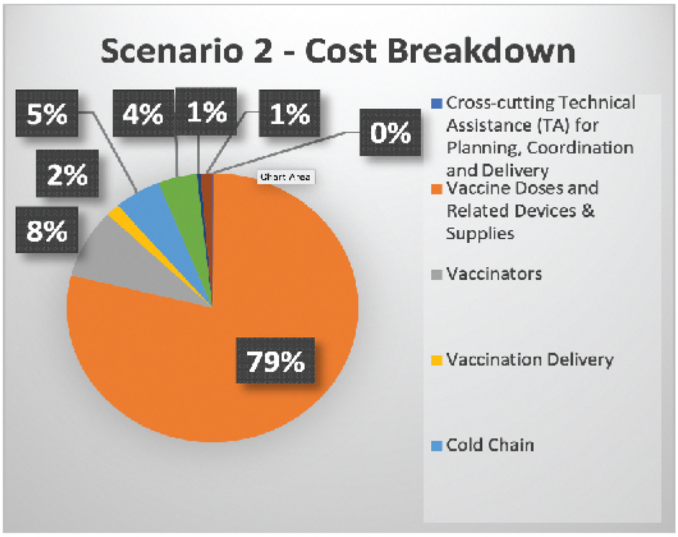

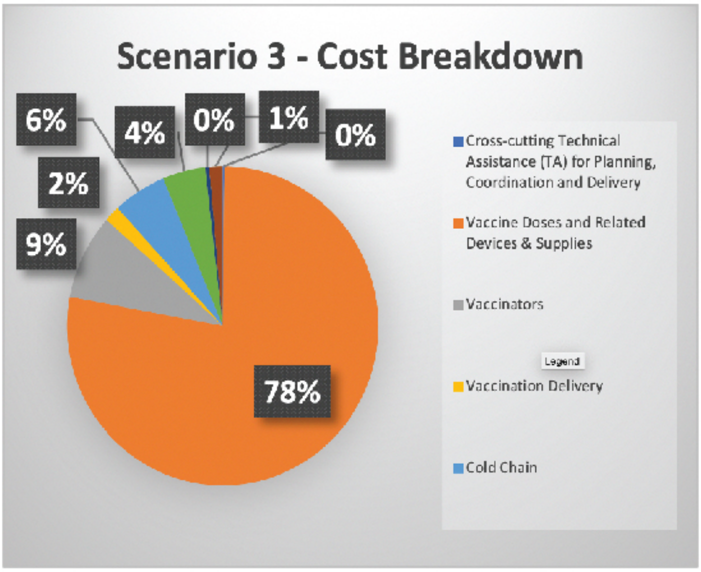

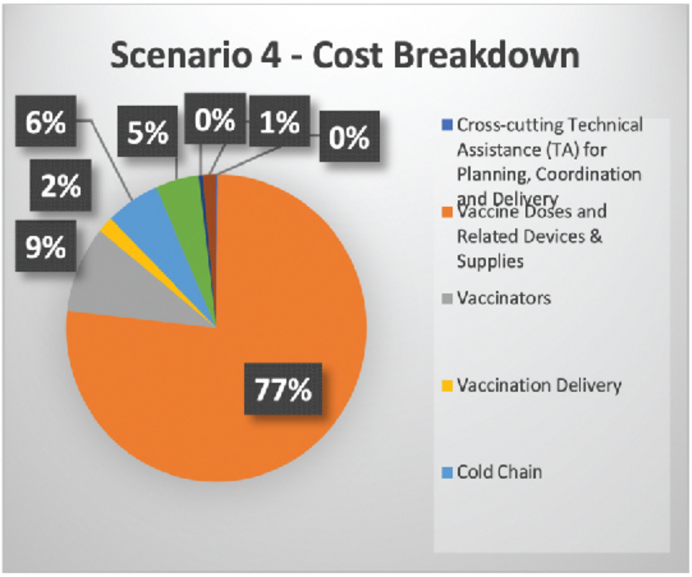

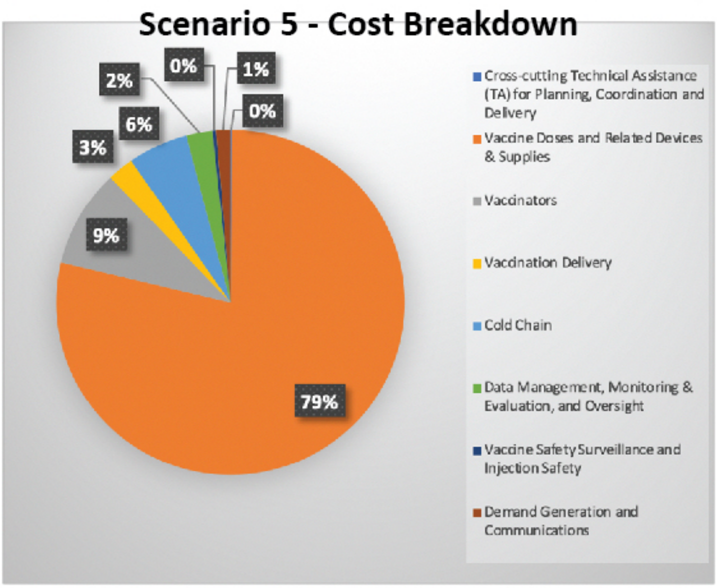

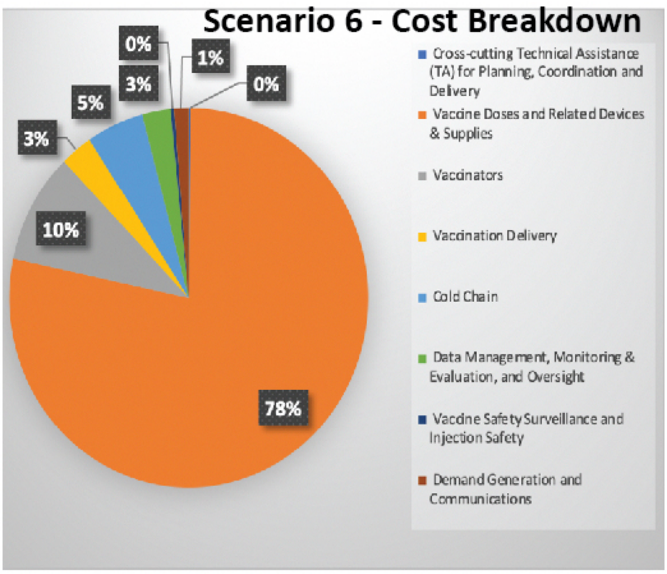

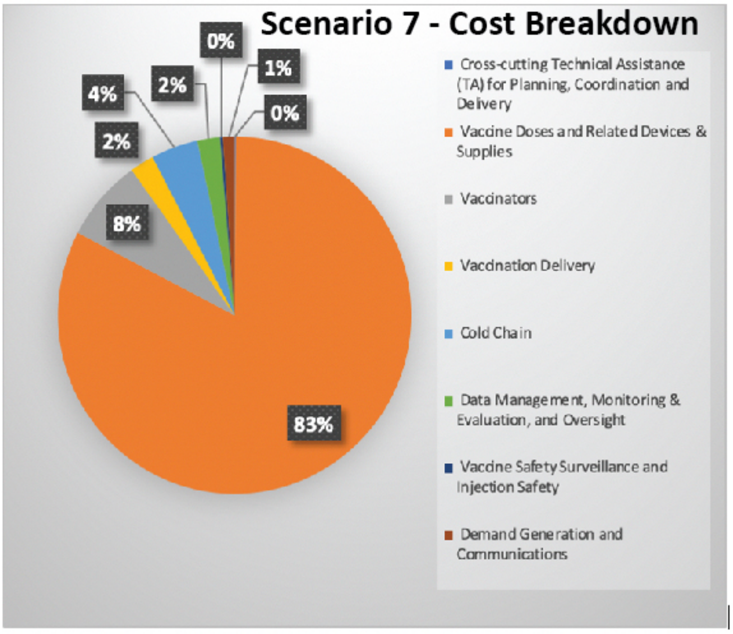

For further information, contact:

HTA Secretariat
Ministry of Health - Pharmacy Directorate
(Technical Coordination Directorate)
Sekou Toure Street, Accra

Conclusions

In conclusion, this result can be adopted by the Ministry of Health to support decision making regarding the introduction and deployment of COVID-19 vaccine in the country. Again, it will be an important guide for financing and sustainability of the COVID-19 vaccination programme.

Health Technology Assessment institutionalisation mechanism

The National Medicines Policy defined three entities to be tasked with the responsibility for ensuring the successful rollout of HTA in Ghana. Under the section on Health Technology Assessments, the governance structures for implementation of the Ghana HTA strategy 1st edition, shall be:
1, the HTA Steering Committee (responsible for governance),
2, the HTA Technical Working Group (responsible for technical functions) as well as
3, the HTA Secretariat (responsible for the support and management of all HTA work and processes in Ghana). The HTA Secretariat therfore works under the Pharmacy Directorate within the MOH structure for Technical Coordination.

HTA structures and processes would:
1. deliver evidence-based recommendations (as part of its mandate)
2. identify and appropriate policy implementation levers
3. provide recommendations on change management and implementation, informed by implementation research
4. develop impact assessment framework based on explicit criteria
5. assess impact of HTA implementation as part of health systems strengthening

The roll-out of HTA in Ghana has been through collaborations and partnerships with iDSI, NIPH, ADP/PATH, UG, UHAS, KNUST, NHIA, GHS, among others.

THE WORLD BANK
