# Supplementary material for: Barriers to the application of Health Technology Assessment (HTA) results: the case of COVID-19 vaccine deployment in Ghana
Source: Int J Technol Assess Health Care. 2026 Feb 2;42(1):e17. doi: 10.1017/S0266462325100342 (PMC12951341; doi:10.1017/S0266462325100342)
Supplement: Asare et al. supplementary material [file S0266462325100342sup001.zip › Supplementary Material 5 Key messages from the Costing of covid19 vaccines.docx]

**Key messages from the Cost-analysis of COVID-19 deployment plan** (1)

1. The estimated total cost of COVID-19 vaccination ranges between $348.7 and $436.1 million for the target population of 17.5 million.
2. These translate into per person completed primary schedule cost of $20.9–$26.2 and per dose (including vaccine cost) of $10.5–$13.1.
3. Again, per person completed primary schedule excluding vaccine cost was $4.5 and $4.6, thus per dose excluding vaccine also ranged from $2.2 – $2.3.
4. The main cost driver was vaccine doses, including shipping, which accounts for between 78% and 83% of total cost.
5. Further, an estimated 8,437–10,247 vaccinators (non-FTEs) would be required during 2021–2022 to vaccinate using a mix of delivery strategies, accounting for 8–10% of total cost.

**Reference**

1. Nonvignon J, Owusu R, Asare B, Adjagba A, Aun YW, Yeung KHT, et al. Estimating the cost of COVID-19 vaccine deployment and introduction in Ghana using the CVIC tool. Vaccine [Internet]. 2022 Mar 15 [cited 2022 May 20];40(12):1879–87. Available from: http://www.ncbi.nlm.nih.gov/pubmed/35190206
