# Supplementary material for: Barriers to the application of Health Technology Assessment (HTA) results: the case of COVID-19 vaccine deployment in Ghana
Source: Int J Technol Assess Health Care. 2026 Feb 2;42(1):e17. doi: 10.1017/S0266462325100342 (PMC12951341; doi:10.1017/S0266462325100342)
Supplement: Asare et al. supplementary material [file S0266462325100342sup001.zip › Supplementary Material 6 Consent Form and interview guide.docx]

**Consent Form** (UHAS-REC Consent form)

**
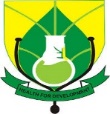
**
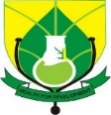
**RESEARCH OPERATIONS OFFICE**

**INSTITUTE OF HEALTH RESEARCH**

**UNIVERSITY OF HEALTH AND ALLIED SCIENCES**

**RESEARCH ETHICS COMMITTEE (REC)**

**PROTOCOL CONSENT FORM**

| Section A- **BACKGROUND INFORMATION** |
| --- |

| Title of Study: | **Identifying and resolving the factors responsible for non-application of the outcomes of Health Technology Assessment (HTA) on COVID-19 vaccines in Ghana.** |
| --- | --- |
| Principal Investigator: |  |
| Certified Protocol Number |  |

| Section B– **CONSENT TO PARTICIPATE IN RESEARCH** |
| --- |

**General Information about Research**

The World Health Organization and UNICEF introduced a costing tool that aims at guiding countries that seek to introduce COVID-19 vaccines. The COVID-19 Vaccine Introduction and deployment Costing tool (CVIC tool) is used to estimate the incremental costs for resource mobilization purposes including the World Bank’s COVID-19 Fast-Track Loan Facility (WHO, 2021). Countries can also use the tool to prepare budgets for vaccination beyond 2021 as COVID-19 vaccine is deployed. The tool aligns with the COVID-19 vaccine introduction readiness assessment tool, the guidance on developing a national deployment and vaccination plan, and the WHO SAGE values framework for the allocation and prioritization of COVID-19 vaccination.

The Ministry of Health’s Technical Working Group for Health Technology Assessment (HTA-TWG) used the CVIC tool to estimate the cost and economic burdens of the various COVID-19 vaccines and vaccination programmes available at the time, in a rapid HTA.

The results of this activity were presented to the special session of the Ghana HTA Steering Committee with the Presidential Advisors on Health and COVID-19, and the WHO Country Representative. It was observed that after the results of this work were disseminated, implementing stakeholders did not apply it in the selection of vaccines to be procured. This study, however, seeks to identify and resolve the reasons for the non-application of the outcomes of Health Technology Assessment (HTA) on COVID-19 vaccines by implementing stakeholders in Ghana. At the end of research, expectations are to identify solutions to address the bottlenecks to the application of HTA results and develop strategies for effective uptake of the results.

**Benefits of the study**

There will be no direct benefit to any participant, however your participation will help us find out more about how to ensure the effective uptake of HTA recommendations, which would inure to the benefit of society at large.

**Risks of the study**

We are asking you to share with us information about what you think caused the non-application of the HTA results, which may be confidential, and you may feel uncomfortable answering some of the questions. You do not have to answer any question or take part in the study if you do not wish to do so. You do not have to give us any reason for not responding to any question or refusing to take part in the interview.

**Confidentiality**

By participating in this study, you agree for your responses to be analysed and the results presented in anonymous form. In-depth interview or focus group discussion is likely to be audio taped. You understand the results of this study will be used by the research team to develop interventions to improve on uptake of HTA results in Ghana, present at conferences and published in peer reviewed journals. Your name will not be used in this study and data will be anonymized.

**Compensation**

Participants will not receive any compensation for participating in this study.

**Withdrawal from Study**

Your participation is voluntary and participants may withdraw at any time without penalty. You will not be adversely affected if you decline to participate or later stop participating.

Your legal representative will be informed in a timely manner if information becomes available that may be relevant to your willingness to continue participation or withdraw.

**Contact for Additional Information**

The Principal Investigator is Ms. Edith Gavor, Ministry of Health, P.O. Box M 44 Sekou Toure Avenue, North Ridge Accra, Ghana. Questions should be directed to **Dr. Brian Asare, ph. +233244529867,** [basare100@gmail.com](mailto:basare100@gmail.com)

If you have any questions about your rights as a research participant in this study you may contact the Administrator of the Research Ethics Committee, IHR, University of Health and Allied Sciences at [**rec@uhas.edu.gh**](mailto:rec@uhas.edu.gh)**or +233- 362-196-193**.

| Section C- **PARTICIPANT AGREEMENT** |
| --- |

**"I have read or have had someone read all of the above, asked questions, received answers regarding participation in this study, and am willing to give consent for me, my child/ward to participate in this study. I will not have waived any of my rights by signing this consent form. Upon signing this consent form, I will receive a copy for my personal records."**

________________________________________________

Name of Participant

_________________________________________________ _______________________

Signature or mark of Participant Date

**If participant cannot read and or understand the form themselves, a witness must sign here:**

I was present while the benefits, risks and procedures were read to the volunteer. All questions were answered and the volunteer has agreed to take part in the research.

_________________________________________________

Name of witness

____________________________________________ _______________________

Signature of witness / Mark Date

I certify that the nature and purpose, the potential benefits, and possible risks associated with participating in this research have been explained to the above individual.

__________________________________________

Name of Person who Obtained Consent

________________________________________ ______________________

Signature of Person Who Obtained Consent Date

**Data Collection Instruments**

Identifying and resolving the factors responsible for the non-application of the outcomes of Health Technology Assessment (HTA) on COVID-19 vaccines in Ghana

**Interview Guide**

| Roles and functions   1. What is your role in relation to COVID-19 vaccines in Ghana?    1. Can you describe what you do in relation to COVID-19 vaccines?    2. Are you responsible for any decisions on COVID-19 vaccines? |
| --- |
| Knowledge about HTA |
| 1. What is your general knowledge about HTA in Ghana? 2. What is the relevance of HTA in vaccine selection and deployment? |
| Awareness and knowledge about the HTA costing report   1. What do you know about the health technology assessment costing analysis done on the COVID-19 vaccines in Ghana? (If respondent does not know about the report, skip to question 7)    1. Kindly tell me about the HTA analysis conducted    2. How did you know about it?    3. When did you hear about the report?    4. How can one access the report? (soft or hard copy) 2. Kindly tell me about the report    1. Can you describe briefly the findings of the report?    2. Can you describe briefly the recommendations from that report? |
| Application of findings   1. Can you identify any instance or instances where any aspect of the report has been relevant for your work? **(this is apart from the main recommendations of the report)**    1. How does this report relate to your work as a …    2. Which aspects of your work did you apply this report to?    3. What decisions have been made regarding the report (in presentations, use in advocacy, conferences, meeting reports etc)? Kindly give examples    4. What were the challenges in the process of using these aspects of the report?    5. What enabled you to use these aspects of the report?    6. What were the positive outcomes from the use of the report?    7. What were the negative outcomes from the use of the report? |
| Issues affecting the use of the report   1. What do you think are some of the **factors** that would **prevent (make it difficult for) the application** of the recommendations of the report or such kinds of reports in the future?    1. What do you think are the considerations (e.g. the political factors, political will) that could negatively affect the application of such reports?    2. What do you think are the economic factors (e.g. funding constraints) that could negatively affect the application of such reports?    3. What do you think are the health system factors (e.g. unavailability of appropriate fridges for certain types of vaccines) that could negatively affect the application of such reports? 2. What do you think are some of the **factors** that would **enhance your application** of the recommendations of the report or such kinds of reports in the future?    1. In your opinion what are the political factors (e.g. political will) that could **enhance** the application of such reports?    2. Kindly tell me about the economic factors (e.g. availability of funding) that could **enhance** the application of such reports?    3. What do you think are the health system factors (e.g availability of appropriate fridges for certain types of vaccines) that could **enhance** the application of such reports? |
| **Any other comments**  **Kindly share any information I may have missed in this interview** |
