# Supplementary material for: Barriers to the application of Health Technology Assessment (HTA) results: the case of COVID-19 vaccine deployment in Ghana
Source: Int J Technol Assess Health Care. 2026 Feb 2;42(1):e17. doi: 10.1017/S0266462325100342 (PMC12951341; doi:10.1017/S0266462325100342)
Supplement: Asare et al. supplementary material [file S0266462325100342sup001.zip › Supplementary Material 7 - Table 1 stakeholders and their roles.docx]

Table 1: Stakeholder groups and their roles

| Stakeholder | Role |
| --- | --- |
| Drug Policy Unit | Develops and oversees policies for drug use, selection, and procurement. Influences evidence-based decisions for drug-related health technologies, including vaccines. |
| HTA Secretariat, TWG and Steering committee | Conducts technical assessments and generates HTA evidence. The secretariat coordinates the generation and dissemination of HTA reports. |
| National Medicines Selection Committee | Reviews and selects medicines for inclusion in the Essential Medicines List. Integrate HTA recommendations into national medicines policies. |
| Policy Planning Monitoring and Evaluation Directorate | Monitors and evaluates the application of HTA evidence in health programs. |
| Procurement and Supply Directorate | Manages procurement processes for vaccines and other health commodities. |
| National Immunization Technical Advisory Group (NITAG) | Provides expert advice on immunization policy, including vaccine selection. |
| Ministry of Finance | Allocates funding for health programs, including vaccine procurement and deployment. |
