# Supplementary material for: Barriers to the application of Health Technology Assessment (HTA) results: the case of COVID-19 vaccine deployment in Ghana
Source: Int J Technol Assess Health Care. 2026 Feb 2;42(1):e17. doi: 10.1017/S0266462325100342 (PMC12951341; doi:10.1017/S0266462325100342)
Supplement: Asare et al. supplementary material [file S0266462325100342sup001.zip › Supplementary Material 8 - Table 2 Summary of findings and recommendations.docx]

Supplementary Material 7

Table 2: Key findings and messages (recommendations)

| Key findings | Key message (recommendations) |
| --- | --- |
| Technical Issues | |
| The timing of the release of the report and its accessibility to decision-makers significantly impact the implementation of the recommendations. | Reports should be released promptly and made readily accessible to all relevant stakeholders to ensure they can be effectively used in decision-making processes. |
| The technical complexity of the HTA report was a significant barrier to its implementation. Stakeholders, particularly non-technical ones, found it challenging to understand the report's findings due to its technical density. | There is a need to present findings in a manner that is easily understandable to a wide range of stakeholders, including those without technical expertise. |
| A mismatch between the content of the report and the decisions that need to be made can serve as a barrier to implementation. | Reports should align with the needs of decision-makers to ensure their relevance and applicability. |
| Political, organisation, and ethical issues | |
| Political considerations and power dynamics significantly influenced the implementation of the report's recommendations. Decisions were often made by politicians, not technicians, and power dynamics and differing interests could override evidence-based recommendations. | There is a need for a legal framework that supports the implementation of evidence-based recommendations and reduces the influence of politics on decision-making. |
| Health system fragmentation and poor responsiveness to research findings were identified as major barriers. Fragmentation within the health system hindered the effective implementation of recommendations, and decision-makers often failed to adequately acknowledge, act upon, or integrate research findings into their decision-making processes. | Efforts should be made to improve coordination, communication, and responsiveness within the health system. Decision-makers should be encouraged to acknowledge, act upon, and integrate research findings into their decision-making processes. |
