# Supplementary figures and images for: Barriers to the application of Health Technology Assessment (HTA) results: the case of COVID-19 vaccine deployment in Ghana
Source: Int J Technol Assess Health Care. 2026 Feb 2;42(1):e17. doi: 10.1017/S0266462325100342 (PMC12951341; doi:10.1017/S0266462325100342)

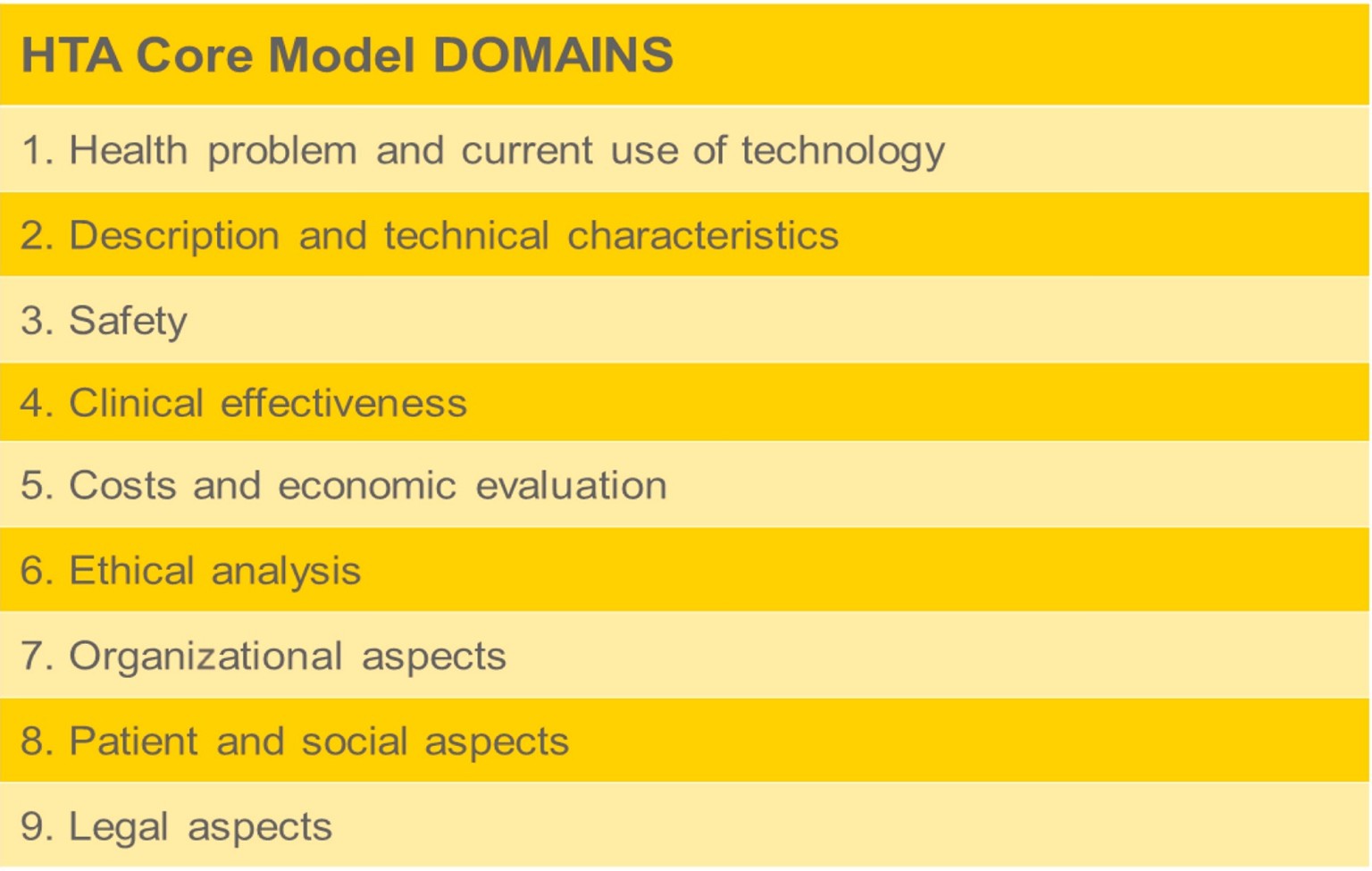

Supplement: Asare et al. supplementary material [file S0266462325100342sup001.zip › Supplementary figure 1 HTA Core Model Domains.jpg]
